# A Hitchhiker's Guide to Functional Magnetic Resonance Imaging

**DOI:** 10.3389/fnins.2016.00515

**Published:** 2016-11-10

**Authors:** José M. Soares, Ricardo Magalhães, Pedro S. Moreira, Alexandre Sousa, Edward Ganz, Adriana Sampaio, Victor Alves, Paulo Marques, Nuno Sousa

**Affiliations:** ^1^Life and Health Sciences Research Institute (ICVS), School of Medicine, University of MinhoBraga, Portugal; ^2^ICVS/3B's - PT Government Associate LaboratoryBraga, Portugal; ^3^Department of Informatics, University of MinhoBraga, Portugal; ^4^Neuropsychophysiology Lab, CIPsi, School of Psychology, University of MinhoBraga, Portugal; ^5^Clinical Academic Center – BragaBraga, Portugal

**Keywords:** fMRI, hitchhiker's guide, acquisition, preprocessing, analysis

## Abstract

Functional Magnetic Resonance Imaging (fMRI) studies have become increasingly popular both with clinicians and researchers as they are capable of providing unique insights into brain functions. However, multiple technical considerations (ranging from specifics of paradigm design to imaging artifacts, complex protocol definition, and multitude of processing and methods of analysis, as well as intrinsic methodological limitations) must be considered and addressed in order to optimize fMRI analysis and to arrive at the most accurate and grounded interpretation of the data. In practice, the researcher/clinician must choose, from many available options, the most suitable software tool for each stage of the fMRI analysis pipeline. Herein we provide a straightforward guide designed to address, for each of the major stages, the techniques, and tools involved in the process. We have developed this guide both to help those new to the technique to overcome the most critical difficulties in its use, as well as to serve as a resource for the neuroimaging community.

## Introduction

Introduced in the early nineties, functional Magnetic Resonance Imaging (fMRI) (Bandettini et al., 1992; Kwong et al., 1992; Ogawa et al., 1992; Bandettini, 2012a; Kwong, 2012) is a variant of conventional Magnetic Resonance Imaging (MRI) intended to measure brain activity and connectivity. It is a fundamentally non-invasive method, and one which provides a method to assess brain function with unparalleled spatial specificity. Amongst its attributes are high spatial resolution, signal reliability, robustness, and reproducibility.

**Figure 1:**
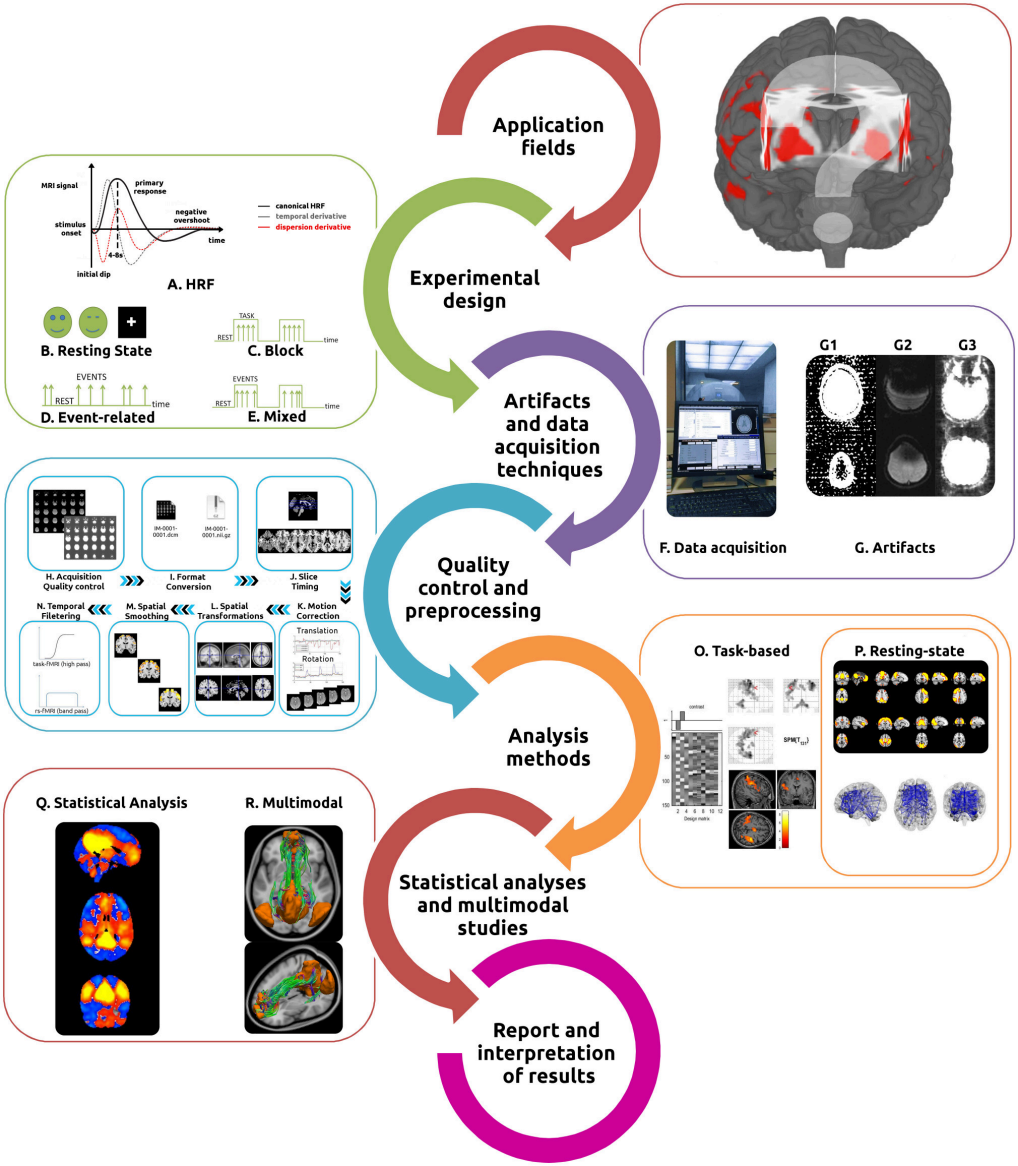
**Typical fMRI workflow**. In order to perform the most appropriate fMRI study (either task-based or resting state), researchers/clinicians need to understand its main application fields, intrinsic hemodynamic characteristics **(A)** and how to best design the experiment [Resting State **(B)**, Block **(C)**, Event related **(D)**, or Mixed **(E)** designs]. Identification of the most appropriate acquisition techniques **(F)** and the recognition of the primary artifacts involved **(G)** are essential. The acquired data then undergoes several quality control and preprocessing steps [acquisition quality control **(H)**, format conversion **(I)**, slice timing **(J)**, motion correction **(K)**, spatial transformations **(L)**, spatial smoothing **(M)**, and temporal filtering **(N)**]. The intended analysis methods should be implemented for task-based **(O)** or resting-state fMRI **(P)** and statistical inferences performed **(Q)**. Analysis can be complemented with a variety of different methods for multimodal studies **(R)**. Finally, results interpretation should be made with extreme caution.

Functional brain mapping is most commonly performed using the venous blood oxygenation level-dependent (BOLD) contrast technique (Ogawa and Lee, 1990; Ogawa et al., 1990a,b; Ogawa, 2012). The magnitude of the BOLD signal is an indirect measure of neuronal activity, and is a composite which reflects changes in regional cerebral blood flow, volume, and oxygenation. Functional MRI principles and basic concepts have been extensively described and reviewed in the literature (Le Bihan, 1996; Gore, 2003; Amaro and Barker, 2006; Norris, 2006; Logothetis, 2008; Buxton, 2009; Faro and Mohamed, 2010; Ulmer and Jansen, 2010; Poldrack et al., 2011; Bandettini, 2012b; Uğurbil and Ogawa, 2015). In summary, the basic concept underlying all fMRI measurement is that an increase in local neuronal activity stimulates both higher energy consumption and increased blood flow. The resultant indirect determination of brain function is typically represented as a statistical map which reflects regional activity. Information transfer between neurons is a metabolically demanding process, which requires an increased flow of oxygenated blood, oxyhemoglobin. The local influx of oxygenated blood results in a net increase in the balance of oxygenated arterial blood to deoxygenated venous blood (associated with elevated deoxyhemoglobin). The increase in the oxy-/deoxy-hemoglobin ratio leads to an increase in the MRI signal compared to that of the surrounding tissue. It is important to note that as local neuronal activity increases, there is an intrinsic delay before regional vasodilation occurs and flow increases. This mechanism, which is a function of the properties of the local vascular network, is referred to as the hemodynamic response function (HRF) and has a time course of several seconds after the increase in activity. The BOLD signal can be characterized by the shape of this HRF, which reflects its vascular origin. Typically, fMRI software model the HRF with a set of gamma functions, commonly designated by canonical HRF, that is characterized by a gradual rise, peaking ~5–6 s after the stimulus, followed by a return to the baseline (about 12 s after the stimulus) and a small undershoot before stabilizing again, 25–30 s after (Figure 1A) (Miezin et al., 2000; Buxton et al., 2004; Handwerker et al., 2012). Occasionally, an initial dip is reported but its origin and implications are still under debate (Hu and Yacoub, 2012). This also highlights that, despite the good fit of the canonical HRF for most situations, the true HRF is known to present some variability. Whenever a researcher suspects that the canonical HRF is not good enough, it is common practice to include its temporal and dispersion derivatives in the model in order to estimate the variability in latency and shape, respectively (Friston et al., 1998; Calhoun et al., 2004).

It has become an established practice in fMRI studies to investigate the differential neuronal responses to various forms of stimuli and activity during task performance. Typical investigations have compared periods of brain activation during a task with periods of a matched baseline task or a “rest” condition (Bandettini et al., 1992; Blamire et al., 1992; Vallesi et al., 2015). However, stimulus-evoked responses are only the tip of the iceberg in brain activity. More recently, a new perspective in functional imaging has brought with it the recognition that spontaneous/intrinsic brain activity is a fundamental aspect of normal brain function. Technical advances in neuroimaging methods have contributed to this paradigm shift, and have led to the recognition that the brain is more accurately considered a network of functionally connected (co-varying) and constantly interacting regions, requiring a focus on understanding patterns of connectivity as well as localized activation (Biswal et al., 1995; Carlson et al., 2003; Fox et al., 2005; Fox and Raichle, 2007; Raichle, 2009; Smith et al., 2011). For this reason, resting state fMRI (rs-fMRI) analyses rely upon spontaneous coupled brain activity to reveal intrinsic signal fluctuations in the absence of external stimuli or demands of imposed tasks (Damoiseaux et al., 2006; Fox and Raichle, 2007; Schölvinck et al., 2010; van den Heuvel and Hulshoff Pol, 2010; Friston et al., 2014b). Complimentary approaches, combining rs-fMRI with functional deactivation (shifting from periods of stimulation to those of rest) also have been described to study functional activity transitions (Greicius and Menon, 2004; Anticevic et al., 2012; Soares et al., 2016).

With its popularity steadily increasing among clinicians and researchers, the technique of fMRI has demonstrated great utility in the study of the functioning brain, both in health and disease. It is important to recognize, however, that it has an intrinsically complex workflow (summarized in Figure 1) which assumes broad knowledge of task design, imaging artifacts, complex MRI acquisition techniques, a multitude of preprocessing and analysis methods (in several software packages see Tables 1–4), statistical analyses, as well as interpretation of results. Several papers and books describing the main technical issues and pitfalls related to both intrinsic and evoked activity have been published (Jezzard and Song, 1996; Le Bihan, 1996; Norris, 2006; Haller and Bartsch, 2009; Cole et al., 2010; Margulies et al., 2010; Poldrack et al., 2011; Davis and Poldrack, 2013; Lee et al., 2013; Uğurbil and Ogawa, 2015). However, given the complex nature of the data processing, constant methodological advances and the increasingly broad application of fMRI to both the clinical and research domains, we have sought to compile a practical “hitchhiker's guide,” containing essential information and primary references. These guides have proven to be important to assist in the optimization of data quality and interpretation of results (Soares et al., 2013). We also have provided an analysis of the principal software tools available for each step in the workflow, highlighting the most suitable features of each. Through this process it is our goal to enable investigators/clinicians to design and implement practical workflows which will lead to robust and reproducible results. In the following sections, information about each specific fMRI workflow step, from the current technique applications to the final results interpretation, will be discussed in detail. We have started by presenting a list of common software tools used for fMRI pipelines (Table 1), including both applications for general and wide-ranging purposes (e.g., AFNI, BrainVoyager, FSL, or SPM) as well as for very specific tasks (e.g., Marsbar and NBS).

**Table 1:** **Software tools used for fMRI pipelines present in published studies**.

**Software tools**	**URL**	**Main purpose**
Analysis of Functional NeuroImages (AFNI; Cox, 1996, 2012)	https://afni.nimh.nih.gov/afni/	Preprocessing, analysis, and statistical analysis
Analyze 4D	http://analyze4d.com/	Region of interest and time-series analysis
AnalyzeFMRI (Bordier et al., 2011)	https://cran.r-project.org/web/packages/AnalyzeFMRI/	Independent component analysis
BioImage Suite (Papademetris et al., 2006)	http://bioimagesuite.yale.edu/	Preprocessing, analysis, and statistical analysis (modules from AFNI)
Brain Connectivity Toolbox (Rubinov and Sporns, 2010)	http://www.brain-connectivity-toolbox.net/	Graph theory analysis
BrainVISA	http://brainvisa.info/web/index.html	Analysis and statistical analysis (preprocessing modules from SPM or FSL)
BrainVoyager (Goebel, 2012)	http://www.brainvoyager.com/	Preprocessing, analysis, and statistical analysis
BROCCOLI (Eklund et al., 2014)	https://github.com/wanderine/BROCCOLI/	Preprocessing, analysis, and statistical analysis (mainly non parametric) with GPU implementation
Cambridge Brain Analysis (CamBA)	http://www.bmu.psychiatry.cam.ac.uk/software/	Analysis and statistical analysis
Cambridge Centre for Ageing and Neuroscience (Cam-CAN)	http://www.cam-can.org/	Structural equation modeling analysis
Functional connectivity toolbox (CONN)	https://www.nitrc.org/projects/conn/	Preprocessing, analysis, and statistical analysis
Cosmo multi-variate pattern analysis toolbox (CosmoMVPA)	http://cosmomvpa.org/	Multi-voxel pattern analysis
Configurable Pipeline for the Analysis of Connectomes (C-PAC)	https://fcp-indi.github.io/	Preprocessing, analysis, and statistical analysis (based on AFNI, FSL, and ANTS)
Data Processing and Analysis for Brain Imaging (DPABI; Yan et al., 2016)	http://rfmri.org/dpabi	Preprocessing, analysis, and statistical analysis
Data Processing Assistant for Resting-State fMRI (DPARSF; Yan and Zang, 2010)	http://rfmri.org/DPARSF	Preprocessing and analysis
A software for dynamic functional connectivity analysis of fMRI data (DynaConn)	http://softwarecircuits.weebly.com/dynaconn.html	Dynamic functional connectivity
Dynamic brain connectome (DynamicBC; Liao et al., 2014)	http://restfmri.net/forum/DynamicBC	Dynamic functional connectivity
fMRI Advanced Normalization Tools (ANTsR; Avants et al., 2009)	http://stnava.github.io/fMRIANTs/	Preprocessing, analysis, and statistical analysis
FMRLAB	http://sccn.ucsd.edu/fmrlab/	Independent component analysis
Freesurfer (FSFAST; Fischl, 2012)	http://freesurfer.net/	Preprocessing, analysis, and statistical analysis
FMRIB Software Library (FSL; Jenkinson et al., 2012)	http://fsl.fmrib.ox.ac.uk/fsl/fslwiki/	Preprocessing, analysis, and statistical analysis
Group ICA Of fMRI Toolbox (GIFT; Calhoun et al., 2009)	http://mialab.mrn.org/software/gift/	Independent component analysis
Group Iterative Multiple Model Estimation (GIMME; Gates and Molenaar, 2012)	https://www.nitrc.org/projects/gimme/	Structural equation modeling analysis
GLM Flex	http://mrtools.mgh.harvard.edu/	Statistical analysis
Granger Multivariate Autoregressive Connectivity (GMAC; Tana et al., 2012)	https://www.nitrc.org/projects/gmac_2012/	Preprocessing, analysis (Granger causality mapping)
Generalized psychophysiological interactions (GPPI; McLaren et al., 2012)	https://www.nitrc.org/projects/gppi	Psychophysiological interactions analysis
A user-friendly toolbox for comprehensive graph analyses of functional brain connectivity (GraphVar; Kruschwitz et al., 2015)	http://rfmri.org/GraphVar	Graph theory analysis
GRaph thEoreTical Network Analysis (GRETNA; Wang J. et al., 2015)	https://www.nitrc.org/frs/shownotes.php?release_id = 2213	Graph theory analysis
Graph Theory GLM (GTG)	https://www.nitrc.org/projects/metalab_gtg/	Preprocessing and graph theory analysis
Marsbar	http://marsbar.sourceforge.net/	Region of interest analysis
Multivariate Granger causality (MVGC; Barnett and Seth, 2014)	http://www.sussex.ac.uk/sackler/mvgc/	Granger causality mapping analysis
Network Based Statistic (NBS; Zalesky et al., 2010)	https://www.nitrc.org/projects/nbs/	Graph theory analysis and statistical analysis
NeuroLens	https://www.nitrc.org/projects/nldo/	Preprocessing, analysis, and statistical analysis
Nipy (Millman and Brett, 2007)	http://nipy.org/	Preprocessing, analysis, and statistical analysis
Nitime	http://nipy.org/nitime/	Time-series analysis
Pattern Recognition for Neuroimaging Toolbox (PRONTO)	http://www.mlnl.cs.ucl.ac.uk/pronto/prtsoftware.html	Multi-voxel pattern analysis
MultiVariate Pattern Analysis in Python (PyMVPA; Hanke et al., 2009)	http://www.pymvpa.org/	Multi-voxel pattern analysis
Structural equation modeling for fMRI (SEM)	http://dslink333.dyndns.org/SEM.htm	Structural equation modeling analysis (toolbox for SPM)
Statistical non Parametric Mapping (SnPM; Nichols and Holmes, 2002)	http://www2.warwick.ac.uk/fac/sci/statistics/staff/academic-research/nichols/software/snpm	Non-parametric permutation/randomization analysis (toolbox for SPM)
Statistical Parametric Mapping (SPM; Friston et al., 2007)	http://www.fil.ion.ucl.ac.uk/spm/	Preprocessing, processing and statistical analysis
The Decoding Toolbox (Hebart et al., 2014)	http://www.bccn-berlin.de/tdt	Multi-voxel pattern analysis (toolbox optimized for SPM)

**Table 2:** **Most common software tools used to program and present fMRI stimuli**.

**Software tools**	**URL**	**OS**	**GUI**	**Programming skills required**	**Availability**
A Simple Framework (ASF) (Schwarzbach, 2011)	https://code.google.com/p/asf/	Windows (Matlab)	×	✓	Open-source
BOLDSync (Joshi et al., 2014)	http://www.nbrc.ac.in/faculty/pravat/BOLDSync.php	Windows, Linux (Matlab)	✓	×	Open-source
Cogent 2000	http://www.vislab.ucl.ac.uk/cogent_2000.php	Windows (Matlab)	×	✓	Open-source
E-Prime (Psychology Software Tools, Pittsburgh, PA)	http://www.pstnet.com/	Windows	✓	×	Commercial
FLXLab	http://flxlab.sourceforge.net/	Windows, Linux, OS X	×	✓	Open-source
Inquisit	http://www.millisecond.com/	Windows, OS X	✓	✓	Commercial
NordicAktiva	http://www.nordicneurolab.com/	Windows	✓	×	Commercial
Paradigm	http://www.paradigmexperiments.com/	Windows	✓	×	Commercial
Presentation® (Neurobehavioral systems)	https://www.neurobs.com/	Windows	✓	✓	Commercial
Psychophysics Toolbox (Brainard, 1997)	http://psychtoolbox.org/	Windows, Linux, OS X (Matlab/Octave)	×	✓	Open-source
PsychoPy (Peirce, 2008)	http://www.psychopy.org/	Windows, Linux, OS X	✓	✓	Open-source
PsyScope X (Cohen et al., 1993)	http://psy.ck.sissa.it/	OS X	✓	✓	Open-source
Stim 2	http://compumedicsneuroscan.com/	Windows	✓	×	Commercial
SuperLab	http://www.superlab.com/	Windows, OS X	✓	×	Commercial
Wake Forest Visual Experimentation Software (WaVE) (Meyer and Constantinidis, 2005)	http://www.wakehealth.edu/Research/Neurobiology-and-Anatomy/Wake-Forest-Visual-Experimentation-Software-(WaVE).htm	Windows (Matlab)	✓	✓	Open-source

**Table 3:** A list of the main preprocessing steps implemented by the common fMRI tools ^*^.

**Software**	**Preprocessing steps**									
	**DICOM import**	**Slice Timing**	**Motion Correction**	**Motion outlier detection**	**Automated skull striping**	**Coregister**	**Normalization**	**Confound removal**	**Spatial Smoothing**	**Temporal Filtering**
AFNI	✓	✓	✓	✓	✓	✓	✓	✓	✓	✓
BioImage Suite	×	✓	✓	×	✓	✓	✓	✓	✓	✓
BrainVoyager	✓	✓	✓	×	✓	✓	✓	×	✓	✓
BROCCOLI	×	✓	✓	×	✓	✓	✓	✓	✓	✓
CONN	×	×	×	×	×	×	×	✓	×	✓
C-PAC	×	✓	✓	✓	✓	✓	✓	✓	✓	✓
DPABI	✓	✓	✓	✓	✓	✓	✓	✓	✓	✓
DPARSF	✓	✓	✓	✓	✓	✓	✓	✓	✓	✓
fMRIANTs/ANTsR	×	×	✓	×	×	✓	✓	✓	✓	×
Freesurfer	✓	✓	✓	×	✓	✓	✓	×	✓	×
FSL	×	✓	✓	✓	✓	✓	✓	✓	✓	✓
GMAC	×	×	×	×	×	×	×	✓	×	✓
GTG	×	✓	✓	✓	×	×	×	✓	×	✓
NeuroLens	✓	×	✓	×	×	✓	✓	×	✓	×
Nipy	✓	✓	✓	✓	✓	✓	✓	✓	✓	✓
SPM	✓	✓	✓	×	✓	✓	✓	✓	✓	✓

* *To the best of our knowledge at the date of submission, based on information gathered from the software manuals, main webpages and published papers*.

**Table 4:** A list of the main analysis methods implemented by the common fMRI tools^*^.

**Software**	**Task-based fMRI**						**Resting-state fMRI**							
	**GLM**	**PPI**	**SEM**	**DCM**	**GCM**	**MVPA**	**Seed-Based**	**ReHO**	**ALFF**	**PCA**	**ICA**	**Clustering**	**Graph Theory**	**dFC**
AFNI	✓	✓	✓	×	✓	×	✓	✓	✓	✓	✓	✓	×	×
AnalyzeFMRI	×	×	×	×	×	×	×	×	×	×	✓	×	×	×
BioImage Suite	✓	×	×	×	×	×	×	×	×	×	×	×	×	×
Brain Connectivity Toolbox	×	×	×	×	×	×	×	×	×	×	×	×	✓	×
BrainVoyager	✓	✓	×	×	✓	✓	✓	×	×	✓	✓	✓	×	×
BROCCOLI	✓	×	×	×	×	×	×	×	×	×	✓	×	×	×
CamBA	✓	×	×	×	×	×	×	×	×	×	×	×	×	×
Cam Can	×	×	✓	×	×	×	×	×	×	×	×	×	×	×
CONN	✓	✓	×	×	×	✓	✓	✓	×	✓	✓	×	✓	✓
CosmoMVPA	×	×	×	×	×	✓	×	×	×	×	×	×	×	×
C-PAC	×	×	×	×	×	×	✓	✓	✓	×	×	×	✓	×
DPABI	✓	×	×	×	×	×	✓	✓	✓	✓	×	×	×	×
DynaConn	×	×	×	×	×	×	×	×	×	×	×	×	×	✓
DynamicBC	×	×	×	×	×	×	×	×	×	×	×	×	×	✓
ANTs/ANTsR fMRI	✓	×	×	×	×	×	×	×	×	×	×	×	×	×
FMRLAB	×	×	×	×	×	×	×	×	×	×	✓	×	×	×
Freesurfer (FSFAST)	✓	×	×	×	×	×	×	×	×	×	×	×	×	×
FSL	✓	×	×	×	×	×	✓	×	✓	✓	✓	×	×	×
GIFT	×	×	×	×	×	×	×	×	×	×	✓	×	×	×
GIMME	×	×	✓	×	×	×	×	×	×	×	×	×	×	×
GLMFlex	✓	×	×	×	×	×	×	×	×	×	×	×	×	×
GMAC	×	×	×	×	✓	×	×	×	×	×	×	×	×	×
GPPI	×	✓	×	×	×	×	×	×	×	×	×	×	×	×
GraphVar	×	×	×	×	×	×	×	×	×	×	×	×	✓	×
GRETNA	×	×	×	×	×	×	×	×	×	×	×	×	✓	×
GTG	×	×	×	×	×	×	×	×	×	×	×	×	✓	×
Lipsia	✓	×	×	×	×	×	×	×	×	×	×	×	×	×
MVGC	×	×	×	×	✓	×	×	×	×	×	×	×	×	×
NBS	✓	×	×	×	×	×	×	×	×	×	×	×	✓	×
Neurolens	✓	×	×	×	×	×	×	×	×	×	×	×	×	×
Nipy	✓	×	×	×	×	×	×	×	×	×	×	×	×	×
Nitime	×	×	×	×	✓	×	✓	×	×	×	×	×	✓	×
PRONTO	×	×	×	×	×	✓	×	×	×	×	×	×	×	×
PyMVPA	×	×	×	×	×	✓	×	×	×	×	×	×	×	×
SEM - Structural Equation Modeling (SEM) for fMRI	×	×	✓	×	×	×	×	×	×	×	×	×	×	×
SnPM	✓	×	×	×	×	×	×	×	×	×	×	×	×	×
SPM	✓	✓	×	✓	×	×	✓	×	×	×	×	×	×	×
The Decoding Toolbox	×	×	×	×	×	✓	×	×	×	×	×	×	×	×

* *To the best of our knowledge at the date of submission, based on information gathered from the software manuals, main webpages and published papers*.

## Application fields

The use of the technique of fMRI has led to significant expansion of understanding in multiple areas of cognitive neuroscience (Cabeza, 2001; Raichle, 2001; Poldrack, 2008, 2012). It has, for example, been successfully used to study systems involved with sensory-motor functions (Biswal et al., 1995; Calvo-Merino et al., 2005), language (Woermann et al., 2003; Centeno et al., 2014), visuospatial orientation (Formisano et al., 2002; Rao and Singh, 2015), attention (Vuilleumier et al., 2001; Markett et al., 2014), memory (Machulda et al., 2003; Sidhu et al., 2015) affective processing (Kiehl et al., 2001; Shinkareva et al., 2014), working memory (Curtis and D'Esposito, 2003; Meyer et al., 2015), personality dimensions (Canli et al., 2001; Sampaio et al., 2013), decision-making (Bush et al., 2002; Soares et al., 2012), and executive function (Just et al., 2007; Di et al., 2014). Functional MRI has also been used as a tool in the study of topics as diverse as addiction behavior (Chase and Clark, 2010; Kober et al., 2016), neuromarketing (Ariely and Berns, 2010; Kuhn et al., 2016) and politics (Knutson et al., 2006), among others.

Another area in which fMRI is expanding is in clinical neuroimaging, with applications which range from pre-surgical mapping/planning (Stippich, 2015; Lee et al., 2016) to functional characterization of a variety of disease states (Matthews et al., 2006; Bullmore, 2012), as well as in understanding plasticity, contributing to the study of drug development (Wise and Preston, 2010; Duff et al., 2015), and in the study of genetically determined differences in function (Koten et al., 2009; Richiardi et al., 2015).

While these research and clinical-oriented fields of fMRI application usually require a task or stimulus evoked brain response, the defining attribute of not requiring active patient participation, triggered the use of rs-fMRI for additional research and clinical applications. This research methodology has been applied to prognostic and diagnostic information (Fox and Greicius, 2010; Lang et al., 2014), treatment guidance (Rosazza and Minati, 2011; Castellanos et al., 2013), identification of functional fingerprints, discovery, and validation of biomarkers in the investigation of relation to cognitive, emotional, and social processes (Fox M. D. et al., 2014; Krishnadas et al., 2014; Finn et al., 2015).

Finally, preliminary work appears to offer promise for the use of real-time neurofeedback and/or brain computer interfaces methodologies (Caria et al., 2012; Weiskopf, 2012; Sulzer et al., 2013; Kadosh et al., 2016), in the treatment of disorders such as Obsessive Compulsive Disorder (Emmert et al., 2016), Depression (Young et al., 2014), and Schizophrenia (Scheinost et al., 2013; Cordes et al., 2015).

## Experimental design

The number of variables (such as the specific nature of the research question, availability of imaging instruments, demand of data handling, and cost) associated with each study makes it essential to optimize BOLD signal acquisition time and statistical efficiency of the analysis. There is not one optimal design which will encompass all fMRI studies. However, optimizing certain parameters can significantly improve the study efficiency and reliability of the final results. Some reviews and book chapters have already provided the basic fMRI experimental design concepts (Amaro and Barker, 2006; Friston et al., 2007; Filippi, 2009; Bennett and Miller, 2013; Maus and van Breukelen, 2013). The experimental designs used in fMRI are resting state and task-based.

### Resting state

Characterization of the resting state is the most straightforward experimental design in fMRI. The subjects are not performing any explicit task (Figure 1B). During acquisitions performed under these circumstances, consistent and stable functional patterns, which are reproducible across individuals, sessions, scanners, and methods can be identified and are known as Resting State Networks (RSNs) (Fox et al., 2005; Damoiseaux et al., 2006; Long et al., 2008; Choe et al., 2015; Jovicich et al., 2016). That said, the specific resting conditions and the duration of the acquisition both have an important effect on the final functional signals. The most traditional design consists of instructing the participants to keep their eyes closed, not to think about anything in particular and not falling asleep. Alternative approaches have included keeping the eyes open or keeping the eyes open while fixating upon an object in the visual field, such as a cross, during scanning. The most suitable approach depends on the research question and purpose. If reliability and consistency are of upmost importance, the eyes fixated condition should be preferred, except for the primary visual network whose connectivity is more reliable with the eyes open but not fixated condition (Yan et al., 2009; Patriat et al., 2013; Zou et al., 2015). On the other hand, if the focus is on obtaining higher functional connectivity (FC) strength, eyes open, either fixated or not, should be used (Yan et al., 2009; Van Dijk et al., 2010). The chosen approach can also have a significant impact on the topological organization (Xu et al., 2014), global signal amplitude (Wang X.-H. et al., 2015; Wong et al., 2016), and directionality (Zhang et al., 2015). Nevertheless, the different resting-state conditions present comparable results, and thus the choice of the condition should also take into account which is more comfortable/appropriate for the study population, keeping in mind that it should be consistent for all the study participants. Differences in scan length also have a demonstrable impact, with acquisition times of 5–7 min shown to yield a reasonable trade-off between time/robustness of RSNs FC (Van Dijk et al., 2010; Whitlow et al., 2011), 5.5 min shown to be acceptable in young children (White et al., 2014), but both increased reliability and greater in-depth analysis are possible with scans of ~13 min (Birn et al., 2013).

### Task-based

When employing task-based fMRI studies, the way in which the stimuli are presented as a function of time is of upmost importance. The typical experimental designs are termed block (Figure 1C), event-related (Figure 1D), and mixed block/event-related (Figure 1E). The most simple task design, block-design, consists of presenting consecutive stimuli as a series of epochs, or blocks, with stimuli from one condition being presented during each epoch, followed by an epoch of stimuli from another condition, or with rest/baseline epochs. Specific block duration depends on the type of stimulus, with 15–30 s the most commonly used range, although some researchers suggest an optimal length of 15 s (Maus and van Breukelen, 2013). The order of the conditions is also important, and these are recommended to be counter-balanced across subjects of the same study. Block design allows a straightforward approach, good statistical power, signal amplitude and robustness. However, because each block is of such long duration, the participant's rapid habituation to task as well as the inability to accurately define response-time courses are intrinsic limitations of this design (Dale and Buckner, 1997; Amaro and Barker, 2006; Dosenbach et al., 2006).

Event-related designs are intended to delineate the association between brain functions and discrete events (typically randomized and of short duration between 0.5 and 8 s), separated by an inter-stimulus interval (ISI, normally ranging from 0.5 to 20 s). By incorporating great task flexibility and participant's unpredictability, this design provides the means to detect transient variations in local hemodynamic response. It presents however a more complex analysis process and a decreased signal-to-noise ratio (SNR), the combination of which leads to diminished detection power (Dale, 1999; Miezin et al., 2000; Huettel, 2012; Liu, 2012). Two types of event-related designs can be implemented and are characterized by different ranges of ISI: slow event-related designs, where the individual stimuli are well-separated in time (usually by more than 15 s), which prevents the overlap of successive stimuli HRFs, and rapid event-related designs, where stimuli are closely spaced in time (less than the HRF of the previous stimulus) resulting in the overlap of their HRFs. The latter protocols allow higher stimulus frequencies, resulting in greater statistical power, as well as diminished participant anticipation and boredom (Amaro and Barker, 2006; Huettel, 2012). Additionally, the randomized or pseudo-randomized order of stimuli presentation also is of importance in minimizing habituation. For these rapid event-related designs, implementing variable ISIs (jittering) allows differential overlap of HRFs, reduces multicollinearity problems and may provide better characterization of each condition response (Dale, 1999). Alternative methods as m-sequences (Buracas and Boynton, 2002; Liu, 2004) and genetic algorithms (Wager and Nichols, 2003; Maus et al., 2010) also can be used in event-related experimental designs, in order to reach flexible trade-offs between estimation efficiency and detection power. Some tools which can facilitate the implementation of randomized design are Optseq2 (https://surfer.nmr.mgh.harvard.edu/optseq/), RSFGen (http://homepage.usask.ca/~ges125/fMRI/RSFgen.html) and the fMRI Simulator (http://www.mccauslandcenter.sc.edu/crnl/tools/fmristim).

Combining stimuli in discrete blocks (mixed block/event-related design) provides information about both sustained and transient functional activations during task performance. While the technique offers the advantages of both block and event-related designs, it involves more assumptions, has a poorer HRF estimation and decreased statistical strength of sustained signal, and requires more subjects in order to measure statistically significant and sustained effects (Visscher et al., 2003; Amaro and Barker, 2006; Petersen and Dubis, 2012).

Independent of the experimental design, the specific way with which the study conditions are modeled (model specification) also plays an important role in the signal optimization process (Price et al., 1997; Friston, 2005; Amaro and Barker, 2006; Friston et al., 2007). The most basic comparison consists of subtracting two or more conditions (e.g., A − B), in which one is typically a control condition. Factorial designs expand this principle to two or more factors (e.g., different cognitive processes), each one with two or more levels. A simple example of such design would be the visualization of two different words in two different colors which would result in 4 conditions: the first word with the first color (A) the first word with the second color (B), the second word with the first color (C) and the second word with the second color (D). This design, not only enables the exploration of the effect of the two main factors (words and colors), but also their interactions, specifically how one factor affects the relation between the other factor and the response variables [e.g., (A − B) − (C − D)]. If the researcher is interested in assessing if the BOLD response to trials is modulated by a continuously varying parameter, a parametric design (e.g., A < A < A < A) would be more suitable. A typical example would be a study where the goal is to assess if the BOLD response increases/decreases linearly with the difficulty of the task. Choosing appropriate baselines and controls is of paramount importance since neural activity may vary unpredictably and overlap (or even exceed in amplitude) regions activated during the target task. A properly defined baseline should allow for maximum sensitivity in the detection of brain activity related to the study target (target isolation) while controlling for as many extraneous variables and unrelated confounds as possible (Stark and Squire, 2001; Peck et al., 2004; Diers et al., 2014). Generic recommendations include the use of multiple baseline conditions, scan times as long as possible (the more trials the better, with several shorter runs preferred over one long run), randomized conditions when possible, avoidance of comparison between trials widely separated in time and keeping participants engaged (Friston et al., 2007).

Several software tools can be used to implement the stated principles and present the task to the participants in the scanner (Table 2).

When designing a study involving both task-based and rs-fMRI, in order to avoid contamination of rs-fMRI with residual activity from previous task performance, it is recommended that one perform the resting state acquisition before the task-based or, at the minimum, after a suitable delay (Stevens et al., 2010; Tung et al., 2013).

### Power analyses

The question about “how large is enough” is a matter of debate in the neuroimaging field to determine the appropriate study sample size. For example, in an attempt to establish the boundaries for an adequate sample size, sensitivity and sensibility analyses were conducted, demonstrating that sample sizes of at least 27 subjects provide adequate reliability for fMRI investigations (Thirion et al., 2007). Additionally, in a controversial technical note (Friston, 2012), it was suggested that there is an optimal sample size, compared to which sample sizes could be either too small (studies with less than 16 subjects) or even, although less frequently, too large (studies with more than 32 subjects), under the arguments of reduced power or meaningless/trivial findings resulting from overpowered studies, respectively. It was mentioned that: on one hand, significant findings obtained in small samples (*n* = 16) indicate large effects being stronger than the same level of significance obtained with larger sample sizes; on the other hand, the relevance of significant findings obtained with large samples can be illustrated with the magnitude of observed effect-sizes. However, criticisms have been outlined (e.g., Yarkoni, 2012), particularly focusing on the liberal assumptions (e.g., significance threshold) in which Friston's arguments were built. Furthermore, it was recently described that a substantial number of published studies are statistically under-powered (Button et al., 2013). In this context, it is important to highlight the use of power analyses as a means to obtain robust and meaningful findings in these studies. Power analyses refer to the probability of rejecting the null hypothesis (given that the alternative hypothesis is true) and allow the establishment of a sample size that will increase the confidence of detecting true effects (Ioannidis, 2008). Functional MRI studies are often characterized by low statistical power, primarily due to limited sample size and large number of comparisons (Murphy and Garavan, 2004). Calculations of power are rarely performed in fMRI research, possibly due to the uncertainty associated to the unknown variance of the BOLD response and also due to the difficulty in predicting expected effects (Guo et al., 2012). Software tools have been developed in order to facilitate calculation of the statistical power, both for estimating the number of subjects to be included in the study, and for the number of stimuli to be presented. In order to employ these tools, information about the mean activation, the variance, the Type I error rate, and the sample size must be provided (Mumford, 2012). The power calculation should use either the statistical images (t/F maps generated by simple study designs) from pilot studies (PowerMap software; Joyce and Hayasaka, 2012), the estimated parameters in specific regions-of-interest (fMRIPower tool) (Mumford and Nichols, 2008) or the prevalence of active peaks (NeuroPower; Durnez et al., 2016).

## Data acquisition techniques and artifacts

Performing effective fMRI studies requires a thorough understanding of specific MRI acquisition techniques and artifacts, and how to deal with them (Figures 1F,G). When the activity of a population of neurons within a voxel (minimum spatial resolution unit in each image, the volume element) changes, the associated hemodynamic response can be determined using T2^*^ weighted MRI acquisitions (details in Buxton, 2009; Hashemi et al., 2012). Detection of the BOLD signal is the most commonly used technique in fMRI, due primarily to its ease of implementation and inherent functional contrast. Alternative detection methods do exist and are based on the measurement of a combination of additional parameters including: changes in cerebral blood volume (CBV), cerebral blood flow (CBF), and cerebral metabolic rate of oxygen (CMRO2) (Davis et al., 1998). The alternative methods are: calibrated BOLD, based on BOLD contrast but also taking into account physiological variation (e.g., heamatocrit, oxygen extraction fraction, and blood volume) (Davis et al., 1998; Blockley et al., 2012); Arterial Spin Labelling (ASL) used to measure regional CBF by tracking intravascular water as an endogenous tracer (Williams et al., 1992; Buxton et al., 1998; Telischak et al., 2015); Vascular-Space-Occupancy (VASO) based on differences between blood and surrounding tissues and determined through dynamic measurement of local CBV (Lu et al., 2003; Lu and van Zijl, 2012); Venous Refocusing for Volume Estimation (VERVE), based on changes in venous cerebral blood volume (Stefanovic and Pike, 2005); Signal Enhancement by Extravascular Protons (SEEP) based on the determination of proton-density changes associated with cellular swelling (Stroman et al., 2003; Figley et al., 2010); and diffusion-weighted fMRI, which measures structural changes in the neural tissues related to cell swelling during activation (Le Bihan, 2012; Aso et al., 2013).

Functional MRI data are generally collected over the entire brain through the acquisition of sequential volumes (time-points), each one composed of a set of slices. The typical sequence used for fMRI studies is echo planar imaging (EPI), which is attractive due both to its imaging speed and BOLD contrast sensitivity, but also associated with inherent artifacts and diminished image quality (Stehling et al., 1991; Poustchi-Amin et al., 2001; Schmitt et al., 2012). EPI may be performed using gradient-echo, spin-echo, or combination techniques. When compared to spin-echo EPI, gradient echo acquisitions have higher BOLD sensitivity, imaging speed and versatility, and have been used in the majority of fMRI studies. On the other hand, spin-echo sequences have been proposed as a viable alternative when the goal is to obtain increased functional localization in the capillary bed (especially at high fields) and when specific regions of interest (ROIs) are less superficial regions such as for example the ventromedial frontal and anterior inferior temporal cortex are the primary focus of the study (Norris, 2012; Boyacioğlu et al., 2014; Halai et al., 2014; Chiacchiaretta and Ferretti, 2015).

### Data acquisition techniques

In order to minimize artifact, and to obtain the most reliable data it is critically important to optimize the acquisition phase. There is no single “gold standard” fMRI protocol due to the great variability in parameters such as the MRI hardware vendor and configuration, field strength, scanning time available, specific regions under study and subsequent analyses intended. For this reason, we here confine ourselves to a series of suggestions based upon the use of a standard single-shot gradient-echo EPI 3 T fMRI acquisition. When defining an fMRI acquisition protocol, a reasonable strategy is to start from a well-characterized “standard” protocol usually provided by the vendor, and then to modify it according to the specific requirements of the study to be undertaken. A practical description of the parameters involved in a typical fMRI acquisition, and guide to how they should be reported, is provided in Inglis' checklist (Inglis, 2015). While many characteristics of the individual MRI scanner and of the specific acquisition protocols have a strong impact on the fMRI results, magnetic field strength is amongst the most defining. The amplitude of signal usually associated with the BOLD contrast is very low (around 1% of baseline or less). With increased field strengths the sensitivity is increased as is the spatial resolution and SNR (Gore, 2003; van der Zwaag et al.,, 2009; Wald, 2012; Skouras et al., 2014), but all at the cost of increased artifact (Triantafyllou et al., 2005). The majority of scanners currently in use, both in diagnostic and research centers, are units having field strengths of 1.5–3 T, but some research groups are already utilizing 7 T fields, and it is expected that the availability and use of such scanners will increase (Duyn, 2012). Typically, fMRI data are acquired using a series of 2D axial slices to cover the whole brain (one volume) and then the process is repeated to collect a number of volumes over time (time-series). Each volume can be acquired using either interleaved or sequential slice acquisitions. While interleaved acquisitions have less adjacent slice interference, they can be more vulnerable to spin history effects generated by head motion (Muresan et al., 2005). To reduce the influence of both these potential issues, most fMRI acquisitions utilize a gap between slices (around 10–25% of the total slice thickness). Slice acquisition also can be performed either in an ascending (foot-to-head) or descending order, with the former theoretically affected by excitation and saturation of in-flowing blood. Although no significant differences have been reported between the two directions, the most robust approach seems to favor the use of descending sequential acquisitions (Howseman et al., 1999).

An important trade-off in fMRI acquisition is between temporal and spatial resolution. Since the BOLD signal changes as a function of time, optimizing the temporal resolution is critical. Typical fMRI acquisitions with full brain coverage have repetition times (TRs) of 2–3 s (the time it takes to acquire one volume). For task-based studies, shorter TRs are usually chosen for event-related designs than for block designs, due to the relative lack of experimental power and greater importance of time-course information. Shorter TRs may lead to a significant reduction in SNR while longer TRs are theoretically associated with higher sensitivity to motion (Filippi, 2009; Wald, 2012; Craddock et al., 2013). Due to the necessity of optimizing temporal measurements, spatial resolution is usually sacrificed. With high-field strengths and/or if full brain coverage is not mandatory for the specific study, the TR can be made as low as 1 s, or even less. One way of increasing temporal resolution while still maintaining full brain coverage is to use a parallel imaging method, such as GRAPPA (Griswold et al., 2002), SENSE (Pruessmann et al., 1999), or multiplex-EPI (Feinberg et al., 2010). GRAPPA and SENSE work by reducing the time required for acquiring a single slice but increasing the sensitivity to motion. Thus, extra care should be taken, especially with participants prone to move a lot during scanning. On the other hand, multiplexed-EPI works by simultaneously acquiring more than one slice at a time (Feinberg et al., 2002). However, the simultaneous excitation of slices causes signal leaking from one slice to the other, which increases with the number of slices acquired simultaneously (i.e., the acceleration factor) and also induces artifactual thermal noise correlations, critical for functional connectivity studies (Setsompop et al., 2013). The combination of both techniques can also be employed, further reducing the acquisition time and with revealed increased sensitivity to detect RSNs at moderate acceleration factors (Preibisch et al., 2015). Isotropic voxels are recommended (in-plane resolution and slice thickness with equal dimensions) because the folded cortex has no dominant orientation. At 3 T fields, typical voxel sizes range between 2.8 and 3.5 mm^3^ (Wald, 2012; Craddock et al., 2013). Higher spatial resolution can be achieved at higher field strengths, but is associated with increased artifact (Olman and Yacoub, 2011). A square Field of View (FOV) ranging between 192 and 224 mm, with a matrix size of 64 and slice number of 30–36, is common at 3T. The most critical parameter when optimizing an fMRI protocol with respect to timing is the interval between slice excitation and signal acquisition, known as echo time (TE). The interval choice of TE in order to maximize the BOLD contrast depends on the tissue characteristics and the field strength and is ideally equal to the apparent tissue T2^*^. The TE for 3 T field strength is typically around 30 ms (ranging from 25 to 40 ms) (Gorno-Tempini et al., 2002; Craddock et al., 2013; Murphy et al., 2013). The appropriate flip angle also is of relevance when optimizing the BOLD signal. One recommended practice is to select a flip angle equal to the Ernst angle (Ernst and Anderson, 1966) for gray matter. More recently, however, it has been shown that the use of much lower flip angles is possible, as long as physiological noise is the dominant noise source in fMRI time-series (Gonzalez-Castillo et al., 2011). For field strength of 1.5 T and a TR of 3 s, the Ernst angle is ~89°, resulting in the common choice of 90° for flip angle. For 3 T and a TR of 2 s, the angle is closer to 77° (Ernst and Anderson, 1966). These specifications are even more complex when a multicenter study is planned, and a number of considerations need to be taken into account in order to maximize reproducibility (Stöcker et al., 2005; Friedman and Glover, 2006; Glover et al., 2012; Keator et al., 2016).

Some important tips include: for studies involving both resting state and task-based fMRI, it is recommended that the same acquisition protocol be used, or at least, as similar as possible, in order to most accurately integrate and compare results (Ganger et al., 2015; Pernet et al., 2016); when performing task-based studies, it is of upmost importance to precisely synchronize scan acquisition with stimulus presentation. Such synchronization can be achieved through the use of manual configurations (e.g., sending triggers between the scanner and stimulus presentation software) or with integrated solutions such as the Lumina Controller (http://cedrus.com/lumina/controller/), SyncBox (http://www.nordicneurolab.com/products/SyncBox.html), SensaVue fMRI (http://www.invivocorp.com/solutions/neurological-solutions/sensavue/), or nordic fMRI solution (http://www.nordicneurolab.com/products/fMRISolution.html).

### Artifacts

The primary goal of any fMRI acquisition is to obtain the highest possible SNR and contrast-to-noise ratio (CNR) (Welvaert and Rosseel, 2013) while minimizing the impact of artifacts. The artifacts in fMRI are usually related to the pulse sequence, gradient system hardware, acquisition strategy used as well as physiological noise. Three artifacts are characteristic of the traditional EPI pulse sequence: spatial distortions (Figure 1G1), signal dropouts (Figure 1G2), and ghosting (Figure 1G3). Geometric and intensity spatial distortions may result from static field inhomogeneity and appear locally either as stretched or compressed pixels along the phase-encoding axis, being worse at higher field strengths. A number of strategies have been suggested to correct the distortions, and include the use of shimming coils (Reese et al., 1995; Balteau et al., 2010), field mapping (Hutton et al., 2002; Zeng and Constable, 2002), point spread function mapping, or reversed phase gradients (Holland et al., 2010; In et al., 2015). Signal dropouts due to field inhomogeneities near air/tissue interfaces, particularly prevalent in the frontal and temporal lobes, also occur in EPI. The choice of an appropriate echo time (TE, described below; optimum BOLD contrast occurs when the TE matches the local T2^*^ of the tissue of interest), greater number of thinner (rather than lower number of thicker) slices, as well as optimizing slice tilt, the direction of the phase-encoding or the z-shim moment may all help to reduce these dropouts (Weiskopf et al., 2006; Balteau et al., 2010). Ghosting artifacts, which occur only in the phase-encoding direction, are triggered because odd and even lines of k-space are acquired with opposite polarity. Techniques such as implementing a multi-echo reference scan, two-dimensional phase correction or applying dual-polarity generalized autocalibrating partially parallel acquisitions (GRAPPA), can reduce the magnitude of these effects (Schmithorst et al., 2001; Chen and Wyrwicz, 2004; Robinson et al., 2013; Hoge and Polimeni, 2015). Hardware-related artifacts such as scanner and head coil heterogeneities, spiking, chemical shifts, and radiofrequency (RF) interferences all can significantly impact the fMRI image quality and compromise results (Bernstein et al., 2006; Poldrack et al., 2011). One approach for reducing the impact of these artifacts is to implement an Independent Component Analysis (ICA) or Robust Principle Component Analysis (RPCA) (Behzadi et al., 2007; Griffanti et al., 2014; Campbell-Washburn et al., 2016). Although hardware-related artifacts can, at least theoretically be fixed, participant related confounds will always be present. Participant's physiological confounds such as head motion (Power et al., 2012), cardiac, and respiratory “noise” as well as vascular effects all have a significant impact on the final fMRI results (Faro and Mohamed, 2010; Murphy et al., 2013). The most common and critical artifact in fMRI is head motion. Even though it is common to correct for subject motion during preprocessing (see preprocessing section), the best approach is to prevent motion as much as possible in the first place using comfortable padding and optimized head fixation (Edward et al., 2000; Heim et al., 2006), as well as to fully inform the subject in advance about scanner noise and the confining environment. Performing multi-echo acquisitions can also help reduce motion artifacts (Kundu et al., 2013). Cardiac pulsation and the respiratory cycle can have an impact similar to that of head motion. Due to the long repetition time (TR, see below) of standard BOLD EPI acquisitions (2–3 s) the fluctuations are aliased into low-frequency signals which may be mistaken for neural activity-related BOLD oscillations, especially on rs-fMRI (Birn, 2012; Murphy et al., 2013; Cordes et al., 2014). A number of strategies have been used in an attempt to reduce these artifacts, and include the use of band-stop filtering, dynamic retrospective filtering (Särkkä et al., 2012), image-based methods (RETROICOR; Glover et al., 2000), corrections based on canonical correlation analysis (Churchill et al., 2012c) and through the use of externally recorded cardiac and respiratory waveforms as regressors (Falahpour et al., 2013).

Thorough understanding of the link between neural activity and the hemodynamic changes that give rise to the BOLD signal (neurovascular coupling), as well as the variation in its response, should help to reduce the inter-subject variability and increase the homogeneity and statistical power of the studies (D'Esposito et al., 2003; Handwerker et al., 2012; Liu, 2013; Phillips et al., 2015). One key feature is that as the signal increases (field strength, array coils), the physiological noise increases proportionally (Triantafyllou et al., 2005, 2006; Hutton et al., 2011). A great variety of software tools have been developed to minimize the impact of artifacts, for example the Artifact detection Tool (ART—http://www.nitrc.org/projects/artifact_detect/), the Physiological Artifact Removal Tool (PART—http://www.mccauslandcenter.sc.edu/CRNL/tools/part), the PhysIO Toolbox (http://www.translationalneuromodeling.org/tnu-checkphysretroicor-toolbox/), the ArtRepair Software (http://cibsr.stanford.edu/tools/human-brain-project/artrepair-software.html), the FMRIB's-based Xnoisifier (FIX) (http://fsl.fmrib.ox.ac.uk/fsl/fslwiki/FIX), and the RobustWLS Toolbox (http://www.icn.ucl.ac.uk/motorcontrol/imaging/robustWLS.html) (Diedrichsen and Shadmehr, 2005). While a significant problem in task-based fMRI, artifact identification and removal is even more complex with rs-fMRI. In the absence of an *a priori* hypothesis, it may be hard to distinguish the signal related to neural activity from the sources of noise, particularly when the artifacts are spatially or temporally correlated and may share a degree of spatial or spectral overlap with the RSNs. Whenever artifacts cannot be corrected, it may be necessary to adopt some alternative strategies such as the exclusion of the affected subject, volume or slice, or to limit the analysis to regions without significant artifacts.

## Quality control and preprocessing

Quality control and preprocessing procedures are key steps in the detection and correction of artifacts in fMRI, thus providing consistency and reliability to maps of functional activation. A variety of automated preprocessing pipelines have been described and implemented [e.g., DPABI, LONI (Rex et al., 2003), Nipype (Gorgolewski et al., 2011), BrainCAT (Marques et al., 2013) and C-PAC], but there is a lack of consensus about which workflow is the most effective. Several studies and reviews have explored the effects of preprocessing techniques on both task-based (Strother, 2006; Churchill et al., 2012a,b) and rs-fMRI results (Aurich et al., 2015; Magalhães et al., 2015). Herein we attempt to provide a practical guide to the most commonly used methodologies.

### Acquisition quality control and data conversion

The first quality control point comes during the acquisition phase. It is important to loop through the images using real-time display of the scanner, while it is still possible to repeat the acquisition and not lose data. Assessing the images using two different contrast settings, standard anatomical (to verify the appearance of the brain, gross head motion and spiking) and background noise contrast (to verify hardware issues and important small motion) is a wise strategy (Figure 1H). Following data acquisition, it is important to verify that all images have been imported and sorted correctly, and to ensure the same acquisition protocol has been used for all study participants. At this point, inspection of the scans to screen for obvious brain lesions (except for those specifically being studied) as well as visible artifacts can be performed using general-purpose viewers, such as Osirix, MRIcro, RadiAnt, or ImageJ (Escott and Rubinstein, 2003; Rosset et al., 2004). Due to the absence of a standard file format, it is necessary to start by converting the original scanner data from DICOM format (Mildenberger et al., 2002; Liao et al., 2008; Mustra et al., 2008) to the most common file format used by fMRI preprocessing tools, the NIfTI format (Neuroimaging Informatics Technology Initiative, allows both separated ^*^.img and ^*^.hdr files or both combined on a single ^*^.nii file) (Poldrack et al., 2011), which is an extension from the Analyze 7.5 format (set of two files: ^*^.img containing the binary image data and ^*^.hdr with the metadata) (Figure 1I). In the NIfTI format most of the DICOM header information is discarded (e.g., patient information) and only basic acquisition information (e.g., TR, resolution, FoV, image orientation) is kept. Most of the fMRI processing packages include file converting tools, and several dedicated converters also are available [e.g., dcm2nii (https://www.nitrc.org/plugins/mwiki/index.php/dcm2nii:MainPage), MRIConvert (https://lcni.uoregon.edu/downloads/mriconvert/mriconvert-and-mcverter) and NiBabel (http://nipy.org/nibabel/index.html)].

### Initial stabilization, slice-timing, and motion correction

Upon beginning an acquisition, the scanner typically takes some seconds to completely stabilize its gradients, and the tissue being imaged requires some time to reach the necessary excitation. To remove the influence of these factors, it is common to discard the initial volumes (usually around the 10 initial seconds) of the fMRI acquisitions whether for task-based or rs-fMRI. Because fMRI volumes are acquired as 2D images, one slice at a time, and even though short and fixed TRs are utilized, there is an intrinsic delay between the real and the expected slice acquisition times, which may substantially decrease the ability to discern a given effect. The interval between the first and the last acquisition slice depends on the TR selected. Slice timing correction adjusts the time-course of voxel data in each slice to account for these differences by interpolating the information in each slice to match the timing of a reference slice (first or mean TR slice) (Calhoun et al., 2000; Sladky et al., 2011) (Figure 1J). The impact of using slice-time correction is described as quite variable, depending on the type of study, ranging from very important for event-related designs (especially for time-course analysis), to less important for block designs, to having minimal effect on rs-fMRI. However, it seems that it never has a negative impact on the results (Henson et al., 1999; Sladky et al., 2011; Wu et al., 2011). In addition to the debate about whether or not to employ slice timing correction is the issue of, if used, when such correction ought be done, as this step can interact strongly with motion correction (described below). Common suggestions include: for interleaved acquisitions, it is usually performed before motion correction and for sequential acquisitions thereafter; for subjects with low head motion performed before motion correction and with high head motion after (it is recommended to keep the order consistent for all the study subjects) (Sladky et al., 2011). Nevertheless, the issue remains poorly addressed as slice timing and motion correction are two inextricably linked steps (Bannister et al., 2007). An alternative option is to perform slice timing and motion simultaneously through 4d realignments using the Nypipe 4d realignment function (Roche, 2011) or with the Seshamani data reconstruction framework (Seshamani et al., 2016). Additional methods exist for slice timing adjustment, such as adding regressors as nuisance variables (Henson et al., 1999) or altering the model rather than the data, as in dynamic causal modeling (DCM) (Kiebel et al., 2007), though that specific approach is not suitable for interleaved acquisitions.

Head motion during scanning is probably the most common and critical confound for both task and rs-fMRI studies, both of which are dependent upon precise spatial correspondence between voxels and anatomical areas over time (Friston et al., 1996; Satterthwaite et al., 2012; Maclaren et al., 2013; Zeng et al., 2014; Power et al., 2015). The most common strategy used to perform motion correction is first to realign each volume to a reference volume (mean image, first, or last volume) using a rigid body transformation (x, y, and z rotations and translations) (Jiang et al., 1995) (Figure 1K). While there is no standard rule about the motion threshold to be used, it is a rule of thumb to discard data sets with motion greater than the dimensions of a single voxel (Formisano et al., 2005; Johnstone et al., 2006). Because most traditional realignment strategies take into account each volume at a single point in time, and due to the fact that residual motion-induced fluctuations still are present in the data set and decrease the reliability and statistical sensitivity of the study, a different strategy was proposed. This technique was to include in the subject-level general linear model (GLM) the motion parameters estimated during the realignment step as “nuisance variables” (covariates of no interest), possibly also including the temporal derivatives of those variables (Friston et al., 1996; Johnstone et al., 2006; Power et al., 2012). Most of the commonly used fMRI packages include motion correction tools, and significant differences in their performance are not evident (Oakes et al., 2005; Morgan et al., 2007). Several groups have recently demonstrated that small head motion produces spurious but structured noise, which then triggers distance-dependent changes in signal correlations (Power et al., 2012, 2015; Satterthwaite et al., 2012; Van Dijk et al., 2012; Siegel et al., 2014). The method proposed to reduce these effects has been called Scrubbing, and is based on two measures to capture the head displacements, Framewise Displacement (FD) or the brain-wide BOLD signal displacements (temporal Derivative VARiance—DVARS) derived from volume to volume measurements over all brain voxels (Power et al., 2012). After FD or DVARS calculation, a threshold is applied and, despite a lack of standardization, it is common to use FD > 0.2–1 mm and DVARS > 0.3–0.5% of BOLD signal in order to identify outliers. Scrubbing corrections can be implemented with several tools including the C-PAC, Artifact Detection Tools (Mazaika et al., 2007), DPARSF (Yan and Zang, 2010) and fsl_motion_outliers tool. By default, fsl_motion_outliers detects outliers if FD or DVARS exceeds the 75th percentile + 1.5 times the InterQuartile Range. The identified outliers are commonly regressed out later in the preprocessing pipeline (but before temporal filtering) from the data with a GLM where each outlier is entered as a nuisance regressor. Additional alternative motion correction strategies are available, such as the use of slice derived information (Beall and Lowe, 2014), task associated motion (Artifact Detection Tool), expansion to 24–36 motion regressors (Power et al., 2014), independent component analysis de-noising (Mowinckel et al., 2012; Griffanti et al., 2014; Pruim et al., 2015), and group-level motion covariates (Van Dijk et al., 2012). Furthermore, the use of non-gray matter nuisance signals (Behzadi et al., 2007; Jo et al., 2013) and regression of global signal (Power et al., 2014) have been shown to help reducing the impact of motion.

### Spatial transformations

Performing spatial transformations to align the images from the individual's native space with those acquired from a different modality or subject [(co-)registration] or into a common standard space (normalization) is a fundamental step of the fMRI preprocessing (Brett et al., 2002) (Figure 1L). If homologous brain regions are not properly aligned between individuals, sensitivity is lost and leads to an increase in the false negatives rate. On the other hand, systematic normalization errors between groups may trigger false positive activations. In fMRI studies there are two main standard coordinate systems which have been used in order to reduce intersubject variability and to facilitate the reporting of results in the form of standard stereotactic (x,y,z) coordinates. These are the Talairach space, where the principal axis corresponds to the anterior commissure-posterior commissure (AC-PC) line, and which is based upon the brain of a single individual (Talairach and Tournoux, 1988), and the Montreal Neurological Institute (MNI) templates (there are several MNI templates available, being the MNI152 the most commonly used), which are based on the average of T1-weighted MRI scans of a large number of subjects (Mazziotta et al., 1995, 2001). These templates normally are associated with an atlas (Cabezas et al., 2011; Evans et al., 2012) and allow the localization of designated anatomical features in coordinate space, as well as the association of functional results to identified anatomical regions. The Automated Anatomic Labelling (AAL) (Tzourio-Mazoyer et al., 2002), the Talairach atlas (Lancaster et al., 2000), and the Harvard-Oxford atlas (Desikan et al., 2006) are amongst the most commonly used. It is important to note that the Talairach and MNI coordinates do not refer to the same brain regions or structures (Laird et al., 2010), and it is frequently necessary to convert between the two (e.g., for meta-analyses). Available tools to implement transformation between the two coordinate spaces include the “icbm2tal” (Lancaster et al., 2007; Laird et al., 2010) (GingerALE, http://www.brainmap.org/icbm2tal/) and the “mni2tal” (Brett et al., 2002) (BioImage Suite, http://bioimagesuite.yale.edu/mni2tal/). Tools also are available to localize and label brain regions according to the MNI (MRIcron, http://www.mccauslandcenter.sc.edu/mricro/mricron/, Neurosynth, http://neurosynth.org/) or Talairach (Talairach software, http://www.talairach.org/, WFU_PickAtlas, http://www.nitrc.org/projects/wfu_pickatlas/) coordinates. Normalization strategies rely on optimization functions which maximize the similarity between two images (Jenkinson and Smith, 2001) by applying translations, rotations, and scaling in multiple axes. Transformations are usually divided into two subtypes: linear, applied uniformly along an axis and usually represented as affine matrices, and non-linear, defined locally (meaning that different points along an axis undergo unique transformations) usually defined by warp or distortion maps. Several deformation algorithms are available which can be applied to MRI registrations (Klein et al., 2009). An alternative registration method is the use of surface registration techniques, in which the functional time series are mapped onto cortical surface models [e.g., automatically implemented by Freesurfer (Fischl et al., 1999)], improving the computational efficiency and the mapping of the cortical surface, beneficial for subsequent processing and analysis steps (surface-based smoothing kernels and surface-registration can be used) (Klein et al., 2010; Khan et al., 2011).

In fMRI there are two commonly used processing streams for spatial normalization. In one, a single step strategy is used to normalize directly to a standard EPI template, while the other employs a multi-step method which first aligns to the matching structural image using rigid-body or affine transformations, following which the composite image is then registered to the reference space, using either affine or non-linear transformations (Poldrack et al., 2011). Complementary techniques for removing non-brain areas from the analysis and reducing the data size, such as skull striping or masking, may also help to improve the normalization step (Tsang et al., 2007; Andersen et al., 2010; Fischmeister et al., 2013). The choice of the optimal atlas template and mapping function depends on a multitude of factors and is influenced by age, gender, hemispheric asymmetry, normalization methodology, and disease-specificity (Crinion et al., 2007). Following the normalization step, it is always important to perform visual quality control, for example by displaying the fMRI data of each participant along with a reference EPI template.

### Spatial smoothing and filtering

The next preprocessing step normally implemented is that of spatial smoothing/filtering, a process during which data points are averaged with their neighbors, suppressing high frequency signal while enhancing low frequency ones, and results in the blurring of sharp edges (Figure 1M). Smoothing simultaneously increases the SNR and the validity of the statistical tests (from random field theory) by providing a better fit to expected assumptions while reducing the anatomical differences. On the other hand, smoothing reduces the effective spatial resolution, may displace activation peaks (Reimold et al., 2006) and extinguish small but meaningful local activations depending on the filter parameters chosen (Yue et al., 2010; Poldrack et al., 2011; Sacchet and Knutson, 2013). The standard spatial smoothing procedure consists of convolving the fMRI signal with a Gaussian function of a specific width (as, spatially, the BOLD signal is expected to follow a Gaussian distribution). The choice of the proper size of the Gaussian kernel [Full Width at Half Maximum (FWHM)], which determines the extent to which the data is smoothed, will be dependent on specific features of the study undertaken, such as type of paradigm and inference expected, as well as on the primary image resolution. The amount of smoothing always should be the minimum necessary to achieve the intended results, and a reasonable starting point is a FWHM of twice the voxel dimension (care must be taken when using large smoothing kernels as they make the detection of smaller patterns of activation harder). The typical smoothing values used range between 5 and 10 mm for group analyses (Beckmann and Smith, 2004; Mikl et al., 2008; Poldrack et al., 2011). Alternative approaches to smoothing are the use of varying kernel widths (Worsley et al., 1996), adaptive smoothing (Yue et al., 2010; Bartés-Serrallonga et al., 2015), wavelet transforms (Van De Ville et al., 2007), and prolate spheroidal wave functions (Lindquist et al., 2006). Despite its common use, care must be taken when performing smoothing due to its effects on the final results (Geissler et al., 2005; Molloy et al., 2014), its interaction with motion correction (Scheinost et al., 2014) and impact upon analyses which are sensitive to the activation of individual voxels (such as ROI-to-ROI analysis, Regional Homogeneity and Multi-voxel Pattern Analysis). This step is not recommended for connectomic approaches in order to prevent the BOLD signal from extending across different regions of interest (Zuo et al., 2012; Tomasi et al., 2016).

A final step in the data preprocessing pipeline is temporal filtering (Figure 1N). This step is performed in order to remove the effects of confounding signals with known or expected frequencies. The use of frequency filtering (and/or spatial smoothing) may help attenuate noise and thus increase the SNR (White et al., 2001). Functional MRI time-courses often manifest low-frequency drifts which may reduce substantially the statistical power of the results. It is therefore of great relevance to attempt to identify which frequencies are those of interest and which are noise (Kruggel et al., 1999). For example, fMRI noise may be associated with slow scanner drifts (~ <0.01 Hz), as well as cardiac (~ 0.15 Hz) and respiratory (~ 0.3 Hz) effects (Cordes et al., 2001, 2014). The most frequently used filters for task-based fMRI acquisitions are high-pass filters (typically ~ 0.008–0.01 Hz, 100–128 s), generally deployed with a rough rule of using a cut-off value at least 2 times that of the fundamental task-frequency (the interval between one trial start and the next one). With rs-fMRI the standard strategy is to apply a band-pass filter (0.01–0.08 Hz) following the reports of Biswal and colleagues (among others), which have shown that spontaneous BOLD low frequency (~ <0.1 Hz) fluctuations were physiologically meaningful and reflect spontaneous neural activity (Biswal et al., 1995; Fransson, 2005; Shirer et al., 2015). Nevertheless, high frequency signals (>0.1 Hz) have also been shown to present functional significance (Chen and Glover, 2015; Gohel and Biswal, 2015). Exploring such frequency band requires extra caution in controlling for physiological sources of noise (e.g., respiratory and cardiac effects) as these are known to present frequencies greater than 0.1 Hz. This can be achieved using simultaneous monitoring of pulse oximetry, electrocardiogram and/or breathing belt.

Normally associated with the filtering step, detrending methods also are used to reduce the effects of noise (Tanabe et al., 2002; Friman et al., 2004). Complex spatio-temporal filters (Kriegeskorte et al., 2010), cross-validation (Ngan et al., 2000), adaptive filters (Deckers et al., 2006), matched-filter acquisitions (Kasper et al., 2014), bilateral filtering (Rydell et al., 2008), and multifiltering (Hui et al., 2013) are alternatives to the standard fMRI temporal filters.

Effective quality control is of fundamental importance in the optimization of data usability reliability and reproducibility. Software tools have been developed in order to implement quality control procedures complementary to the ones already mentioned, such as BIRN QA (http://www.nitrc.org/projects/bxh_xcede_tools/), NYU CBI Data Quality tool (http://cbi.nyu.edu/software/dataQuality.php), and the CANLAB Diagnostic Tools (http://wagerlab.colorado.edu/tools).

## Analysis methods

The next stage in the fMRI workflow is the selection of the most suitable method to extract the relevant functional information. There are many fMRI analysis methods and software tools for both task-based (Figure 1O) and rs-fMRI (Figure 1P). Thus, choosing the one most suitable for a specific study may be a complex, often confusing and time-consuming task. In order to assist with this choice, we herein present a table with the most commonly used software tools for the analysis of task-based and rs-fMRI data (Table 4). Some existing reviews have already explored fMRI analysis methods (van den Heuvel and Hulshoff Pol, 2010; Lohmann et al., 2013; Smith et al., 2013; Sporns, 2014; Haynes, 2015; Zhan and Yu, 2015; Pauli et al., 2016). In the following sections we distinguish between task-based and resting-state fMRI analysis according to the prominence use of each method, nevertheless, some are suitable for both fMRI acquisitions. Other distinctions could be performed, namely between methods suitable for localization and for connectivity approaches. The appropriateness application of each method will also be discussed below.

### Typical task-based analyses methods

**Figure 2:**
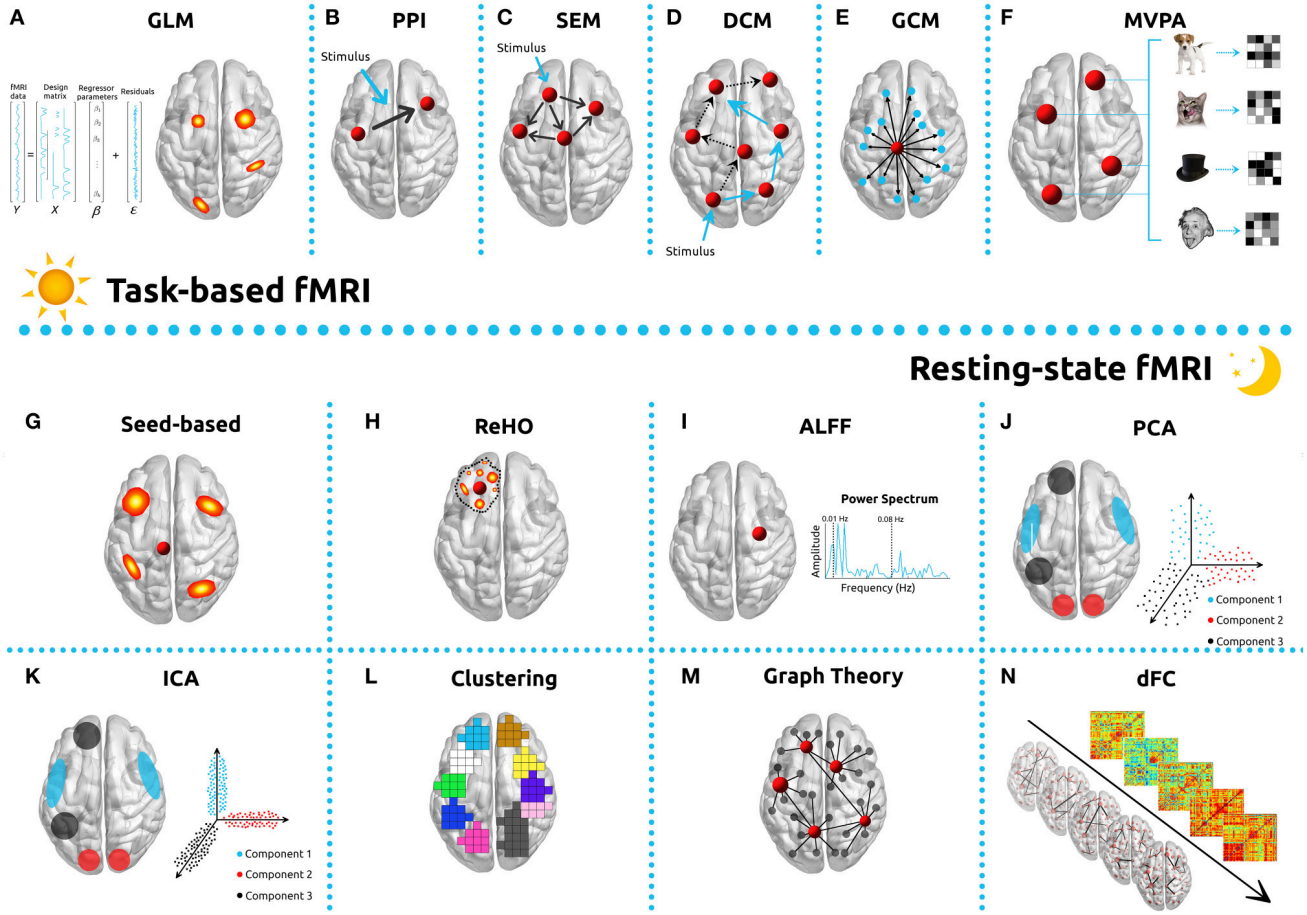
**Commonly used analysis methods in functional MRI studies**. For task-based analyses, implementing the General Linear Model (GLM, **A**), Psychophysiological interactions (PPI, **B**), Structural Equation Modeling (SEM, **C**), Dynamic Causal Modelling (DCM, **D**), Granger Causality Mapping (GCM, **E**), and Multi-voxel Pattern Analysis (MVPA, **F**) are common strategies. To analyze resting-state fMRI data, methods such as seed-based correlations **(G)**, Regional homogeneity (ReHo, **H**), Amplitude of Low Frequency Fluctuations (ALFF, **I**), Principal Component Analysis (PCA, **J**), Independent Component Analysis (ICA, **K**), Clustering **(L)**, Graph Theory **(M)**, or dynamic Functional Connectivity (dFC, **N**) can be implemented.

The most employed method in the analysis of task-based fMRI is Statistical Parametric Mapping (SPM), which is based on the GLM (Figure 2A) (Friston et al., 1994a; Kiebel and Holmes, 2003; Poline and Brett, 2012). GLM's popularity is based on its straightforward implementation, interpretability and computability. It incorporates most data modeling structures and provides the means for minimizing/controlling the effects of confounding factors such as motion, respiratory and cardiac and HRF derivatives (Calhoun et al., 2004; Lund et al., 2006; Bright and Murphy, 2015). One common approach in the use of this technique is to convolve the stimulus onsets and durations with a canonical HRF, which results in quantifying an estimate of the expected BOLD signal for any condition of interest. These estimates are then defined, along with intrinsic confounding factors (e.g., motion parameters), as the independent variables of the GLM. Each voxel time-series is then set as the dependent variable. The result of this process is to generate a test statistic for each voxel in the brain, which makes possible the creation of a parametric map (SPM). The process is performed separately for each subject and is commonly designated as first-level analysis. The GLM can be used very generally, ranging from the simplest subtraction method to parametric correlations with behavior, and also serves as the reference for several methods used to estimate connectivity. The main criticism of the GLM is based upon the intrinsic assumptions which must be made related to parametric testing in general, and the GLM in particular, and which are not usually verified nor are they tested (Monti, 2011). Despite these reservations, GLM analysis remains extremely popular for fMRI.

Task based connectivity analysis is being performed with increasing frequency and its results are quite sensitive to the choice of analysis tool. For it to be used appropriately, it is necessary to distinguish undirected associations between brain regions (functional connectivity—FC) from directed and causal relationships (effective connectivity) (Horwitz et al., 2005; Friston, 2011). Functional connectivity will be discussed in greater detail below, since the methods involved are more widely used for rs-fMRI, although some of the same principles apply also to task-based analysis. Also closely related to the GLM, and concerned with effective connectivity, psychophysiological interaction (PPI) is a method used to quantify how task-specific FC between a particular brain ROI (source/seed) and the rest of the brain voxels are affected by psychophysiological variables (Figure 2B) (Friston et al., 1997; O'Reilly et al., 2012). Some caveats when using PPI analyses are the hemodynamic deconvolution, the low power, and the difficulties intrinsic to event-related designs (Gitelman et al., 2003; O'Reilly et al., 2012).

Similar to PPI analysis in that it explores how the experimental context affects connectivity between a group of regions, the structural equation model (SEM) is used to assess the effective connectivity based on an *a priori* model of causality (Figure 2C) (McLntosh and Gonzalez-Lima, 1994; Büchel and Friston, 1997; Kline, 2011). It starts with the definition of a set of ROIs, and then tries to determine the connection strength between those ROIs that best fit the model. SEM allows the investigation of several brain regions simultaneously, and incorporates prior anatomical and functional knowledge to determine causal relationships, but assumes that the interactions are linear and, (similar to PPI) it cannot take into account the dynamic changes of the BOLD signal (Tomarken and Waller, 2005). Most often used for task-based fMRI, SEM has seen application with rs-fMRI (James et al., 2009).

DCM allows estimation of the effective connectivity (model states) between brain regions by determining hemodynamic response (model output) as a function of specified external experimental variables (model input) (Figure 2D). One of the primary characteristics of DCM is that it allows exploration of the brain as a dynamic system, accounting for changes in populations of neurons, and is able to build non-linear models of interacting regions (Friston et al., 2003; Penny et al., 2004; Stephan et al., 2008; Friston, 2009). DCM is a reliable and potentially a more biologically realistic method for fMRI in that it deals with function at the neuronal level. It does require pre-specified models and based on its non-linearity and complexity, involves the estimation of many parameters (using Bayesian estimation) and thus considerably more processing time. Each region ultimately is characterized by a single parameter (neuronal activity) (Friston et al., 2003; Frässle et al., 2015). DCM is primarily used for task-based fMRI but can also be applied to rs-fMRI analyses (Friston et al., 2014a; Razi et al., 2015; Rigoux and Daunizeau, 2015).

Another method which may be used to investigate effective connectivity is Granger Causality Mapping (GCM) (Figure 2E). The process is based upon determining temporal precedence in neural time-series and infers causality from time-lagged correlation (Goebel et al., 2003; Friston et al., 2013; Seth et al., 2015). GCM does not require the specification of an *a priori* model, but does have significant limitations imposed by inherent latency differences in the HRF across different brain regions, low-sampling rates and noise (Wen et al., 2013). It has been applied both to task-based (Anderson et al., 2015) and rs-fMRI (Liao et al., 2011).

Enjoying increasing popularity, Multivoxel Pattern Analysis (MVPA) uses pattern-classification algorithms (classifiers) (Haynes, 2015) in the attempt to delineate different mental states, as well as to correlate the patterns with specific perceptual, cognitive, or disease states (Figure 2F) (Norman et al., 2006; Mahmoudi et al., 2012; Premi et al., 2016). In contrast to the standard GLM approach (focus on patterns of activity of individual voxels), MVPA incorporates the signal from the distributed activity or connectivity across multiple voxels simultaneously, allowing to infer mental states from patterns of distributed neural activity and the formulation of proper reverse inferences (Poldrack et al., 2009). Furthermore, enables a greater sensitivity and specificity, as well as the possibility to test hypothesis with designs that cannot be implemented in mass-univariate methods implemented with the standard GLM approach (Etzel et al., 2013). Another difference between the approaches relies on the fact that while *t*-tests model the complete set of time points, a classification trains on a subset of data (Coutanche, 2013). MVPA analyses are typically implemented using a “decoding” approach, which is based on the use of classifiers, such as neural networks (Polyn et al., 2005; Nickl-Jockschat et al., 2015), support vector machines (Meier et al., 2012; Månsson et al., 2015), and linear discriminant analysis (Cox and Savoy, 2003; Mandelkow et al., 2016), as a mean to differentiate between different classes or groups of individuals. Despite its popularity in the neuroimaging field, the “decoding” approach has some limitations, particularly related with the different results obtained with different parameters and/or algorithms. An alternative approach, the “searchlight” mapping, performs multivariate analysis on a spherical “searchlight” centered on each voxel in turn, resulting in a statistical map of local multivariate effects (Allefeld and Haynes, 2014), which can be interpreted similarly to a GLM statistics output map (Kriegeskorte et al., 2006). MVPA analyses can be applied both to task-based and rs-fMRI and, with their high sensitivity and effective use of spatial information, allow pattern detection of increasingly complex scenarios. On the other hand, the use of complex and specific classifiers may make it difficult to generalize the results of employing this technique (Dosenbach et al., 2010; Cole et al., 2013).

### Typical resting state analyses methods

Historically, the first method applied to rs-fMRI was seed-based correlational analysis (Figure 2G) (Biswal et al., 1995). The method is based on the activity in an *a priori* defined ROI (the seed region) which may be a volume or a single voxel, which is compared to that in all other voxels in the brain (Lee et al., 2013). Seed-based analyses are characterized by simple implementation and statistics and are straightforward to interpret, but do require an a priori selection of ROI. Such selection can be optimized using the data itself (Golestani and Goodyear, 2011). This form of analysis is widely used for rs-fMRI (each RSN can be extracted from a specific associated ROI), but can additionally be applied to fMRI tasks (Schurz et al., 2015) and to PPI analysis, which is in principle a seed-based analysis.

Regional Homogeneity analysis (ReHo) (Figure 2H) uses Kendall's coefficient of concordance to measure the synchronization between the time-series of each voxel and that of its nearest neighbors (based on a pre-defined ROI) (Zang et al., 2004). The ReHo method is easy to implement and interpret, and is normally applied to rs-fMRI determinations (Zang et al., 2007; Pedersen et al., 2015). The Amplitude of Low-Frequency Fluctuations (ALFF) and more recently, the fractional ALFF (fALFF, which has reduced sensitivity to physiological noise), measures signal magnitude on a voxel by voxel basis (Figure 2I) (Zou et al., 2008). ReHo and (f)ALFF both are methods which reflect properties of local spontaneous activity and, because they manifest different properties of the BOLD signal (synchronization and amplitude), they are usually implemented as complementary analyses.

In order to overcome the limitations of model-based analyses, exploratory data-driven methods, which require neither prior information nor a previously defined model, have been applied to fMRI. The three primary techniques are Principal Component Analysis (PCA), Independent Component Analysis (ICA), and clustering. PCA is a method built on finding a set of orthogonal axes (identified as principal components) that can maximize the explained variance of data and separate the relevant information from the noise (Figure 2J) (Wold et al., 1987; Viviani et al., 2005; Abdi and Williams, 2010; Smith et al., 2014). The efficacy of PCA is strongly dependent on assumptions of linearity, orthogonality of principal components, and high SNR. It can be applied both to task-based (Nomi et al., 2008) and rs-fMRI (Zhong et al., 2009). The method most frequently used for studies of rs-fMRI FC is ICA (an extension of PCA) (Figure 2K) (Jutten and Herault, 1991). This processing technique separates individual elements into their underlying components, and models the fMRI data set as a constant number of spatially or temporally independent components, which then are linearly mixed (Kiviniemi et al., 2003; Beckmann, 2012). For fMRI, ICA maps are normally generated using spatial ICA methods (spatially independent components) however temporal ICA also can be implemented and is used primarily for task fMRI. Limitations to the use of the technique in the temporal domain are its high computational demands and necessity of relying on fewer data points than studies considering spatial components (Calhoun et al., 2001). ICA generates a set of spatial maps and corresponding time-courses. The selection of components of interest is not trivial (in the absence of an a priori hypothesis) and is usually performed by visual inspection or correlation with a predefined RSN template. While straightforward to implement in single-subject analyses, group ICA analyses are more complex and require choosing between several different workflows and algorithm definitions (Beckmann and Smith, 2004; Calhoun et al., 2009; Schöpf et al., 2010; Du et al., 2016). ICA methods also have been used extensively in rs-fMRI studies (Beckmann et al., 2005; Soares et al., 2016), task-based fMRI (Calhoun et al., 2008), and for artifact removal (Perlbarg et al., 2007; Feis et al., 2015; Pruim et al., 2015).

The use of clustering methods constitutes a different approach based on mathematical algorithms that groups data into subsets (clusters) such that parameters of the same cluster are more similar to one another than they are to those of different clusters (Figure 2L). Similarly to PCA and ICA, clustering is a totally data-driven approach that enables, for example, the grouping of brain voxels with similar connectivity in the same cluster. The main difference relies on the fact that ICA assumes that there are spatially independent regions that form a network through a shared fMRI time-course, while clustering does not rely on assumptions and simply groups voxels with similar time-courses. Clustering methods have been successfully implemented both with rs-fMRI (Mezer et al., 2009; Lee et al., 2012) and task-based fMRI (Goutte et al., 1999; Heller et al., 2006). The major challenges associated are the requirements that the spatial reproducibility of networks be optimized across subjects and that individual network homogeneity be maximized (Shams et al., 2015). Clustering can be implemented using hierarchical techniques (Cordes et al., 2002), partitional clustering (such as k-means) (Fadili et al., 2000), spectral clustering approaches (Craddock et al., 2012), or sparse geostatistical analysis (Ye et al., 2011). Despite serving similar purposes as ICA, clustering methods were shown to outperform ICA for classification purposes (Meyer-Baese et al., 2004).

An increasingly prominent and powerful tool for the study of functional brain networks is graph theory. These methods model the brain as a network comprised of nodes (voxels or regions) and edges (connections between nodes, e.g., time-series correlations). This enables the establishment of functional interactions between every possible brain region, constituting an extension of the seed-based analysis where all possible seeds are explored, also known as the functional connectome. This whole-brain network is mathematically modeled as graph and, consequently, graph-theory metrics can be used to study the topological properties of such network (Figure 2M). Properties such as clustering-coefficient, characteristic path length, centrality, efficiency, modularity, among others, provide insights about functional integration, segregation, resilience or organization of the network as whole or of its individual nodes (Reijneveld et al., 2007; Stam and Reijneveld, 2007; Bullmore and Sporns, 2009). The approach has been used extensively with rs-fMRI (Wang et al., 2010; Ye et al., 2015; Marques et al., 2016) and, to a lesser extent, for task-based fMRI (Cao et al., 2014), where it has been described as sometimes difficult to implement and interpret (Fornito et al., 2013). Another approach, which is somewhat more straightforward to implement, is to characterize the edges of the graph, rather than to consider the topological properties of the entire network.

In contrast to most rs-fMRI strategies, which are based on the assumption of stationarity, dynamic functional connectivity (dFC) addresses the temporal component (fluctuations) of spontaneous BOLD signals (Figure 2N). Dynamic FC analysis has the potential to clarify the constant changes in patterns of neural activity and may be a more appropriate choice for the analysis of rs-fMRI studies (Bassett et al., 2011; Cabral et al., 2011; Madhyastha et al., 2015; Kaiser et al., 2016). The technique can be implemented using the sliding window correlations approach (most common) (Hindriks et al., 2016), time-frequency analysis (Chang and Glover, 2010), single-volume co-activation patterns (Liu et al., 2013), repeating sequences of BOLD activity (Pan et al., 2013), or through phase synchronization (Glerean et al., 2012). A number of limitations associated with the approach include the initial steps of sliding-window specification and specificity of pre-processing, as well as its sensitivity to physiological noise and complexity of the attendant statistical analysis (Hutchison et al., 2013; Leonardi and Van De Ville, 2015; Tagliazucchi and Laufs, 2015).

## Statistical analyses

In a single fMRI experiment, images made up of roughly 100,000 voxels are acquired from hundreds to thousands of times, resulting in a massive data set which has a complex spatial and temporal structure (Figure 1Q).

### Group-level analyses

In order to make inferences at the group-level (i.e., second-level), the analyses of fMRI data most widely used are performed within the GLM framework. In general terms, the GLM approach models the time series of the fMRI signal as a linear combination of different signal components, in order to test whether the activity in a defined brain region is systematically associated with a particular condition of interest (Lindquist, 2008). The GLM is expressed as:

$$\begin{array}{l} Y=X\;*\;\beta +\;\varepsilon \end{array}$$

where *Y* is the observed BOLD response, *X* corresponds to the design matrix, β is related with the parameter estimates and ϵ is the error. Hypothesis testing in the GLM framework include a set of parametric approaches, comprising the familiar *T*-Tests (independent and paired), Multiple Regression and ANalysis Of VAriance (ANOVA) (Friston et al., 2007). Commonly, the research question leads to more complex experimental designs which involve both within-subjects (e.g., condition A vs. B) and between-subjects (e.g., control vs. experimental group) factors. More than one within-subjects factor or the analysis of between-subjects factors, cannot be performed with the traditional tools in a single model. Even though most allow the parametrization of such models, the results can be invalid due to the inherent inability of the tools to incorporate all the factors into a single model (Chen et al., 2014). An alternative approach, the GLM Flex tool (Harvard Aging Brain Study, Martinos Center, MGH, Charlestown, MA, http://mrtools.mgh.harvard.edu/index.php/GLM_Flex) was developed. The tool can handle multiple within- and between-subjects' factors, while also modeling all the possible interactions between factors within the same model. Parametric tests are popular due to their simplicity and ease of application. However, these tests make some strong assumptions that are minimally met, or not met at all, in fMRI data sets (e.g., assumption of normality). As a result, it is often more appropriate to use non-parametric tests. Such tests estimate the null distribution from the data itself. The most common non-parametric tests used in fMRI analysis are permutation (randomization) tests. Tools that implement such tests include randomize from FSL (Winkler et al., 2014) and SnPM (Nichols and Holmes, 2002).

### Statistical significance

As in all standard statistical inference, the evaluation of fMRI data requires the establishment of a criterion for statistical significance. In early fMRI studies, the commonly-used standard for statistical significance was an uncorrected *p*-value of 0.001 at each voxel, a value that is 50 times more restrictive than that typically used in scientific research (Lieberman and Cunningham, 2009). In a typical fMRI experiment, more than 100,000 statistical tests may be performed (one test per voxel). Because this number of determinations is so great, a *p* < 0.001 would likely produce up to 100 voxels which would be erroneously identified as significant. Such a false-positive rate would clearly be unacceptable, so a variety of methods have been proposed to cope with the multiple comparisons issue. They can be divided into two main categories: voxel-based thresholding, including the family-wise error rate (FWER) and the false-discovery rate (FDR); and cluster-extent based thresholding (Forman et al., 1995). A widely used method for voxel-based thresholding consists of using the FWER in combination with Random Field Theory (RFT). The technique is implemented by estimating the smoothness of the image, expressed in the number of resels (image resolution element), since the neighboring voxels share statistical dependency. Although it can be thought of as roughly similar to the Bonferroni correction, FWER control using the RFT approach has a number of unique attributes and limitations due to the inherent smoothness of fMRI data. While enabling a great control over type I error, it often is over-conservative and may prevent true results from being detected (Hayasaka and Nichols, 2004). The FDR approach, another popular technique to control false-positives in neuroimaging studies (Genovese et al., 2002), considers the proportion of false positives in all the rejected tests. FDR control is less stringent than FWER and usually results in increased power. Because this approach is applied to *p*-values (rather than to the test statistics themselves) it can be used with any valid statistical test, but is highly dependent on the sample size. The most widely used FDR approach to functional imaging data is the Benjamini-Hochberg (BH) procedure, which assumes independence between tests (Benjamini and Hochberg, 1995). Statistical tests in fMRI are known to be dependent, however, so concern has been raised regarding its applicability (Chumbley and Friston, 2009; Chumbley et al., 2010). Most common software tools, specifically AFNI, FSL, and SPM, implement this type of correction method.

A significant problem associated with conservative approaches is the increased probability of committing type II errors (failure to detect true effects), particularly evident with small samples (Nichols and Hayasaka, 2003). It has also been postulated that this approach may favor the extraction of more obvious effects (such as sensorimotor processes), associated with signals of large magnitude. While failing to capture more subtle phenomena (such as complex cognitive and affective processes) often associated with signals of low amplitude (Lieberman and Cunningham, 2009), cluster-extent based thresholding has been put forward in order to address some of these shortcomings. It detects significant clusters based on the number of contiguous voxels that surpass a pre-determined primary threshold (Friston et al., 1994b). The main rationale for its use is that adjacent voxels are more likely to be involved in the same neuronal processes and thus are not independent (Smith and Nichols, 2009). The net result is that instead of estimating the false positive probability of each voxel, this approach estimates the false positive probability of the region as a whole (Woo et al., 2014). The cluster size is determined from the sampling distribution of the largest null cluster size under the null hypothesis of no signal. The reasoning behind this correction is based on the observation that false-positives are randomly distributed and thus are not likely to occur in contiguous groups of voxels (Woo et al., 2014). Cluster-extent approaches also are associated with reduced spatial specificity, describing the likelihood of finding a cluster of a given size or greater under the null hypothesis. The implication is that the larger the cluster, the less spatially specific the inference, though this is often an overlooked aspect of functional imaging (Woo et al., 2014). The most well-known cluster-size estimation methods are based on RFT as implemented in SPM, or on Monte Carlo simulations such as AlphaSim distributed with AFNI and with the REST toolbox. All these methods require the definition of an arbitrary primary cluster-defining threshold. An alternative method, termed threshold-free cluster enhancement (TFCE), was developed in order to eliminate the need for the definition of the primary threshold and is implemented in FSL (Smith and Nichols, 2009), CAT toolbox (http://dbm.neuro.uni-jena.de/cat/), and MatlabTFCE (https://github.com/markallenthornton/MatlabTFCE).

Yet another method of performing fMRI statistical analyses is through the use of specified ROIs. Analyses of this type are usually performed when the researcher has some *a priori* hypothesis regarding a specific brain region, which renders the previously discussed corrections for multiple comparisons too restrictive (see Poldrack, 2007 for other rationales). Generally, ROI analyses lead to an increased sensitivity (signal is average across groups of voxels) but a false sense of specificity of a given activation (activity patterns in regions outside the ROIs are masked out). The simplest approach consists of averaging the estimates over the voxels from the ROI and then performing the statistical testing with the averaged estimate. An alternative method, commonly named Small-Volume Correction (SVC), consists of restricting the voxel-wise analysis to the voxels inside the ROI, thus reducing the number of tests required to account for multiple comparisons corrections. Most software tools, such as SPM, FSL, and AFNI, contain routines for ROI-based analysis. The Marsbar tool (http://marsbar.sourceforge.net) for SPM was specifically developed for this purpose.

### Effect sizes

Contrary to the standard practice in other research areas, effect estimates (i.e., the effects' magnitude) are usually not provided in most neuroimaging reports. A recent publication highlighted that the statistic value does not provide information regarding the actual significance of the findings, serving rather as auxiliary evidence for the existence of the targeted effect. On the contrary, the effect estimate provides a clear picture of the property of interest and, consequently should be the focus of the investigation. For this reason, the absence or misreporting of effect-sizes has direct implication on the reliability and interpretability of fMRI findings (Chen et al., 2016). Taking this into consideration, it is strongly recommended that effect size maps/images are made available. With this practice, the whole range of effects and not only significant findings can be used to compare and properly aggregate effect sizes across different studies/research centers, and also allowing the use of power analysis in future studies (Poldrack et al., 2008).

### Meta-analysis

The number of fMRI publications continues to grow exponentially, but the results are often not consistent across studies (Radua and Mataix-Cols, 2012). Therefore, the meta-analysis of functional imaging studies may be essential for the continued development of new hypothesis about the neural mechanisms of cognition, emotion, and social processes (Wager et al., 2007). Individual studies generally provide evidence about brain activity rather than mental states, weather meta-analyses can help to identify consistently activated regions related to the same psychological state (Wager et al., 2007). Neuroimaging meta-analysis pools statistically significant results and offers the potential to improve predictive power, to build analytic tools and models, and to detect emergent properties of neural systems through large-scale data mining and computational modeling (Fox P. T. et al., 2014). The methods work by counting the number of activation peaks in each local brain area and comparing the observed number of peaks to a null-hypothesis distribution in order to establish a criterion for significance. Functional MRI meta-analysis can be performed using either full statistical parametric maps—image-based meta-analysis (IBMA)—or the coordinates of significant findings—coordinate-based meta-analysis (CBMA). Whereas, IBMA captures consistent patterns of brain activation across studies, even though these patterns are not identified as significant in individual studies, neuroimaging studies rarely provide full statistical parametric maps, which preclude these analyses. Thus, the majority of analysis aggregating neuroimaging results relies on CBMA, in which each eligible study included, reports using standard atlas or template based, 3-dimensional locations of peak activations. As a result, CBMA only aggregate results that are reported as significant across studies, and fail to capture individually non-significant, but consistent findings across different studies. A number of different algorithms have been developed for CMBA analyses, including the Activation Likelihood Estimation (ALE) (Eickhoff et al., 2012), Kernel Density Analysis (KDA) (Wager et al., 2004), Multi-level Kernel Density Analysis (MKDA) (Wager et al., 2007), and the Effect-size Signed-Differential-Mapping (ES-SDM) (Radua et al., 2012).

## Multimodal studies

Collecting multimodal brain data using different neuroimaging methods has become increasingly popular and is definitely a future trend, which provides an opportunity to develop a more global description of brain structure and function (Figure 1R). A number of different modalities and techniques have been used to complement fMRI analysis, either simultaneously or separated in time, and have been reviewed elsewhere (Biessmann et al., 2011; Uludag and Roebroeck, 2014; Liu et al., 2015a; Garcés et al., 2016). One particularly powerful approach to better understanding the brain is to model it as a network of functional connections between every possible region. The connectomic paradigm provides the investigator with an effective framework with which to study how dynamic changes in function are related to structural change, and how both are connected with brain states. Several extensive studies and worldwide projects [e.g., Human Connectome Project (Van Essen et al., 2013), Developing Human Connectome Project (http://www.developingconnectome.org/), Baby Connectome Project (http://www.fnih.org/what-we-do/current-research-programs/baby-connectome), or MyConnectome project (http://myconnectome.org/wp/)] are currently under way and have been enhancing multimodal approaches by combining fMRI data with structural information (e.g., diffusion data, volumetric data, cortical thickness, and voxel based morphometry) (Labudda et al., 2012; Crossley et al., 2014; Horn et al., 2014; Frank et al., 2016). Another approach employing complementary methodology is the combination of fMRI with the recording of brain electrical activity (electrophysiological response) using either electroencephalography (EEG) or magnetoencephalography (MEG) (Bledowski et al., 2004; Vaudano et al., 2012; Tewarie et al., 2015). Both techniques add improved temporal resolution to the very good spatial resolution of fMRI (Huster et al., 2012; Hall et al., 2014; Jorge et al., 2014). Positron emission tomography (PET) and single-photon emission computerized tomography (SPECT) both have a long history of providing fundamental information regarding brain metabolism. Though lacking the time resolution of fMRI, they complement that methodology by having the ability to study such parameters as neurotransmitter-receptor interactions and local glucose metabolism for longer periods in time (minutes) (Price, 2012; Sander et al., 2013; Tousseyn et al., 2015). It is possible to perform fMRI and functional near-infrared spectroscopy (fNIRS) simultaneously, and such a multimodal approach may be used to improve the temporal resolution of the former, thus allowing better correlation of the BOLD signal with local hemodynamic changes (Steinbrink et al., 2006; Sato et al., 2013). Inducing small direct currents in the brain using transcranial magnetic stimulation (TMS) or transcranial direct current stimulation (tDCS), make possible relatively focal excitation or inhibition and, when performed concurrently with fMRI, allows the study of functional interactions (Ruff et al., 2009; Peters et al., 2013; Weber et al., 2014; Leitão et al., 2015). The rapid growth of multimodal neuroimaging techniques has triggered the parallel development of computing methods and workflows capable of analyzing the resultant complex data sets (for review Liu et al., 2015b), and has led to the development of several tools dedicated to this type of study (Casanova et al., 2007; McFarquhar et al., 2016).

While the primary focus of this guide has been that of human neuroimaging, it is useful to note that many of the concepts and strategies described also can be applied to animal experimentation. The availability of ultra-high field scanners, capable of achieving very high resolution, has made feasible the application of fMRI to brains as small as that of a mouse (Jonckers et al., 2011; Schlegel et al., 2015). Other animals studied using this technique are rats (Liang et al., 2012; Henckens et al., 2015), non-human primates (Hutchison et al., 2015; Petkov et al., 2015), dogs (Andics et al., 2014; Berns et al., 2015), and cats (Brown et al., 2013; Hall et al., 2016). Translational research opportunities allow the investigator to develop animal models for studies which cannot be undertaken in patients or volunteers. A number of technical issues which must be considered when designing protocols for animal work are: the impact of higher magnetic fields and the ability to detect functional contrasts (Ciobanu et al., 2015); the use, or not, of anesthesia or sedation and its effects on regional and global brain activity (Kalthoff et al., 2013; Schlegel et al., 2015); physiological differences between animals and humans (Kalthoff et al., 2011; Sumiyoshi et al., 2012); and the fact that relatively few reference templates and atlases are available for animals (Stoewer et al., 2012; Nie et al., 2013; Papp et al., 2014).

## Report and interpretation of results

The results reported for a typical fMRI study include such information as the peak cluster coordinates (in x, y, and z), cluster size, the multiple comparisons correction method used, the statistical score (usually T-statistics or *Z*-values), and the brain regions of interest labeled with reference to a standard atlas and/or visual inspection. The correct interpretation of fMRI results is never straightforward and is dependent upon factors which range widely from the technical and methodological to the conceptual and statistical issues. Because there is such great variation in the manner with which studies are performed (Lange et al., 1999; Carp, 2012a; McGonigle, 2012), it is critically important that researchers/clinicians fully describe and report the methodological details as well as results, thus allowing replication as well as the potential incorporation of the findings into meta-analytic studies (Carp, 2012b). Comprehensive guidelines for reporting an fMRI study (Poldrack et al., 2008), as well as the principles of open and reproducible research for neuroimaging (Nichols et al., 2016) have been proposed, and have been accompanied by the development of a number of databases (Van Horn and Ishai, 2007; Poldrack and Gorgolewski, 2014; Poldrack and Poline, 2015). Specific examples of such data pools include OpenfMRI (Poldrack and Gorgolewski, 2015), ConnectomeDB (Hodge et al., 2016), Neuroinformatics Database (NiDB) (Book et al., 2016), or NeuroVault.org (Gorgolewski et al., 2016b). Effective communication of the results of fMRI investigations requires that the information has been organized and described in a clear and straightforward manner, using an unambiguous ontology (formal description of all terms and syntax) (Burns and Turner, 2013; Poldrack and Yarkoni, 2016) and format (Gorgolewski et al., 2016a).

The BOLD signal itself has a number of characteristics which present challenges to the accurate interpretation of fMRI data acquired with its use (Aguirre et al., 1998). BOLD responses are known to vary with different acquisition parameters (Renvall et al., 2014) and to be highly dependent on the specific parameters of neurovascular coupling which are known to vary with age, medication and in certain pathological states (D'Esposito et al., 2003; Bangen et al., 2009; Di et al., 2013; Tsvetanov et al., 2015). In addition, the nature of the BOLD signal has been shown to be affected by a variety of chemical compounds (e.g., caffeine and alcohol) (Levin et al., 1998; Mulderink et al., 2002; Perthen et al., 2008) as well as by respiration (Birn et al., 2008) and oxygen level (Cardenas et al., 2015).

The fundamental challenge of fMRI research is to draw conclusions which are completely supported by the data and which are unbiased. The literature contains numerous examples wherein foci of static regional activation are interpreted as associated with specific cognitive functions. Such empirical conclusions, termed “reverse inference” (Poldrack, 2006) are based on the implicit assumption that when a region of activation changes as a function of the performance of a specific task, that the region whose activity has changed is responsible for the associated cognitive process. This assumption fails to take into account either brain compensatory mechanisms (Sack et al., 2005; Meade et al., 2016) or plasticity (Poldrack, 2000; Colcombe et al., 2004; Amad et al., 2016). It is now generally accepted that a more complete description of brain function must include not only the notion of causality but also recognize the relationship between interconnected regions (network properties) through the characterization both of functional specialization (specific roles played by the different regions) and integration (how the regions interact with one another) (Van Horn and Poldrack, 2009). For all these reasons, drawing significant conclusions about mental states from fMRI data is challenging at best and the use of classification and predictive models such as machine learning algorithms have increasingly been tasked for this purpose (Pereira et al., 2009; Dosenbach et al., 2010).

As stated often throughout this guide, the statistical analysis of fMRI data is a complex process and great caution must be exercised when interpreting the experimental results. Questions have been raised, for example, about whether certain studies have reported findings able to be supported by the methodology used and the data obtained. Some studies purport to find extremely high degrees of correlation between individual behavioral characteristics (including personality, emotion, and social cognition) and specific regions of increased brain activity (Vul et al., 2009). Critics have pointed out that, considering the degree of methodological imprecision both of fMRI and in the measurement of individual characteristics, that the reported results may not be robust (Vul et al., 2009). Another issue is that of circular analysis, unfortunately seen with some frequency in functional studies. The issue arises when the data first are analyzed, subsets of those data selected, and then the same subsets are re-analyzed to obtain the results (Kriegeskorte et al., 2009). An fMRI example might be to define a ROI on the very basis of a statistical mapping which highlights the voxels of which it is composed in response to a functional activation state (Kriegeskorte et al., 2009). Such “double dipping,” the use of the same data for selection and subsequent selective analysis, results in an invalid statistical inference. It violates the criterion that the test statistics must be inherently independent of the selection criteria under the null hypothesis.

## Conclusions and future directions

Functional MRI currently is enjoying popularity in the study of brain function and promises to become even more prominent in the future. A number of factors contributing to the optimism about the expanding role of fMRI in neuroscience include: greater understanding of the BOLD and other contrast mechanisms; higher resolution and increased sensitivity; the use of new, more optimized preprocessing and analytic techniques; more powerful computational models; and extensive data sharing, enabling the design of studies comprised of large numbers of participants. Strategically, functional neuroimaging appears to be moving from the description and characterization of brain states toward predictive models of function based on the whole brain network. It is hoped that such models will incorporate behavioral features, genetic factors and biomarkers and will evolve to play an increasingly prominent clinical role in diagnosis, monitoring, and treatment of central nervous system disorders. In order to contribute to future progress, this article has sought to highlight the typical challenges faced when performing fMRI studies, and to offer some practical strategies with which they may be overcome. We have provided guidelines and references for the tools most commonly used at each step of the principal fMRI pipeline. As a concluding remark, we outline a set of general recommendations that we consider to be of upmost relevance for a better transparency and reproducibility of neuroimaging studies: before the study, perform a suitable experimental planning, including a proper design, power analysis (e.g., use the reported estimates as a means to estimate the adequate sample size) and identify the specific targets and analyses to be implemented; during the study, define the adequate acquisition protocols, identify as soon as possible and prevent the potential artifacts (in order to avoid losing data), carefully check the quality of the data, perform an accurate preprocessing, analysis and statistical testing and organize all the information in a standardized way, preferably with open-source software; after having the results, discuss them with caution, report them as well as the methodological details with great detail and following the guidelines (allowing study replication) and share the full statistical maps ideally in open repositories (allowing meta-analyses and power analyses for other similar studies). It is our hope that this guide will be of assistance both to those beginning to explore the potential of functional imaging as well as those who might appreciate a source book of current practice.

## Author contributions

JMS, RM, PSM, AS (Alexandre Sousa), and PM contributed in literature search, figures, study design, and writing. EG contributed in writing. AS (Adriana Sampaio), VA, and NS contributed in study design and writing.

### Conflict of interest statement

The reviewer BB and handling Editor declared their shared affiliation, and the handling Editor states that the process nevertheless met the standards of a fair and objective review. The other authors declare that the research was conducted in the absence of any commercial or financial relationships that could be construed as a potential conflict of interest.

## References

[B1] Abdi H. Williams L. J. (2010). Principal component analysis. Wiley Interdiscip. Rev. 2, 433–459. 10.1002/wics.10127570801

[B2] Aguirre G. K. Zarahn E. D'Esposito M. (1998). The variability of human, BOLD hemodynamic responses. Neuroimage 8, 360–369. 10.1006/nimg.1998.0369 9811554

[B3] Allefeld C. Haynes J. D. (2014). Searchlight-based multi-voxel pattern analysis of fMRI by cross-validated MANOVA. Neuroimage 89, 345–357. 10.1016/j.neuroimage.2013.11.043 24296330

[B4] Amad A. Seidman J. Draper S. B. Bruchhage M. M. Lowry R. G. Wheeler J.. (2016). Motor learning induces plasticity in the resting brain-drumming up a connection. Cereb. Cortex. 10.1093/cercor/bhw048. [Epub ahead of print]. 26941381

[B5] Amaro E. Jr. Barker G. J. (2006). Study design in fMRI: basic principles. Brain Cogn. 60, 220–232. 10.1016/j.bandc.2005.11.009 16427175

[B6] Andersen S. M. Rapcsak S. Z. Beeson P. M. (2010). Cost function masking during normalization of brains with focal lesions: still a necessity? Neuroimage 53, 78–84. 10.1016/j.neuroimage.2010.06.003 20542122 PMC2938189

[B7] Anderson B. Soliman S. O'Malley S. Danckert J. Besner D. (2015). Control over the strength of connections between modules: a double dissociation between stimulus format and task revealed by granger causality mapping in fMRI. Front. Psychol. 6:321. 10.3389/fpsyg.2015.00321 25870571 PMC4376120

[B8] Andics A. Gácsi M. Faragó T. Kis A. Miklósi A. (2014). Voice-sensitive regions in the dog and human brain are revealed by comparative fMRI. Curr. Biol. 24, 574–578. 10.1016/j.cub.2014.01.058 24560578

[B9] Anticevic A. Cole M. W. Murray J. D. Corlett P. R. Wang X. J. Krystal J. H. (2012). The role of default network deactivation in cognition and disease. Trends Cogn. Sci. 16, 584–592. 10.1016/j.tics.2012.10.008 23142417 PMC3501603

[B10] Ariely D. Berns G. S. (2010). Neuromarketing: the hope and hype of neuroimaging in business. Nat. Rev. Neurosci. 11, 284–292. 10.1038/nrn2795 20197790 PMC2875927

[B11] Aso T. Urayama S. Fukuyama H. Le Bihan D. (2013). Comparison of diffusion-weighted fMRI and BOLD fMRI responses in a verbal working memory task. Neuroimage 67, 25–32. 10.1016/j.neuroimage.2012.11.005 23147237

[B12] Aurich N. K. Alves Filho J. O. Marques da Silva A. M. Franco A. R. (2015). Evaluating the reliability of different preprocessing steps to estimate graph theoretical measures in resting state fMRI data. Front. Neurosci. 9:48. 10.3389/fnins.2015.00048 25745384 PMC4333797

[B13] Avants B. B. Tustison N. Song G. (2009). Advanced normalization tools (ANTS). Insight J. 2, 1–35. Available online at: ftp://ftp.ie.freshrpms.net/pub/sourceforge/a/project/ad/advants/ANTS/ANTS_1_9_x/ants.pdf

[B14] Balteau E. Hutton C. Weiskopf N. (2010). Improved shimming for fMRI specifically optimizing the local BOLD sensitivity. Neuroimage 49, 327–336. 10.1016/j.neuroimage.2009.08.010 19682587 PMC2775904

[B15] Bandettini P. A. (2012a). Sewer pipe, wire, epoxy, and finger tapping: the start of fMRI at the Medical College of Wisconsin. Neuroimage 62, 620–631. 10.1016/j.neuroimage.2011.10.044 22044784 PMC3303998

[B16] Bandettini P. A. (2012b). Twenty years of functional MRI: the science and the stories. Neuroimage 62, 575–588. 10.1016/j.neuroimage.2012.04.026 22542637

[B17] Bandettini P. A. Wong E. C. Hinks R. S. Tikofsky R. S. Hyde J. S. (1992). Time course EPI of human brain function during task activation. Magn. Reson. Med. 25, 390–397. 10.1002/mrm.1910250220 1614324

[B18] Bangen K. J. Restom K. Liu T. T. Jak A. J. Wierenga C. E. Salmon D. P.. (2009). Differential age effects on cerebral blood flow and BOLD response to encoding: associations with cognition and stroke risk. Neurobiol. Aging 30, 1276–1287. 10.1016/j.neurobiolaging.2007.11.012 18160181 PMC2804245

[B19] Bannister P. R. Michael Brady J. Jenkinson M. (2007). Integrating temporal information with a non-rigid method of motion correction for functional magnetic resonance images. Image Vis. Comput. 25, 311–320. 10.1016/j.imavis.2005.10.002

[B20] Barnett L. Seth A. K. (2014). The MVGC multivariate Granger causality toolbox: a new approach to Granger-causal inference. J. Neurosci. Methods 223, 50–68. 10.1016/j.jneumeth.2013.10.018 24200508

[B21] Bartés-Serrallonga M. Serra-Grabulosa J. M. Adan A. Falcón C. Bargalló N. Solé-Casals J. (2015). Smoothing FMRI Data Using an Adaptive Wiener Filter, in Computational Intelligence: International Joint Conference, IJCCI 2012 Barcelona, Revised Selected Papers, eds Madani K. Correia D. A. Rosa A. Filipe J. (Cham: Springer International Publishing), 321–332.

[B22] Bassett D. S. Wymbs N. F. Porter M. A. Mucha P. J. Carlson J. M. Grafton S. T. (2011). Dynamic reconfiguration of human brain networks during learning. Proc. Natl. Acad. Sci. U.S.A. 108, 7641–7646. 10.1073/pnas.1018985108 21502525 PMC3088578

[B23] Beall E. B. Lowe M. J. (2014). SimPACE: generating simulated motion corrupted BOLD data with synthetic-navigated acquisition for the development and evaluation of SLOMOCO: a new, highly effective slicewise motion correction. Neuroimage 101, 21–34. 10.1016/j.neuroimage.2014.06.038 24969568 PMC4165749

[B24] Beckmann C. F. (2012). Modelling with independent components. Neuroimage 62, 891–901. 10.1016/j.neuroimage.2012.02.020 22369997

[B25] Beckmann C. F. DeLuca M. Devlin J. T. Smith S. M. (2005). Investigations into resting-state connectivity using independent component analysis. Philos. Trans. R. Soc. Lond. B Biol. Sci. 360, 1001–1013. 10.1098/rstb.2005.1634 16087444 PMC1854918

[B26] Beckmann C. F. Smith S. M. (2004). Probabilistic independent component analysis for functional magnetic resonance imaging. IEEE Trans. Med. Imaging 23, 137–152. 10.1109/TMI.2003.822821 14964560

[B27] Behzadi Y. Restom K. Liau J. Liu T. T. (2007). A component based noise correction method (CompCor) for BOLD and perfusion based fMRI. Neuroimage 37, 90–101. 10.1016/j.neuroimage.2007.04.042 17560126 PMC2214855

[B28] Benjamini Y. Hochberg Y. (1995). Controlling the false discovery rate: a practical and powerful approach to multiple testing. J. R. Stat. Soc. B 57, 289–300.

[B29] Bennett C. M. Miller M. B. (2013). fMRI reliability: influences of task and experimental design. Cogn. Affect. Behav. Neurosci. 13, 690–702. 10.3758/s13415-013-0195-1 23934630

[B30] Berns G. S. Brooks A. M. Spivak M. (2015). Scent of the familiar: an fMRI study of canine brain responses to familiar and unfamiliar human and dog odors. Behav. Processes 110, 37–46. 10.1016/j.beproc.2014.02.011 24607363

[B31] Bernstein M. A. Huston J. III Ward H. A. (2006). Imaging artifacts at 3.0T. J. Magn. Reson. Imaging 24, 735–746. 10.1002/jmri.20698 16958057

[B32] Biessmann F. Plis S. Meinecke F. C. Eichele T. Müller K. R. (2011). Analysis of multimodal neuroimaging data. IEEE Rev. Biomed. Eng. 4, 26–58. 10.1109/RBME.2011.2170675 22273790

[B33] Birn R. M. (2012). The role of physiological noise in resting-state functional connectivity. Neuroimage 62, 864–870. 10.1016/j.neuroimage.2012.01.016 22245341 PMC13374118

[B34] Birn R. M. Molloy E. K. Patriat R. Parker T. Meier T. B. Kirk G. R.. (2013). The effect of scan length on the reliability of resting-state fMRI connectivity estimates. Neuroimage 83, 550–558. 10.1016/j.neuroimage.2013.05.099 23747458 PMC4104183

[B35] Birn R. M. Smith M. A. Jones T. B. Bandettini P. A. (2008). The respiration response function: the temporal dynamics of fMRI signal fluctuations related to changes in respiration. Neuroimage 40, 644–654. 10.1016/j.neuroimage.2007.11.059 18234517 PMC2533266

[B36] Biswal B. Yetkin F. Z. Haughton V. M. Hyde J. S. (1995). Functional connectivity in the motor cortex of resting human brain using echo-planar MRI. Magn. Reson. Med. 34, 537–541. 10.1002/mrm.1910340409 8524021

[B37] Blamire A. M. Ogawa S. Ugurbil K. Rothman D. McCarthy G. Ellermann J. M.. (1992). Dynamic mapping of the human visual cortex by high-speed magnetic resonance imaging. Proc. Natl. Acad. Sci. U.S.A. 89, 11069–11073. 10.1073/pnas.89.22.11069 1438317 PMC50485

[B38] Bledowski C. Prvulovic D. Goebel R. Zanella F. E. Linden D. E. (2004). Attentional systems in target and distractor processing: a combined ERP and fMRI study. Neuroimage 22, 530–540. 10.1016/j.neuroimage.2003.12.034 15193581

[B39] Blockley N. P. Griffeth V. E. Buxton R. B. (2012). A general analysis of calibrated BOLD methodology for measuring CMRO2 responses: comparison of a new approach with existing methods. Neuroimage 60, 279–289. 10.1016/j.neuroimage.2011.11.081 22155329 PMC3288960

[B40] Book G. A. Stevens M. C. Assaf M. Glahn D. C. Pearlson G. D. (2016). Neuroimaging data sharing on the neuroinformatics database platform. Neuroimage 124, 1089–1092. 10.1016/j.neuroimage.2015.04.022 25888923 PMC4608854

[B41] Bordier C. Dojat M. Micheaux P. L. D. (2011). Temporal and spatial independent component analysis for fMRI data sets embedded in the analyzeFMRI R Package. J. Stat. Softw. 1 10.18637/jss.v044.i09

[B42] Boyacioğlu R. Schulz J. Müller N. C. J. Koopmans P. J. Barth M. Norris D. G. (2014). Whole brain, high resolution multiband spin-echo EPI fMRI at 7 T: a comparison with gradient-echo EPI using a color-word Stroop task. Neuroimage 97, 142–150. 10.1016/j.neuroimage.2014.04.011 24736172

[B43] Brainard D. H. (1997). The psychophysics toolbox. Spat. Vis. 10, 433–436. 10.1163/156856897X00357 9176952

[B44] Brett M. Johnsrude I. S. Owen A. M. (2002). The problem of functional localization in the human brain. Nat. Rev. Neurosci. 3, 243–249. 10.1038/nrn756 11994756

[B45] Bright M. G. Murphy K. (2015). Is fMRI “noise” really noise? Resting state nuisance regressors remove variance with network structure. Neuroimage 114, 158–169. 10.1016/j.neuroimage.2015.03.070 25862264 PMC4461310

[B46] Brown T. A. Joanisse M. F. Gati J. S. Hughes S. M. Nixon P. L. Menon R. S.. (2013). Characterization of the blood-oxygen level-dependent (BOLD) response in cat auditory cortex using high-field fMRI. Neuroimage 64, 458–465. 10.1016/j.neuroimage.2012.09.034 23000258

[B47] Büchel C. Friston K. J. (1997). Modulation of connectivity in visual pathways by attention: cortical interactions evaluated with structural equation modelling and fMRI. Cereb. Cortex 7, 768–778. 10.1093/cercor/7.8.768 9408041

[B48] Bullmore E. (2012). The future of functional MRI in clinical medicine. Neuroimage 62, 1267–1271. 10.1016/j.neuroimage.2012.01.026 22261374

[B49] Bullmore E. Sporns O. (2009). Complex brain networks: graph theoretical analysis of structural and functional systems. Nat. Rev. Neurosci. 10, 186–198. 10.1038/nrn2575 19190637

[B50] Buracas G. T. Boynton G. M. (2002). Efficient design of event-related fMRI experiments using M-sequences. Neuroimage 16, 801–813. 10.1006/nimg.2002.1116 12169264

[B51] Burns G. A. Turner J. A. (2013). Modeling functional Magnetic Resonance Imaging (fMRI) experimental variables in the Ontology of Experimental Variables and Values (OoEVV). Neuroimage 82, 662–670. 10.1016/j.neuroimage.2013.05.024 23684873 PMC4474486

[B52] Bush G. Vogt B. A. Holmes J. Dale A. M. Greve D. Jenike M. A.. (2002). Dorsal anterior cingulate cortex: a role in reward-based decision making. Proc. Natl. Acad. Sci. U.S.A. 99, 523–528. 10.1073/pnas.012470999 11756669 PMC117593

[B53] Button K. S. Ioannidis J. P. Mokrysz C. Nosek B. A. Flint J. Robinson E. S.. (2013). Power failure: why small sample size undermines the reliability of neuroscience. Nat. Rev. Neurosci. 14, 365–376. 10.1038/nrn3475 23571845

[B54] Buxton R. B. (2009). Introduction to Functional Magnetic Resonance Imaging: Principles and Techniques. New York, NY: Cambridge University Press 10.1017/cbo9780511605505

[B55] Buxton R. B. Frank L. R. Wong E. C. Siewert B. Warach S. Edelman R. R. (1998). A general kinetic model for quantitative perfusion imaging with arterial spin labeling. Magn. Reson. Med. 40, 383–396. 10.1002/mrm.1910400308 9727941

[B56] Buxton R. B. Uludag K. Dubowitz D. J. Liu T. T. (2004). Modeling the hemodynamic response to brain activation. Neuroimage 23(Suppl. 1), S220–S233. 10.1016/j.neuroimage.2004.07.013 15501093

[B57] Cabeza R. (2001). Cognitive neuroscience of aging: contributions of functional neuroimaging. Scand. J. Psychol. 42, 277–286. 10.1111/1467-9450.00237 11501741

[B58] Cabezas M. Oliver A. Lladó X. Freixenet J. Cuadra M. B. (2011). A review of atlas-based segmentation for magnetic resonance brain images. Comput. Methods Programs Biomed. 104, e158–e177. 10.1016/j.cmpb.2011.07.015 21871688

[B59] Cabral J. Hugues E. Sporns O. Deco G. (2011). Role of local network oscillations in resting-state functional connectivity. Neuroimage 57, 130–139. 10.1016/j.neuroimage.2011.04.010 21511044

[B60] Calhoun V. D. Adali T. Pearlson G. D. Pekar J. J. (2001). Spatial and temporal independent component analysis of functional MRI data containing a pair of task-related waveforms. Hum. Brain Mapp. 13, 43–53. 10.1002/hbm.1024 11284046 PMC6871956

[B61] Calhoun V. D. Kiehl K. A. Pearlson G. D. (2008). Modulation of temporally coherent brain networks estimated using ICA at rest and during cognitive tasks. Hum. Brain Mapp. 29, 828–838. 10.1002/hbm.20581 18438867 PMC2649823

[B62] Calhoun V. D. Liu J. Adali T. (2009). A review of group ICA for fMRI data and ICA for joint inference of imaging, genetic, and ERP data. Neuroimage 45, S163–S172. 10.1016/j.neuroimage.2008.10.057 19059344 PMC2651152

[B63] Calhoun V. D. Stevens M. C. Pearlson G. D. Kiehl K. A. (2004). fMRI analysis with the general linear model: removal of latency-induced amplitude bias by incorporation of hemodynamic derivative terms. Neuroimage 22, 252–257. 10.1016/j.neuroimage.2003.12.029 15110015

[B64] Calhoun V. Golay X. Pearlson G. (2000). Improved fMRI slice timing correction: interpolation errors and wrap around effects, in Proceedings of 9th Annual Meeting of ISMRM (Denver), 810.

[B65] Calvo-Merino B. Glaser D. E. Grèzes J. Passingham R. E. Haggard P. (2005). Action observation and acquired motor skills: an FMRI study with expert dancers. Cereb. Cortex 15, 1243–1249. 10.1093/cercor/bhi007 15616133

[B66] Campbell-Washburn A. E. Atkinson D. Nagy Z. Chan R. W. Josephs O. Lythgoe M. F.. (2016). Using the robust principal component analysis algorithm to remove RF spike artifacts from MR images. Magn. Reson. Med. 75, 2517–2525. 10.1002/mrm.25851 26193125 PMC4720596

[B67] Canli T. Zhao Z. Desmond J. E. Kang E. Gross J. Gabrieli J. D. (2001). An fMRI study of personality influences on brain reactivity to emotional stimuli. Behav. Neurosci. 115, 33–42. 10.1037/0735-7044.115.1.33 11256451

[B68] Cao H. Plichta M. M. Schäfer A. Haddad L. Grimm O. Schneider M.. (2014). Test-retest reliability of fMRI-based graph theoretical properties during working memory, emotion processing, and resting state. Neuroimage 84, 888–900. 10.1016/j.neuroimage.2013.09.013 24055506

[B69] Cardenas D. P. Muir E. R. Huang S. Boley A. Lodge D. Duong T. Q. (2015). Functional MRI during hyperbaric oxygen: Effects of oxygen on neurovascular coupling and BOLD fMRI signals. Neuroimage 119, 382–389. 10.1016/j.neuroimage.2015.06.082 26143203 PMC4564303

[B70] Caria A. Sitaram R. Birbaumer N. (2012). Real-time fMRI: a tool for local brain regulation. Neuroscientist 18, 487–501. 10.1177/1073858411407205 21652587

[B71] Carlson T. A. Schrater P. He S. (2003). Patterns of activity in the categorical representations of objects. J. Cogn. Neurosci. 15, 704–717. 10.1162/jocn.2003.15.5.704 12965044

[B72] Carp J. (2012a). On the plurality of (methodological) worlds: estimating the analytic flexibility of FMRI experiments. Front. Neurosci. 6:149. 10.3389/fnins.2012.00149 23087605 PMC3468892

[B73] Carp J. (2012b). The secret lives of experiments: methods reporting in the fMRI literature. Neuroimage 63, 289–300. 10.1016/j.neuroimage.2012.07.004 22796459

[B74] Casanova R. Srikanth R. Baer A. Laurienti P. J. Burdette J. H. Hayasaka S.. (2007). Biological parametric mapping: A statistical toolbox for multimodality brain image analysis. Neuroimage 34, 137–143. 10.1016/j.neuroimage.2006.09.011 17070709 PMC1994117

[B75] Castellanos F. X. Di Martino A. Craddock R. C. Mehta A. D. Milham M. P. (2013). Clinical applications of the functional connectome. Neuroimage 80, 527–540. 10.1016/j.neuroimage.2013.04.083 23631991 PMC3809093

[B76] Centeno M. Koepp M. J. Vollmar C. Stretton J. Sidhu M. Michallef C.. (2014). Language dominance assessment in a bilingual population: validity of fMRI in the second language. Epilepsia 55, 1504–1511. 10.1111/epi.12757 25182478

[B77] Chang C. Glover G. H. (2010). Time-frequency dynamics of resting-state brain connectivity measured with fMRI. Neuroimage 50, 81–98. 10.1016/j.neuroimage.2009.12.011 20006716 PMC2827259

[B78] Chase H. W. Clark L. (2010). Gambling severity predicts midbrain response to near-miss outcomes. J. Neurosci. 30, 6180–6187. 10.1523/JNEUROSCI.5758-09.2010 20445043 PMC2929454

[B79] Chen G. Adleman N. E. Saad Z. S. Leibenluft E. Cox R. W. (2014). Applications of multivariate modeling to neuroimaging group analysis: a comprehensive alternative to univariate general linear model. Neuroimage 99, 571–588. 10.1016/j.neuroimage.2014.06.027 24954281 PMC4121851

[B80] Chen G. Taylor P. A. Cox R. W. (2016). Is the statistic value all we should care about in neuroimaging? *bioRxiv*. 10.1016/j.neuroimage.2016.09.066 PMC6591724 27729277

[B81] Chen J. E. Glover G. H. (2015). BOLD fractional contribution to resting-state functional connectivity above 0.1 Hz. Neuroimage 107, 207–218. 10.1016/j.neuroimage.2014.12.012 25497686 PMC4318656

[B82] Chen N. K. Wyrwicz A. M. (2004). Removal of EPI Nyquist ghost artifacts with two-dimensional phase correction. Magn. Reson. Med. 51, 1247–1253. 10.1002/mrm.20097 15170846

[B83] Chiacchiaretta P. Ferretti A. (2015). Resting state BOLD functional connectivity at 3T: spin echo versus gradient echo EPI. PLoS ONE 10:e0120398. 10.1371/journal.pone.0120398 25749359 PMC4352074

[B84] Choe A. S. Jones C. K. Joel S. E. Muschelli J. Belegu V. Caffo B. S.. (2015). Reproducibility and temporal structure in weekly resting-state fMRI over a period of 3.5 years. PLoS ONE 10:e0140134. 10.1371/journal.pone.0140134 26517540 PMC4627782

[B85] Chumbley J. R. Friston K. J. (2009). False discovery rate revisited: FDR and topological inference using Gaussian random fields. Neuroimage 44, 62–70. 10.1016/j.neuroimage.2008.05.021 18603449

[B86] Chumbley J. Worsley K. Flandin G. Friston K. (2010). Topological FDR for neuroimaging. Neuroimage 49, 3057–3064. 10.1016/j.neuroimage.2009.10.090 19944173 PMC3221040

[B87] Churchill N. W. Oder A. Abdi H. Tam F. Lee W. Thomas C.. (2012a). Optimizing preprocessing and analysis pipelines for single-subject fMRI. I. Standard temporal motion and physiological noise correction methods. Hum. Brain Mapp. 33, 609–627. 10.1002/hbm.21238 21455942 PMC4898950

[B88] Churchill N. W. Yourganov G. Oder A. Tam F. Graham S. J. Strother S. C. (2012b). Optimizing preprocessing and analysis pipelines for single-subject fMRI: 2. Interactions with ICA, PCA, task contrast and inter-subject heterogeneity. PLoS ONE 7:e31147. 10.1371/journal.pone.0031147 22383999 PMC3288007

[B89] Churchill N. W. Yourganov G. Spring R. Rasmussen P. M. Lee W. Ween J. E.. (2012c). PHYCAA: data-driven measurement and removal of physiological noise in BOLD fMRI. Neuroimage 59, 1299–1314. 10.1016/j.neuroimage.2011.08.021 21871573

[B90] Ciobanu L. Solomon E. Pyatigorskaya N. Roussel T. Le Bihan D. Frydman L. (2015). fMRI contrast at high and ultrahigh magnetic fields: insight from complementary methods. Neuroimage 113, 37–43. 10.1016/j.neuroimage.2015.03.018 25795340

[B91] Cohen J. Macwhinney B. Flatt M. Provost J. (1993). PsyScope: an interactive graphic system for designing and controlling experiments in the psychology laboratory using Macintosh computers. Behav. Res. Methods Instrum. Comput. 25, 257–271. 10.3758/BF03204507

[B92] Colcombe S. J. Kramer A. F. Erickson K. I. Scalf P. McAuley E. Cohen N. J.. (2004). Cardiovascular fitness, cortical plasticity, and aging. Proc. Natl. Acad. Sci. U.S.A. 101, 3316–3321. 10.1073/pnas.0400266101 14978288 PMC373255

[B93] Cole D. M. Smith S. M. Beckmann C. F. (2010). Advances and pitfalls in the analysis and interpretation of resting-state FMRI data. Front. Syst. Neurosci. 4:8. 10.3389/fnsys.2010.00008 20407579 PMC2854531

[B94] Cole M. W. Reynolds J. R. Power J. D. Repovs G. Anticevic A. Braver T. S. (2013). Multi-task connectivity reveals flexible hubs for adaptive task control. Nat. Neurosci. 16, 1348–1355. 10.1038/nn.3470 23892552 PMC3758404

[B95] Cordes D. Haughton V. Carew J. D. Arfanakis K. Maravilla K. (2002). Hierarchical clustering to measure connectivity in fMRI resting-state data. Magn. Reson. Imaging 20, 305–317. 10.1016/S0730-725X(02)00503-9 12165349

[B96] Cordes D. Haughton V. M. Arfanakis K. Carew J. D. Turski P. A. Moritz C. H.. (2001). Frequencies contributing to functional connectivity in the cerebral cortex in “resting-state” data. AJNR Am. J. Neuroradiol. 22, 1326–1333. 11498421 PMC7975218

[B97] Cordes D. Nandy R. R. Schafer S. Wager T. D. (2014). Characterization and reduction of cardiac- and respiratory-induced noise as a function of the sampling rate (TR) in fMRI. Neuroimage 89, 314–330. 10.1016/j.neuroimage.2013.12.013 24355483 PMC4209749

[B98] Cordes J. S. Mathiak K. A. Dyck M. Alawi E. M. Gaber T. J. Zepf F. D.. (2015). Cognitive and neural strategies during control of the anterior cingulate cortex by fMRI neurofeedback in patients with schizophrenia. Front. Behav. Neurosci. 9:169. 10.3389/fnbeh.2015.00169 26161073 PMC4480149

[B99] Coutanche M. N. (2013). Distinguishing multi-voxel patterns and mean activation: why, how, and what does it tell us? Cogn. Affect. Behav. Neurosci. 13, 667–673. 10.3758/s13415-013-0186-2 23857415

[B100] Cox D. D. Savoy R. L. (2003). Functional magnetic resonance imaging (fMRI) “brain reading”: detecting and classifying distributed patterns of fMRI activity in human visual cortex. Neuroimage 19, 261–270. 10.1016/S1053-8119(03)00049-1 12814577

[B101] Cox R. W. (1996). AFNI: software for analysis and visualization of functional magnetic resonance neuroimages. Comput. Biomed. Res. 29, 162–173. 10.1006/cbmr.1996.0014 8812068

[B102] Cox R. W. (2012). AFNI: what a long strange trip it's been. Neuroimage 62, 743–747. 10.1016/j.neuroimage.2011.08.056 21889996 PMC3246532

[B103] Craddock R. C. James G. A. Holtzheimer P. E. III Hu X. P. Mayberg H. S. (2012). A whole brain fMRI atlas generated via spatially constrained spectral clustering. Hum. Brain Mapp. 33, 1914–1928. 10.1002/hbm.21333 21769991 PMC3838923

[B104] Craddock R. C. Jbabdi S. Yan C. G. Vogelstein J. T. Castellanos F. X. Di Martino A.. (2013). Imaging human connectomes at the macroscale. Nat. Methods 10, 524–539. 10.1038/nmeth.2482 23722212 PMC4096321

[B105] Crinion J. Ashburner J. Leff A. Brett M. Price C. Friston K. (2007). Spatial normalization of lesioned brains: performance evaluation and impact on fMRI analyses. Neuroimage 37, 866–875. 10.1016/j.neuroimage.2007.04.065 17616402 PMC3223520

[B106] Crossley N. A. Mechelli A. Scott J. Carletti F. Fox P. T. McGuire P.. (2014). The hubs of the human connectome are generally implicated in the anatomy of brain disorders. Brain 137, 2382–2395. 10.1093/brain/awu132 25057133 PMC4107735

[B107] Curtis C. E. D'Esposito M. (2003). Persistent activity in the prefrontal cortex during working memory. Trends Cogn. Sci. 7, 415–423. 10.1016/S1364-6613(03)00197-9 12963473

[B108] Dale A. M. (1999). Optimal experimental design for event-related fMRI. Hum. Brain Mapp. 8, 109–114. 10524601 10.1002/(SICI)1097-0193(1999)8:2/3<109::AID-HBM7>3.0.CO;2-W PMC6873302

[B109] Dale A. M. Buckner R. L. (1997). Selective averaging of rapidly presented individual trials using fMRI. Hum. Brain Mapp. 5, 329–340. 20408237 10.1002/(SICI)1097-0193(1997)5:5<329::AID-HBM1>3.0.CO;2-5

[B110] Damoiseaux J. S. Rombouts S. A. Barkhof F. Scheltens P. Stam C. J. Smith S. M.. (2006). Consistent resting-state networks across healthy subjects. Proc. Natl. Acad. Sci. U.S.A. 103, 13848–13853. 10.1073/pnas.0601417103 16945915 PMC1564249

[B111] Davis T. L. Kwong K. K. Weisskoff R. M. Rosen B. R. (1998). Calibrated functional MRI: mapping the dynamics of oxidative metabolism. Proc. Natl. Acad. Sci. U.S.A. 95, 1834–1839. 10.1073/pnas.95.4.1834 9465103 PMC19199

[B112] Davis T. Poldrack R. A. (2013). Measuring neural representations with fMRI: practices and pitfalls. Ann. N.Y. Acad. Sci. 1296, 108–134. 10.1111/nyas.12156 23738883

[B113] Deckers R. H. van Gelderen P. Ries M. Barret O. Duyn J. H. Ikonomidou V. N.. (2006). An adaptive filter for suppression of cardiac and respiratory noise in MRI time series data. Neuroimage 33, 1072–1081. 10.1016/j.neuroimage.2006.08.006 17011214 PMC1716734

[B114] Desikan R. S. Ségonne F. Fischl B. Quinn B. T. Dickerson B. C. Blacker D.. (2006). An automated labeling system for subdividing the human cerebral cortex on MRI scans into gyral based regions of interest. Neuroimage 31, 968–980. 10.1016/j.neuroimage.2006.01.021 16530430

[B115] D'Esposito M. Deouell L. Y. Gazzaley A. (2003). Alterations in the BOLD fMRI signal with ageing and disease: a challenge for neuroimaging. Nat. Rev. Neurosci. 4, 863–872. 10.1038/nrn1246 14595398

[B116] Di X. Kannurpatti S. S. Rypma B. Biswal B. B. (2013). Calibrating BOLD fMRI activations with neurovascular and anatomical constraints. Cereb. Cortex 23, 255–263. 10.1093/cercor/bhs001 22345358 PMC3539449

[B117] Di X. Rypma B. Biswal B. B. (2014). Correspondence of executive function related functional and anatomical alterations in aging brain. Prog. Neuropsychopharmacol. Biol. Psychiatry 48, 41–50. 10.1016/j.pnpbp.2013.09.001 24036319 PMC3870052

[B118] Diedrichsen J. Shadmehr R. (2005). Detecting and adjusting for artifacts in fMRI time series data. Neuroimage 27, 624–634. 10.1016/j.neuroimage.2005.04.039 15975828 PMC1479857

[B119] Diers K. Weber F. Brocke B. Strobel A. Schönfeld S. (2014). Instructions matter: a comparison of baseline conditions for cognitive emotion regulation paradigms. Front. Psychol. 5:347. 10.3389/fpsyg.2014.00347 24808872 PMC4009445

[B120] Dosenbach N. U. F. Nardos B. Cohen A. L. Fair D. A. Power J. D. Church J. A.. (2010). Prediction of Individual Brain Maturity Using fMRI. Science 329, 1358–1361. 10.1126/science.1194144 20829489 PMC3135376

[B121] Dosenbach N. U. Visscher K. M. Palmer E. D. Miezin F. M. Wenger K. K. Kang H. C.. (2006). A core system for the implementation of task sets. Neuron 50, 799–812. 10.1016/j.neuron.2006.04.031 16731517 PMC3621133

[B122] Du W. Levin-Schwartz Y. Fu G. S. Ma S. Calhoun V. D. Adali T. (2016). The role of diversity in complex ICA algorithms for fMRI analysis. J. Neurosci. Methods 264, 129–135. 10.1016/j.jneumeth.2016.03.012 26993820 PMC4833547

[B123] Duff E. P. Vennart W. Wise R. G. Howard M. A. Harris R. E. Lee M.. (2015). Learning to identify CNS drug action and efficacy using multistudy fMRI data. Sci. Transl. Med. 7, 274ra216. 10.1126/scitranslmed.3008438 25673761

[B124] Durnez J. Degryse J. Moerkerke B. Seurinck R. Sochat V. Poldrack R. (2016). Power and sample size calculations for fMRI studies based on the prevalence of active peaks. bioRxiv.

[B125] Duyn J. H. (2012). The future of ultra-high field MRI and fMRI for study of the human brain. Neuroimage 62, 1241–1248. 10.1016/j.neuroimage.2011.10.065 22063093 PMC3389184

[B126] Edward V. Windischberger C. Cunnington R. Erdler M. Lanzenberger R. Mayer D.. (2000). Quantification of fMRI artifact reduction by a novel plaster cast head holder. Hum. Brain Mapp. 11, 207–213. 10.1002/1097-0193(200011)11:3<207::AID-HBM60>3.0.CO;2-J 11098798 PMC6871974

[B127] Eickhoff S. B. Bzdok D. Laird A. R. Kurth F. Fox P. T. (2012). Activation likelihood estimation meta-analysis revisited. Neuroimage 59, 2349–2361. 10.1016/j.neuroimage.2011.09.017 21963913 PMC3254820

[B128] Eklund A. Dufort P. Villani M. Laconte S. (2014). BROCCOLI: Software for fast fMRI analysis on many-core CPUs and GPUs. Front. Neuroinform. 8:24. 10.3389/fninf.2014.00024 24672471 PMC3953750

[B129] Emmert K. Kopel R. Sulzer J. Brühl A. B. Berman B. D. Linden D. E.. (2016). Meta-analysis of real-time fMRI neurofeedback studies using individual participant data: How is brain regulation mediated? Neuroimage 124, 806–812. 10.1016/j.neuroimage.2015.09.042 26419389

[B130] Ernst R. R. Anderson W. A. (1966). Application of fourier transform spectroscopy to magnetic resonance. Rev. Sci. Instrum. 37, 93–102. 10.1063/1.1719961

[B131] Escott E. J. Rubinstein D. (2003). Free DICOM image viewing and processing software for your desktop computer: what's available and what it can do for you. Radiographics 23, 1341–1357. 10.1148/rg.235035047 12975521

[B132] Etzel J. A. Zacks J. M. Braver T. S. (2013). Searchlight analysis: promise, pitfalls, and potential. Neuroimage 78, 261–269. 10.1016/j.neuroimage.2013.03.041 23558106 PMC3988828

[B133] Evans A. C. Janke A. L. Collins D. L. Baillet S. (2012). Brain templates and atlases. Neuroimage 62, 911–922. 10.1016/j.neuroimage.2012.01.024 22248580

[B134] Fadili M. J. Ruan S. Bloyet D. Mazoyer B. (2000). A multistep unsupervised fuzzy clustering analysis of fMRI time series. Hum. Brain Mapp. 10, 160–178. 10.1002/1097-0193(200008)10:43.0.CO;2-U 10949054 PMC6871966

[B135] Falahpour M. Refai H. Bodurka J. (2013). Subject specific BOLD fMRI respiratory and cardiac response functions obtained from global signal. Neuroimage 72, 252–264. 10.1016/j.neuroimage.2013.01.050 23376493

[B136] Faro S. H. Mohamed F. B. (2010). BOLD fMRI: A Guide to Functional Imaging for Neuroscientists. New York, NY: Springer.

[B137] Feinberg D. A. Moeller S. Smith S. M. Auerbach E. Ramanna S. Gunther M.. (2010). Multiplexed echo planar imaging for sub-second whole brain FMRI and fast diffusion imaging. PLoS ONE 5:e15710. 10.1371/journal.pone.0015710 21187930 PMC3004955

[B138] Feinberg D. A. Reese T. G. Wedeen V. J. (2002). Simultaneous echo refocusing in EPI. Magn. Reson. Med. 48, 1–5. 10.1002/mrm.10227 12111925

[B139] Feis R. A. Smith S. M. Filippini N. Douaud G. Dopper E. G. Heise V.. (2015). ICA-based artifact removal diminishes scan site differences in multi-center resting-state fMRI. Front. Neurosci. 9:395. 10.3389/fnins.2015.00395 26578859 PMC4621866

[B140] Figley C. R. Leitch J. K. Stroman P. W. (2010). In contrast to BOLD: signal enhancement by extravascular water protons as an alternative mechanism of endogenous fMRI signal change. Magn. Reson. Imaging 28, 1234–1243. 10.1016/j.mri.2010.01.005 20299173

[B141] Filippi M. (2009). fMRI Techniques and Protocols. New York, NY: Humana Press.

[B142] Finn E. S. Shen X. Scheinost D. Rosenberg M. D. Huang J. Chun M. M.. (2015). Functional connectome fingerprinting: identifying individuals using patterns of brain connectivity. Nat. Neurosci. 18, 1664–1671. 10.1038/nn.4135 26457551 PMC5008686

[B143] Fischl B. (2012). FreeSurfer. Neuroimage 62, 774–781. 10.1016/j.neuroimage.2012.01.021 22248573 PMC3685476

[B144] Fischl B. Sereno M. I. Tootell R. B. Dale A. M. (1999). High-resolution intersubject averaging and a coordinate system for the cortical surface. Hum. Brain Mapp. 8, 272–284. 10619420 10.1002/(SICI)1097-0193(1999)8:4<272::AID-HBM10>3.0.CO;2-4 PMC6873338

[B145] Fischmeister F. P. Hollinger I. Klinger N. Geissler A. Wurnig M. C. Matt E.. (2013). The benefits of skull stripping in the normalization of clinical fMRI data. Neuroimage Clin. 3, 369–380. 10.1016/j.nicl.2013.09.007 24273720 PMC3814956

[B146] Forman S. D. Cohen J. D. Fitzgerald M. Eddy W. F. Mintun M. A. Noll D. C. (1995). Improved assessment of significant activation in functional magnetic resonance imaging (fMRI): use of a cluster-size threshold. Magn. Reson. Med. 33, 636–647. 10.1002/mrm.1910330508 7596267

[B147] Formisano E. Linden D. E. Di Salle F. Trojano L. Esposito F. Sack A. T.. (2002). Tracking the mind's image in the brain I: time-resolved fMRI during visuospatial mental imagery. Neuron 35, 185–194. 10.1016/S0896-6273(02)00747-X 12123618

[B148] Formisano E. Salle F. D. Goebel R. (2005). Fundamentals of data analysis methods in functional MRI, in Advanced Image Processing in Magnetic Resonance Imaging, eds Landini L. Positano V. Santarelli M. F. (Boca Raton, FL: CRC Press), 481–503.

[B149] Fornito A. Zalesky A. Breakspear M. (2013). Graph analysis of the human connectome: promise, progress, and pitfalls. Neuroimage 80, 426–444. 10.1016/j.neuroimage.2013.04.087 23643999

[B150] Fox M. D. Buckner R. L. Liu H. Chakravarty M. M. Lozano A. M. Pascual-Leone A. (2014). Resting-state networks link invasive and noninvasive brain stimulation across diverse psychiatric and neurological diseases. Proc. Natl. Acad. Sci. U.S.A. 111, E4367–E4375. 10.1073/pnas.1405003111 25267639 PMC4205651

[B151] Fox M. D. Greicius M. (2010). Clinical applications of resting state functional connectivity. Front. Syst. Neurosci. 4:19. 10.3389/fnsys.2010.00019 20592951 PMC2893721

[B152] Fox M. D. Raichle M. E. (2007). Spontaneous fluctuations in brain activity observed with functional magnetic resonance imaging. Nat. Rev. Neurosci. 8, 700–711. 10.1038/nrn2201 17704812

[B153] Fox M. D. Snyder A. Z. Vincent J. L. Corbetta M. Van Essen D. C. Raichle M. E. (2005). The human brain is intrinsically organized into dynamic, anticorrelated functional networks. Proc. Natl. Acad. Sci. U.S.A. 102, 9673–9678. 10.1073/pnas.0504136102 15976020 PMC1157105

[B154] Fox P. T. Lancaster J. L. Laird A. R. Eickhoff S. B. (2014). Meta-analysis in human neuroimaging: computational modeling of large-scale databases. Annu. Rev. Neurosci. 37, 409–434. 10.1146/annurev-neuro-062012-170320 25032500 PMC4782802

[B155] Frank S. M. Reavis E. A. Greenlee M. W. Tse P. U. (2016). Pretraining cortical thickness predicts subsequent perceptual learning rate in a visual search task. Cereb. Cortex 26, 1211–1220. 10.1093/cercor/bhu309 25576537

[B156] Fransson P. (2005). Spontaneous low-frequency BOLD signal fluctuations: an fMRI investigation of the resting-state default mode of brain function hypothesis. Hum. Brain Mapp. 26, 15–29. 10.1002/hbm.20113 15852468 PMC6871700

[B157] Frässle S. Stephan K. E. Friston K. J. Steup M. Krach S. Paulus F. M.. (2015). Test-retest reliability of dynamic causal modeling for fMRI. Neuroimage 117, 56–66. 10.1016/j.neuroimage.2015.05.040 26004501

[B158] Friedman L. Glover G. H. (2006). Report on a multicenter fMRI quality assurance protocol. J. Magn. Reson. Imaging 23, 827–839. 10.1002/jmri.20583 16649196

[B159] Friman O. Borga M. Lundberg P. Knutsson H. (2004). Detection and detrending in fMRI data analysis. Neuroimage 22, 645–655. 10.1016/j.neuroimage.2004.01.033 15193593

[B160] Friston K. (2009). Causal modelling and brain connectivity in functional magnetic resonance imaging. PLoS Biol. 7:e1000033. 10.1371/journal.pbio.1000033 19226186 PMC2642881

[B161] Friston K. (2012). Ten ironic rules for non-statistical reviewers. Neuroimage 61, 1300–1310. 10.1016/j.neuroimage.2012.04.018 22521475

[B162] Friston K. J. (2005). Models of brain function in neuroimaging. Annu. Rev. Psychol. 56, 57–87. 10.1146/annurev.psych.56.091103.070311 15709929

[B163] Friston K. J. (2011). Functional and effective connectivity: a review. Brain Connect. 1, 13–36. 10.1089/brain.2011.0008 22432952

[B164] Friston K. J. Ashburner J. T. Kiebel S. J. Nichols T. E. Penny W. D. (2007). Statistical Parametric Mapping: The Analysis of Functional Brain Images. Amsterdam; Boston, MA: Academic Press.

[B165] Friston K. J. Buechel C. Fink G. R. Morris J. Rolls E. Dolan R. J. (1997). Psychophysiological and modulatory interactions in neuroimaging. Neuroimage 6, 218–229. 10.1006/nimg.1997.0291 9344826

[B166] Friston K. J. Harrison L. Penny W. (2003). Dynamic causal modelling. Neuroimage 19, 1273–1302. 10.1016/S1053-8119(03)00202-7 12948688

[B167] Friston K. J. Holmes A. P. Worsley K. J. Poline J. P. Frith C. D. Frackowiak R. S. (1994a). Statistical parametric maps in functional imaging: a general linear approach. Hum. Brain Mapp. 2, 189–210.

[B168] Friston K. J. Josephs O. Rees G. Turner R. (1998). Nonlinear event-related responses in fMRI. Magn. Reson. Med. 39, 41–52. 10.1002/mrm.1910390109 9438436

[B169] Friston K. J. Kahan J. Biswal B. Razi A. (2014a). A DCM for resting state fMRI. Neuroimage 94, 396–407. 10.1016/j.neuroimage.2013.12.009 24345387 PMC4073651

[B170] Friston K. J. Kahan J. Razi A. Stephan K. E. Sporns O. (2014b). On nodes and modes in resting state fMRI. Neuroimage 99, 533–547. 10.1016/j.neuroimage.2014.05.056 24862075 PMC4121089

[B171] Friston K. J. Williams S. Howard R. Frackowiak R. S. Turner R. (1996). Movement-related effects in fMRI time-series. Magn. Reson. Med. 35, 346–355. 10.1002/mrm.1910350312 8699946

[B172] Friston K. J. Worsley K. J. Frackowiak R. S. J. Mazziotta J. C. Evans A. C. (1994b). Assessing the significance of focal activations using their spatial extent. Hum. Brain Mapp. 1, 210–220. 24578041 10.1002/hbm.460010306

[B173] Friston K. Moran R. Seth A. K. (2013). Analysing connectivity with Granger causality and dynamic causal modelling. Curr. Opin. Neurobiol. 23, 172–178. 10.1016/j.conb.2012.11.010 23265964 PMC3925802

[B174] Ganger S. Hahn A. Küblböck M. Kranz G. S. Spies M. Vanicek T.. (2015). Comparison of continuously acquired resting state and extracted analogues from active tasks. Hum. Brain Mapp. 36, 4053–4063. 10.1002/hbm.22897 26178250 PMC4950683

[B175] Garcés P. Pereda E. Hernández-Tamames J. A. Del-Pozo F. Maestú F. Angel Pineda-Pardo J. Á. (2016). Multimodal description of whole brain connectivity: A comparison of resting state MEG, fMRI, and DWI. Hum. Brain Mapp. 37, 20–34. 10.1002/hbm.22995 26503502 PMC5132061

[B176] Gates K. M. Molenaar P. C. (2012). Group search algorithm recovers effective connectivity maps for individuals in homogeneous and heterogeneous samples. Neuroimage 63, 310–319. 10.1016/j.neuroimage.2012.06.026 22732562

[B177] Geissler A. Lanzenberger R. Barth M. Tahamtan A. R. Milakara D. Gartus A.. (2005). Influence of fMRI smoothing procedures on replicability of fine scale motor localization. Neuroimage 24, 323–331. 10.1016/j.neuroimage.2004.08.042 15627575

[B178] Genovese C. R. Lazar N. A. Nichols T. (2002). Thresholding of statistical maps in functional neuroimaging using the false discovery rate. Neuroimage 15, 870–878. 10.1006/nimg.2001.1037 11906227

[B179] Gitelman D. R. Penny W. D. Ashburner J. Friston K. J. (2003). Modeling regional and psychophysiologic interactions in fMRI: the importance of hemodynamic deconvolution. Neuroimage 19, 200–207. 10.1016/S1053-8119(03)00058-2 12781739

[B180] Glerean E. Salmi J. Lahnakoski J. M. Jääskeläinen I. P. Sams M. (2012). Functional magnetic resonance imaging phase synchronization as a measure of dynamic functional connectivity. Brain Connect. 2, 91–101. 10.1089/brain.2011.0068 22559794 PMC3624768

[B181] Glover G. H. Li T. Q. Ress D. (2000). Image-based method for retrospective correction of physiological motion effects in fMRI: RETROICOR. Magn. Reson. Med. 44, 162–167. 10.1002/1522-2594(200007)44:1<162::AID-MRM23>3.0.CO;2-E 10893535

[B182] Glover G. H. Mueller B. A. Turner J. A. van Erp T. G. Liu T. T. Greve D. N.. (2012). Function biomedical informatics research network recommendations for prospective multicenter functional MRI studies. J. Magn. Reson. Imaging 36, 39–54. 10.1002/jmri.23572 22314879 PMC3349791

[B183] Goebel R. (2012). BrainVoyager - past, present, future. Neuroimage 62, 748–756. 10.1016/j.neuroimage.2012.01.083 22289803

[B184] Goebel R. Roebroeck A. Kim D.-S. Formisano E. (2003). Investigating directed cortical interactions in time-resolved fMRI data using vector autoregressive modeling and Granger causality mapping. Magn. Reson. Imaging 21, 1251–1261. 10.1016/j.mri.2003.08.026 14725933

[B185] Gohel S. R. Biswal B. B. (2015). Functional integration between brain regions at rest occurs in multiple-frequency bands. Brain Connect. 5, 23–34. 10.1089/brain.2013.0210 24702246 PMC4313418

[B186] Golestani A. M. Goodyear B. G. (2011). Regions of interest for resting-state fMRI analysis determined by inter-voxel cross-correlation. Neuroimage 56, 246–251. 10.1016/j.neuroimage.2011.02.038 21338691

[B187] Gonzalez-Castillo J. Roopchansingh V. Bandettini P. A. Bodurka J. (2011). Physiological noise effects on the flip angle selection in BOLD fMRI. Neuroimage 54, 2764–2778. 10.1016/j.neuroimage.2010.11.020 21073963 PMC3020268

[B188] Gore J. C. (2003). Principles and practice of functional MRI of the human brain. J. Clin. Invest. 112, 4–9. 10.1172/JCI200319010 12840051 PMC162295

[B189] Gorgolewski K. Burns C. D. Madison C. Clark D. Halchenko Y. O. Waskom M. L.. (2011). Nipype: a flexible, lightweight and extensible neuroimaging data processing framework in python. Front. Neuroinform. 5:13. 10.3389/fninf.2011.00013 21897815 PMC3159964

[B190] Gorgolewski K. J. Auer T. Calhoun V. D. Craddock R. C. Das S. Duff E. P.. (2016a). The brain imaging data structure, a format for organizing and describing outputs of neuroimaging experiments. Sci. Data 3, 160044. 10.1038/sdata.2016.44 27326542 PMC4978148

[B191] Gorgolewski K. J. Varoquaux G. Rivera G. Schwartz Y. Sochat V. V. Ghosh S. S.. (2016b). NeuroVault.org: A repository for sharing unthresholded statistical maps, parcellations, and atlases of the human brain. Neuroimage 124, 1242–1244. 10.1016/j.neuroimage.2015.04.016 25869863 PMC4806527

[B192] Gorno-Tempini M. L. Hutton C. Josephs O. Deichmann R. Price C. Turner R. (2002). Echo time dependence of BOLD contrast and susceptibility artifacts. Neuroimage 15, 136–142. 10.1006/nimg.2001.0967 11771981

[B193] Goutte C. Toft P. Rostrup E. Nielsen F. Hansen L. K. (1999). On clustering fMRI time series. Neuroimage 9, 298–310. 10.1006/nimg.1998.0391 10075900

[B194] Greicius M. D. Menon V. (2004). Default-mode activity during a passive sensory task: uncoupled from deactivation but impacting activation. J. Cogn. Neurosci. 16, 1484–1492. 10.1162/0898929042568532 15601513

[B195] Griffanti L. Salimi-Khorshidi G. Beckmann C. F. Auerbach E. J. Douaud G. Sexton C. E.. (2014). ICA-based artefact removal and accelerated fMRI acquisition for improved resting state network imaging. Neuroimage 95, 232–247. 10.1016/j.neuroimage.2014.03.034 24657355 PMC4154346

[B196] Griswold M. A. Jakob P. M. Heidemann R. M. Nittka M. Jellus V. Wang J.. (2002). Generalized autocalibrating partially parallel acquisitions (GRAPPA). Magn. Reson. Med. 47, 1202–1210. 10.1002/mrm.10171 12111967

[B197] Guo Q. Hall G. McKinnon M. Thabane L. Goeree R. Pullenayegum E. (2012). Setting sample size using cost efficiency in fMRI studies. Open Access Medical Statistics 2, 33–41. 10.2147/OAMS.S30830

[B198] Halai A. D. Welbourne S. R. Embleton K. Parkes L. M. (2014). A comparison of dual gradient-echo and spin-echo fMRI of the inferior temporal lobe. Hum. Brain Mapp. 35, 4118–4128. 10.1002/hbm.22463 24677506 PMC6869502

[B199] Hall A. J. Butler B. E. Lomber S. G. (2016). The cat's meow: A high-field fMRI assessment of cortical activity in response to vocalizations and complex auditory stimuli. Neuroimage 127, 44–57. 10.1016/j.neuroimage.2015.11.056 26658927

[B200] Hall E. L. Robson S. E. Morris P. G. Brookes M. J. (2014). The relationship between MEG and fMRI. Neuroimage 102(Pt 1), 80–91. 10.1016/j.neuroimage.2013.11.005 24239589

[B201] Haller S. Bartsch A. J. (2009). Pitfalls in FMRI. Eur. Radiol. 19, 2689–2706. 10.1007/s00330-009-1456-9 19504107

[B202] Handwerker D. A. Gonzalez-Castillo J. D'Esposito M. Bandettini P. A. (2012). The continuing challenge of understanding and modeling hemodynamic variation in fMRI. Neuroimage 62, 1017–1023. 10.1016/j.neuroimage.2012.02.015 22366081 PMC4180210

[B203] Hanke M. Halchenko Y. O. Sederberg P. B. Hanson S. J. Haxby J. V. Pollmann S. (2009). PyMVPA: A python toolbox for multivariate pattern analysis of fMRI data. Neuroinformatics 7, 37–53. 10.1007/s12021-008-9041-y 19184561 PMC2664559

[B204] Hashemi R. H. Bradley W. G. Lisanti C. J. (2012). MRI: The Basics. Philadelphia, PA: Wolters Kluwer Health.

[B205] Hayasaka S. Nichols T. E. (2004). Combining voxel intensity and cluster extent with permutation test framework. Neuroimage 23, 54–63. 10.1016/j.neuroimage.2004.04.035 15325352

[B206] Haynes J. D. (2015). A primer on pattern-based approaches to fMRI: principles, pitfalls, and perspectives. Neuron 87, 257–270. 10.1016/j.neuron.2015.05.025 26182413

[B207] Hebart M. N. Görgen K. Haynes J. D. (2014). The Decoding Toolbox (TDT): a versatile software package for multivariate analyses of functional imaging data. Front. Neuroinform. 8:88. 10.3389/fninf.2014.00088 25610393 PMC4285115

[B208] Heim S. Amunts K. Mohlberg H. Wilms M. Friederici A. D. (2006). Head motion during overt language production in functional magnetic resonance imaging. Neuroreport 17, 579–582. 10.1097/00001756-200604240-00005 16603915

[B209] Heller R. Stanley D. Yekutieli D. Rubin N. Benjamini Y. (2006). Cluster-based analysis of FMRI data. Neuroimage 33, 599–608. 10.1016/j.neuroimage.2006.04.233 16952467

[B210] Henckens M. J. van der Marel K. van der Toorn A. Pillai A. G. Fernández G. Dijkhuizen R. M.. (2015). Stress-induced alterations in large-scale functional networks of the rodent brain. Neuroimage 105, 312–322. 10.1016/j.neuroimage.2014.10.037 25462693

[B211] Henson R. N. A. Büchel C. Josephs O. Friston K. (1999). The slice-timing problem in event-related fMRI. Neuroimage 9, 125. 10.1006/nimg.1999.0498 10547338

[B212] Hindriks R. Adhikari M. H. Murayama Y. Ganzetti M. Mantini D. Logothetis N. K.. (2016). Can sliding-window correlations reveal dynamic functional connectivity in resting-state fMRI? Neuroimage 127, 242–256. 10.1016/j.neuroimage.2015.11.055 26631813 PMC4758830

[B213] Hodge M. R. Horton W. Brown T. Herrick R. Olsen T. Hileman M. E.. (2016). ConnectomeDB–Sharing human brain connectivity data. Neuroimage 124, 1102–1107. 10.1016/j.neuroimage.2015.04.046 25934470 PMC4626437

[B214] Hoge W. S. Polimeni J. R. (2015). Dual-polarity GRAPPA for simultaneous reconstruction and ghost correction of echo planar imaging data. Magn. Reson. Med. 76, 32–44. 10.1002/mrm.25839 26208304 PMC4758917

[B215] Holland D. Kuperman J. M. Dale A. M. (2010). Efficient correction of inhomogeneous static magnetic field-induced distortion in Echo Planar Imaging. Neuroimage 50, 175–183. 10.1016/j.neuroimage.2009.11.044 19944768 PMC2819607

[B216] Horn A. Ostwald D. Reisert M. Blankenburg F. (2014). The structural-functional connectome and the default mode network of the human brain. Neuroimage 102(Pt 1), 142–151. 10.1016/j.neuroimage.2013.09.069 24099851

[B217] Horwitz B. Warner B. Fitzer J. Tagamets M. A. Husain F. T. Long T. W. (2005). Investigating the neural basis for functional and effective connectivity. Application to fMRI. Philos. Trans. R. Soc. Lond. B Biol. Sci. 360, 1093–1108. 10.1098/rstb.2005.1647 16087450 PMC1854930

[B218] Howseman A. M. Grootoonk S. Porter D. A. Ramdeen J. Holmes A. P. Turner R. (1999). The effect of slice order and thickness on fMRI activation data using multislice echo-planar imaging. Neuroimage 9, 363–376. 10.1006/nimg.1998.0418 10191165

[B219] Hu X. Yacoub E. (2012). The story of the initial dip in fMRI. Neuroimage 62, 1103–1108. 10.1016/j.neuroimage.2012.03.005 22426348 PMC3389272

[B220] Huettel S. A. (2012). Event-related fMRI in cognition. Neuroimage 62, 1152–1156. 10.1016/j.neuroimage.2011.08.113 21963919 PMC3277683

[B221] Hui M. Li R. Chen K. Jin Z. Yao L. Long Z. (2013). Improved estimation of the number of independent components for functional magnetic resonance data by a whitening filter. IEEE J. Biomed. Health Inform. 17, 629–641. 10.1109/JBHI.2013.2253560 24592464

[B222] Huster R. J. Debener S. Eichele T. Herrmann C. S. (2012). Methods for simultaneous EEG-fMRI: an introductory review. J. Neurosci. 32, 6053–6060. 10.1523/JNEUROSCI.0447-12.2012 22553012 PMC6622140

[B223] Hutchison R. M. Culham J. C. Flanagan J. R. Everling S. Gallivan J. P. (2015). Functional subdivisions of medial parieto-occipital cortex in humans and nonhuman primates using resting-state fMRI. Neuroimage 116, 10–29. 10.1016/j.neuroimage.2015.04.068 25970649

[B224] Hutchison R. M. Womelsdorf T. Allen E. A. Bandettini P. A. Calhoun V. D. Corbetta M.. (2013). Dynamic functional connectivity: promise, issues, and interpretations. Neuroimage 80, 360–378. 10.1016/j.neuroimage.2013.05.079 23707587 PMC3807588

[B225] Hutton C. Bork A. Josephs O. Deichmann R. Ashburner J. Turner R. (2002). Image distortion correction in fMRI: A quantitative evaluation. Neuroimage 16, 217–240. 10.1006/nimg.2001.1054 11969330

[B226] Hutton C. Josephs O. Stadler J. Featherstone E. Reid A. Speck O.. (2011). The impact of physiological noise correction on fMRI at 7 T. Neuroimage 57, 101–112. 10.1016/j.neuroimage.2011.04.018 21515386 PMC3115139

[B227] In M.-H. Posnansky O. Beall E. B. Lowe M. J. Speck O. (2015). Distortion correction in EPI using an extended PSF method with a reversed phase gradient approach. PLoS ONE 10:e0116320. 10.1371/journal.pone.0116320 25707006 PMC4338274

[B228] Inglis B. (2015). A Checklist for fMRI Acquisition Methods Reporting in the Literature. Berkeley: The Winnower.

[B229] Ioannidis J. P. (2008). Why most discovered true associations are inflated. Epidemiology 19, 640–648. 10.1097/EDE.0b013e31818131e7 18633328

[B230] James G. A. Kelley M. E. Craddock R. C. Holtzheimer P. E. Dunlop B. W. Nemeroff C. B.. (2009). Exploratory structural equation modeling of resting-state fMRI: applicability of group models to individual subjects. Neuroimage 45, 778–787. 10.1016/j.neuroimage.2008.12.049 19162206 PMC2653594

[B231] Jenkinson M. Beckmann C. F. Behrens T. E. Woolrich M. W. Smith S. M. (2012). Fsl. Neuroimage 62, 782–790. 10.1016/j.neuroimage.2011.09.015 21979382

[B232] Jenkinson M. Smith S. (2001). A global optimisation method for robust affine registration of brain images. Med. Image Anal. 5, 143–156. 10.1016/S1361-8415(01)00036-6 11516708

[B233] Jezzard P. Song A. W. (1996). Technical foundations and pitfalls of clinical fMRI. Neuroimage 4, S63–75. 10.1006/nimg.1996.0056 9345530

[B234] Jiang A. Kennedy D. N. Baker J. R. Weisskoff R. M. Tootell R. B. H. Woods R. P. (1995). Motion detection and correction in functional MR imaging. Hum. Brain Mapp. 3, 224–235. 10.1002/hbm.460030306

[B235] Jo H. J. Gotts S. J. Reynolds R. C. Bandettini P. A. Martin A. Cox R. W.. (2013). Effective preprocessing procedures virtually eliminate distance-dependent motion artifacts in resting state fMRI. J. Appl. Math. 2013:935154. 10.1155/2013/935154 24415902 PMC3886863

[B236] Johnstone T. Ores Walsh K. S. Greischar L. L. Alexander A. L. Fox A. S. Davidson R. J.. (2006). Motion correction and the use of motion covariates in multiple-subject fMRI analysis. Hum. Brain Mapp. 27, 779–788. 10.1002/hbm.20219 16456818 PMC6871380

[B237] Jonckers E. Van Audekerke J. De Visscher G. Van der Linden A. Verhoye M. (2011). Functional connectivity fMRI of the rodent brain: comparison of functional connectivity networks in rat and mouse. PLoS ONE 6:e18876. 10.1371/journal.pone.0018876 21533116 PMC3078931

[B238] Jorge J. van der Zwaag W. Figueiredo P. (2014). EEG-fMRI integration for the study of human brain function. Neuroimage 102(Pt 1), 24–34. 10.1016/j.neuroimage.2013.05.114 23732883

[B239] Joshi J. Saharan S. Mandal P. K. (2014). BOLDSync: a MATLAB-based toolbox for synchronized stimulus presentation in functional MRI. J. Neurosci. Methods 223, 123–132. 10.1016/j.jneumeth.2013.12.002 24345673

[B240] Jovicich J. Minati L. Marizzoni M. Marchitelli R. Sala-Llonch R. Bartrés-Faz D.. (2016). Longitudinal reproducibility of default-mode network connectivity in healthy elderly participants: A multicentric resting-state fMRI study. Neuroimage 124, 442–454. 10.1016/j.neuroimage.2015.07.010 26163799

[B241] Joyce K. E. Hayasaka S. (2012). Development of PowerMap: a software package for statistical power calculation in neuroimaging studies. Neuroinformatics 10, 351–365. 10.1007/s12021-012-9152-3 22644868 PMC4426870

[B242] Just M. A. Cherkassky V. L. Keller T. A. Kana R. K. Minshew N. J. (2007). Functional and anatomical cortical underconnectivity in autism: evidence from an FMRI study of an executive function task and corpus callosum morphometry. Cereb. Cortex 17, 951–961. 10.1093/cercor/bhl006 16772313 PMC4500121

[B243] Jutten C. Herault J. (1991). Blind separation of sources, part I: an adaptive algorithm based on neuromimetic architecture. Signal Process. 24, 1–10. 10.1016/0165-1684(91)90079-X

[B244] Kadosh K. C. Luo Q. de Burca C. Sokunbi M. O. Feng J. Linden D. E. J.. (2016). Using real-time fMRI to influence effective connectivity in the developing emotion regulation network. Neuroimage 125, 616–626. 10.1016/j.neuroimage.2015.09.070 26475487 PMC4692450

[B245] Kaiser R. H. Whitfield-Gabrieli S. Dillon D. G. Goer F. Beltzer M. Minkel J.. (2016). Dynamic resting-state functional connectivity in major depression. Neuropsychopharmacology 41, 1822–1830. 10.1038/npp.2015.352 26632990 PMC4869051

[B246] Kalthoff D. Po C. Wiedermann D. Hoehn M. (2013). Reliability and spatial specificity of rat brain sensorimotor functional connectivity networks are superior under sedation compared with general anesthesia. NMR Biomed. 26, 638–650. 10.1002/nbm.2908 23303725

[B247] Kalthoff D. Seehafer J. U. Po C. Wiedermann D. Hoehn M. (2011). Functional connectivity in the rat at 11.7T: Impact of physiological noise in resting state fMRI. Neuroimage 54, 2828–2839. 10.1016/j.neuroimage.2010.10.053 20974263

[B248] Kasper L. Haeberlin M. Dietrich B. E. Gross S. Barmet C. Wilm B. J.. (2014). Matched-filter acquisition for BOLD fMRI. Neuroimage 100, 145–160. 10.1016/j.neuroimage.2014.05.024 24844745

[B249] Keator D. B. van Erp T. G. Turner J. A. Glover G. H. Mueller B. A. Liu T. T.. (2016). The function biomedical informatics research network data repository. Neuroimage 124, 1074–1079. 10.1016/j.neuroimage.2015.09.003 26364863 PMC4651841

[B250] Khan R. Zhang Q. Darayan S. Dhandapani S. Katyal S. Greene C.. (2011). Surface-based analysis methods for high-resolution functional magnetic resonance imaging. Graph. Models 73, 313–322. 10.1016/j.gmod.2010.11.002 22125419 PMC3223917

[B251] Kiebel S. Holmes A. (2003). The general linear model, in Human Brain Function, eds Frackowiak R. S. Friston K. J. Frith C. Dolan R. J. Price C. Zeki S. Ashburner J. Penny W. (Academic Press), 725–760.

[B252] Kiebel S. J. Klöppel S. Weiskopf N. Friston K. J. (2007). Dynamic causal modeling: a generative model of slice timing in fMRI. Neuroimage 34, 1487–1496. 10.1016/j.neuroimage.2006.10.026 17161624

[B253] Kiehl K. A. Smith A. M. Hare R. D. Mendrek A. Forster B. B. Brink J.. (2001). Limbic abnormalities in affective processing by criminal psychopaths as revealed by functional magnetic resonance imaging. Biol. Psychiatry 50, 677–684. 10.1016/S0006-3223(01)01222-7 11704074

[B254] Kiviniemi V. Kantola J. H. Jauhiainen J. Hyvärinen A. Tervonen O. (2003). Independent component analysis of nondeterministic fMRI signal sources. Neuroimage 19, 253–260. 10.1016/S1053-8119(03)00097-1 12814576

[B255] Klein A. Andersson J. Ardekani B. A. Ashburner J. Avants B. Chiang M. C.. (2009). Evaluation of 14 nonlinear deformation algorithms applied to human brain MRI registration. Neuroimage 46, 786–802. 10.1016/j.neuroimage.2008.12.037 19195496 PMC2747506

[B256] Klein A. Ghosh S. S. Avants B. Yeo B. T. Fischl B. Ardekani B.. (2010). Evaluation of volume-based and surface-based brain image registration methods. Neuroimage 51, 214–220. 10.1016/j.neuroimage.2010.01.091 20123029 PMC2862732

[B257] Kline R. B. (2011). Principles and Practice of Structural Equation Modeling. New York, NY: Guilford Press.

[B258] Knutson K. M. Wood J. N. Spampinato M. V. Grafman J. (2006). Politics on the brain: an FMRI investigation. Soc. Neurosci. 1, 25–40. 10.1080/17470910600670603 17372621 PMC1828689

[B259] Kober H. Lacadie C. M. Wexler B. E. Malison R. T. Sinha R. Potenza M. N. (2016). Brain activity during cocaine craving and gambling urges: an fMRI study. Neuropsychopharmacology 41, 628–637. 10.1038/npp.2015.193 26119472 PMC5130138

[B260] Koten J. W. Jr. Wood G. Hagoort P. Goebel R. Propping P. Willmes K.. (2009). Genetic contribution to variation in cognitive function: an FMRI study in twins. Science 323, 1737–1740. 10.1126/science.1167371 19325117

[B261] Kriegeskorte N. Cusack R. Bandettini P. (2010). How does an fMRI voxel sample the neuronal activity pattern: compact-kernel or complex spatiotemporal filter? Neuroimage 49, 1965–1976. 10.1016/j.neuroimage.2009.09.059 19800408 PMC2818340

[B262] Kriegeskorte N. Goebel R. Bandettini P. (2006). Information-based functional brain mapping. Proc. Natl. Acad. Sci. U.S.A. 103, 3863–3868. 10.1073/pnas.0600244103 16537458 PMC1383651

[B263] Kriegeskorte N. Simmons W. K. Bellgowan P. S. Baker C. I. (2009). Circular analysis in systems neuroscience: the dangers of double dipping. Nat. Neurosci. 12, 535–540. 10.1038/nn.2303 19396166 PMC2841687

[B264] Krishnadas R. Ryali S. Chen T. Uddin L. Supekar K. Palaniyappan L. (2014). Resting state functional hyperconnectivity within a triple network model in paranoid schizophrenia. Lancet 383, S65 10.1016/S0140-6736(14)60328-7

[B265] Kruggel F. von Cramon D. Y. Descombes X. (1999). Comparison of filtering methods for fMRI datasets. Neuroimage 10, 530–543. 10.1006/nimg.1999.0490 10547330

[B266] Kruschwitz J. D. List D. Waller L. Rubinov M. Walter H. (2015). GraphVar: a user-friendly toolbox for comprehensive graph analyses of functional brain connectivity. J. Neurosci. Methods 245, 107–115. 10.1016/j.jneumeth.2015.02.021 25725332

[B267] Kuhn S. Strelow E. Gallinat J. (2016). Multiple “buy buttons” in the brain: forecasting chocolate sales at point-of-sale based on functional brain activation using fMRI. Neuroimage 136, 122–128. 10.1016/j.neuroimage.2016.05.021 27173762

[B268] Kundu P. Brenowitz N. D. Voon V. Worbe Y. Vértes P. E. Inati S. J.. (2013). Integrated strategy for improving functional connectivity mapping using multiecho fMRI. Proc. Natl. Acad. Sci. U.S.A. 110, 16187–16192. 10.1073/pnas.1301725110 24038744 PMC3791700

[B269] Kwong K. K. (2012). Record of a single fMRI experiment in May of 1991. Neuroimage 62, 610–612. 10.1016/j.neuroimage.2011.07.089 21839841 PMC3236801

[B270] Kwong K. K. Belliveau J. W. Chesler D. A. Goldberg I. E. Weisskoff R. M. Poncelet B. P.. (1992). Dynamic magnetic resonance imaging of human brain activity during primary sensory stimulation. Proc. Natl. Acad. Sci. U.S.A. 89, 5675–5679. 1608978 10.1073/pnas.89.12.5675 PMC49355

[B271] Labudda K. Mertens M. Janszky J. Bien C. G. Woermann F. G. (2012). Atypical language lateralisation associated with right fronto-temporal grey matter increases–a combined fMRI and VBM study in left-sided mesial temporal lobe epilepsy patients. Neuroimage 59, 728–737. 10.1016/j.neuroimage.2011.07.053 21839176

[B272] Laird A. R. Robinson J. L. McMillan K. M. Tordesillas-Gutiérrez D. Moran S. T. Gonzales S. M.. (2010). Comparison of the disparity between Talairach and MNI coordinates in functional neuroimaging data: validation of the Lancaster transform. Neuroimage 51, 677–683. 10.1016/j.neuroimage.2010.02.048 20197097 PMC2856713

[B273] Lancaster J. L. Tordesillas-Gutiérrez D. Martinez M. Salinas F. Evans A. Zilles K.. (2007). Bias between MNI and Talairach coordinates analyzed using the ICBM-152 brain template. Hum. Brain Mapp. 28, 1194–1205. 10.1002/hbm.20345 17266101 PMC6871323

[B274] Lancaster J. L. Woldorff M. G. Parsons L. M. Liotti M. Freitas C. S. Rainey L.. (2000). Automated Talairach atlas labels for functional brain mapping. Hum. Brain Mapp. 10, 120–131. 10.1002/1097-0193(200007)10:3<120::AID-HBM30>3.0.CO;2-8 10912591 PMC6871915

[B275] Lang S. Duncan N. Northoff G. (2014). Resting-state functional magnetic resonance imaging: review of neurosurgical applications. Neurosurgery 74, 453–464; discussion: 464–455. 10.1227/neu.0000000000000307 24492661

[B276] Lange N. Strother S. C. Anderson J. R. Nielsen F. A. Holmes A. P. Kolenda T.. (1999). Plurality and resemblance in fMRI data analysis. Neuroimage 10, 282–303. 10.1006/nimg.1999.0472 10458943

[B277] Le Bihan D. (1996). Functional MRI of the brain principles, applications and limitations. J. Neuroradiol. 23, 1–5. 8767912

[B278] Le Bihan D. (2012). Diffusion, confusion and functional MRI. Neuroimage 62, 1131–1136. 10.1016/j.neuroimage.2011.09.058 21985905

[B279] Lee M. H. Hacker C. D. Snyder A. Z. Corbetta M. Zhang D. Leuthardt E. C.. (2012). Clustering of resting state networks. PLoS ONE 7:e40370. 10.1371/journal.pone.0040370 22792291 PMC3392237

[B280] Lee M. H. Miller-Thomas M. M. Benzinger T. L. Marcus D. S. Hacker C. D. Leuthardt E. C.. (2016). Clinical Resting-state fMRI in the Preoperative Setting: Are We Ready for Prime Time? Top. Magn. Reson. Imaging 25, 11–18. 10.1097/RMR.0000000000000075 26848556 PMC5640316

[B281] Lee M. H. Smyser C. D. Shimony J. S. (2013). Resting-state fMRI: a review of methods and clinical applications. AJNR Am. J. Neuroradiol. 34, 1866–1872. 10.3174/ajnr.A3263 22936095 PMC4035703

[B282] Leitão J. Thielscher A. Tünnerhoff J. Noppeney U. (2015). Concurrent TMS-fMRI reveals interactions between dorsal and ventral attentional systems. J. Neurosci. 35, 11445–11457. 10.1523/JNEUROSCI.0939-15.2015 26269649 PMC6605127

[B283] Leonardi N. Van De Ville D. (2015). On spurious and real fluctuations of dynamic functional connectivity during rest. Neuroimage 104, 430–436. 10.1016/j.neuroimage.2014.09.007 25234118

[B284] Levin J. M. Ross M. H. Mendelson J. H. Kaufman M. J. Lange N. Maas L. C.. (1998). Reduction in BOLD fMRI response to primary visual stimulation following alcohol ingestion. Psychiatry Res. 82, 135–146. 10.1016/S0925-4927(98)00022-5 9754438

[B285] Liang Z. King J. Zhang N. (2012). Anticorrelated resting-state functional connectivity in awake rat brain. Neuroimage 59, 1190–1199. 10.1016/j.neuroimage.2011.08.009 21864689 PMC3230741

[B286] Liao W. Deserno T. Spitzer K. (2008). Evaluation of Free Non-Diagnostic DICOM Software Tools. Bellingham; Washington, DC.

[B287] Liao W. Ding J. Marinazzo D. Xu Q. Wang Z. Yuan C.. (2011). Small-world directed networks in the human brain: multivariate Granger causality analysis of resting-state fMRI. Neuroimage 54, 2683–2694. 10.1016/j.neuroimage.2010.11.007 21073960

[B288] Liao W. Wu G. R. Xu Q. Ji G. J. Zhang Z. Zang Y. F.. (2014). DynamicBC: a MATLAB toolbox for dynamic brain connectome analysis. Brain Connect. 4, 780–790. 10.1089/brain.2014.0253 25083734 PMC4268585

[B289] Lieberman M. D. Cunningham W. A. (2009). Type I and Type II error concerns in fMRI research: re-balancing the scale. Soc. Cogn. Affect. Neurosci. 4, 423–428. 10.1093/scan/nsp052 20035017 PMC2799956

[B290] Lindquist M. A. (2008). The statistical analysis of fMRI data. Stat. Sci. 23, 439–464. 10.1214/09-sts282

[B291] Lindquist M. A. Zhang C.-H. Glover G. Shepp L. Yang Q. X. (2006). A generalization of the two-dimensional prolate spheroidal wave function method for nonrectilinear MRI data acquisition methods. IEEE Trans. Image Process. 15, 2792–2804. 10.1109/TIP.2006.877314 16948323

[B292] Liu S. Cai W. Liu S. Zhang F. Fulham M. Feng D.. (2015a). Multimodal neuroimaging computing: a review of the applications in neuropsychiatric disorders. Brain Inform. 2, 167–180. 10.1007/s40708-015-0019-x 27747507 PMC4737664

[B293] Liu S. Cai W. Liu S. Zhang F. Fulham M. Feng D.. (2015b). Multimodal neuroimaging computing: the workflows, methods, and platforms. Brain Inform. 2, 181–195. 10.1007/s40708-015-0020-4 27747508 PMC4737665

[B294] Liu T. T. (2004). Efficiency, power, and entropy in event-related fMRI with multiple trial types. Part II: design of experiments. Neuroimage 21, 401–413. 10.1016/j.neuroimage.2003.09.031 14741677

[B295] Liu T. T. (2012). The development of event-related fMRI designs. Neuroimage 62, 1157–1162. 10.1016/j.neuroimage.2011.10.008 22037002 PMC3272106

[B296] Liu T. T. (2013). Neurovascular factors in resting-state functional MRI. Neuroimage 80, 339–348. 10.1016/j.neuroimage.2013.04.071 23644003 PMC3746765

[B297] Liu X. Chang C. Duyn J. H. (2013). Decomposition of spontaneous brain activity into distinct fMRI co-activation patterns. Front. Syst. Neurosci. 7:101. 10.3389/fnsys.2013.00101 24550788 PMC3913885

[B298] Logothetis N. K. (2008). What we can do and what we cannot do with fMRI. Nature 453, 869–878. 10.1038/nature06976 18548064

[B299] Lohmann G. Stelzer J. Neumann J. Ay N. Turner R. (2013). "More is different" in functional magnetic resonance imaging: a review of recent data analysis techniques. Brain Connect. 3, 223–239. 10.1089/brain.2012.0133 23402339

[B300] Long X. Y. Zuo X. N. Kiviniemi V. Yang Y. Zou Q. H. Zhu C. Z.. (2008). Default mode network as revealed with multiple methods for resting-state functional MRI analysis. J. Neurosci. Methods 171, 349–355. 10.1016/j.jneumeth.2008.03.021 18486233

[B301] Lu H. Golay X. Pekar J. J. Van Zijl P. C. (2003). Functional magnetic resonance imaging based on changes in vascular space occupancy. Magn. Reson. Med. 50, 263–274. 10.1002/mrm.10519 12876702

[B302] Lu H. van Zijl P. C. (2012). A review of the development of Vascular-Space-Occupancy (VASO) fMRI. Neuroimage 62, 736–742. 10.1016/j.neuroimage.2012.01.013 22245650 PMC3328630

[B303] Lund T. E. Madsen K. H. Sidaros K. Luo W.-L. Nichols T. E. (2006). Non-white noise in fMRI: Does modelling have an impact? Neuroimage 29, 54–66. 10.1016/j.neuroimage.2005.07.005 16099175

[B304] Machulda M. M. Ward H. A. Borowski B. Gunter J. L. Cha R. H. O'Brien P. C.. (2003). Comparison of memory fMRI response among normal, MCI, and Alzheimer's patients. Neurology 61, 500–506. 10.1212/01.WNL.0000079052.01016.78 12939424 PMC2744465

[B305] Maclaren J. Herbst M. Speck O. Zaitsev M. (2013). Prospective motion correction in brain imaging: a review. Magn. Reson. Med. 69, 621–636. 10.1002/mrm.24314 22570274

[B306] Madhyastha T. M. Askren M. K. Boord P. Grabowski T. J. (2015). Dynamic connectivity at rest predicts attention task performance. Brain Connect. 5, 45–59. 10.1089/brain.2014.0248 25014419 PMC4313397

[B307] Magalhães R. Marques P. Soares J. Alves V. Sousa N. (2015). The impact of normalization and segmentation on resting-state brain networks. Brain Connect. 5, 166–176. 10.1089/brain.2014.0292 25420048

[B308] Mahmoudi A. Takerkart S. Regragui F. Boussaoud D. Brovelli A. (2012). Multivoxel pattern analysis for FMRI data: a review. Comput. Math. Methods Med. 2012:961257. 10.1155/2012/961257 23401720 PMC3529504

[B309] Mandelkow H. de Zwart J. Duyn J. (2016). Linear Discriminant analysis achieves high classification accuracy for the BOLD fMRI response to naturalistic movie stimuli. Front. Hum. Neurosci. 10:128. 10.3389/fnhum.2016.00128 27065832 PMC4815557

[B310] Månsson K. N. Frick A. Boraxbekk C.-J. Marquand A. Williams S. C. Carlbring P.. (2015). Predicting long-term outcome of Internet-delivered cognitive behavior therapy for social anxiety disorder using fMRI and support vector machine learning. Transl. Psychiatry 5, e530. 10.1038/tp.2015.22 25781229 PMC4354352

[B311] Margulies D. S. Böttger J. Long X. Lv Y. Kelly C. Schäfer A.. (2010). Resting developments: a review of fMRI post-processing methodologies for spontaneous brain activity. MAGMA 23, 289–307. 10.1007/s10334-010-0228-5 20972883

[B312] Markett S. Reuter M. Montag C. Voigt G. Lachmann B. Rudorf S.. (2014). Assessing the function of the fronto-parietal attention network: insights from resting-state fMRI and the attentional network test. Hum. Brain Mapp. 35, 1700–1709. 10.1002/hbm.22285 23670989 PMC6869384

[B313] Marques P. C. G. Soares J. M. Alves V. Sousa N. (2013). BrainCAT - a tool for automated and combined functional Magnetic Resonance Imaging and Diffusion Tensor Imaging brain connectivity analysis. Front. Hum. Neurosci. 7:794. 10.3389/fnhum.2013.00794 24319419 PMC3836207

[B314] Marques P. Moreira P. Magalhães R. Costa P. Santos N. Zihl J.. (2016). The functional connectome of cognitive reserve. Hum. Brain Mapp. 37, 3310–3322. 10.1002/hbm.23242 27144904 PMC5084807

[B315] Matthews P. M. Honey G. D. Bullmore E. T. (2006). Applications of fMRI in translational medicine and clinical practice. Nat. Rev. Neurosci. 7, 732–744. 10.1038/nrn1929 16924262

[B316] Maus B. van Breukelen G. J. Goebel R. Berger M. P. (2010). Robustness of optimal design of fMRI experiments with application of a genetic algorithm. Neuroimage 49, 2433–2443. 10.1016/j.neuroimage.2009.10.004 19833212

[B317] Maus B. van Breukelen G. J. P. (2013). Optimal design for functional magnetic resonance imaging experiments: Methodology, challenges, and future perspectives. Zeitschrift Psychologie 221, 174–189. 10.1027/2151-2604/a000145

[B318] Mazaika P. Whitfield-Gabrieli S. Reiss A. Glover G. (2007). Artifact repair for fMRI data from high motion clinical subjects, in 13th Annual Meeting of the Organization for Human Brain Mapping (Chicago: IL).

[B319] Mazziotta J. C. Toga A. W. Evans A. Fox P. Lancaster J. (1995). A probabilistic atlas of the human brain: theory and rationale for its development. The International Consortium for Brain Mapping (ICBM). Neuroimage 2, 89–101. 10.1006/nimg.1995.1012 9343592

[B320] Mazziotta J. Toga A. Evans A. Fox P. Lancaster J. Zilles K.. (2001). A probabilistic atlas and reference system for the human brain: International Consortium for Brain Mapping (ICBM). Philos. Trans. R. Soc. Lond. B Biol. Sci. 356, 1293–1322. 10.1098/rstb.2001.0915 11545704 PMC1088516

[B321] McFarquhar M. McKie S. Emsley R. Suckling J. Elliott R. Williams S. (2016). Multivariate and repeated measures (MRM): A new toolbox for dependent and multimodal group-level neuroimaging data. Neuroimage 132, 373–389. 10.1016/j.neuroimage.2016.02.053 26921716 PMC4862963

[B322] McGonigle D. J. (2012). Test–retest reliability in fMRI: Or how I learned to stop worrying and love the variability. Neuroimage 62, 1116–1120. 10.1016/j.neuroimage.2012.01.023 22261373

[B323] McLaren D. G. Ries M. L. Xu G. Johnson S. C. (2012). A generalized form of context-dependent psychophysiological interactions (gPPI): a comparison to standard approaches. Neuroimage 61, 1277–1286. 10.1016/j.neuroimage.2012.03.068 22484411 PMC3376181

[B324] McLntosh A. R. Gonzalez-Lima F. (1994). Structural equation modeling and its application to network analysis in functional brain imaging. Hum. Brain Mapp. 2, 2–22. 10.1002/hbm.460020104

[B325] Meade C. S. Cordero D. M. Hobkirk A. L. Metra B. M. Chen N.-K. Huettel S. A. (2016). Compensatory activation in fronto-parietal cortices among HIV-infected persons during a monetary decision-making task. Hum. Brain Mapp. 37, 2455–2467. 10.1002/hbm.23185 27004729 PMC4913278

[B326] Meier T. B. Desphande A. S. Vergun S. Nair V. A. Song J. Biswal B. B.. (2012). Support vector machine classification and characterization of age-related reorganization of functional brain networks. Neuroimage 60, 601–613. 10.1016/j.neuroimage.2011.12.052 22227886 PMC3288439

[B327] Meyer M. L. Taylor S. E. Lieberman M. D. (2015). Social working memory and its distinctive link to social cognitive ability: an fMRI study. Soc. Cogn. Affect. Neurosci. 10, 1338–1347. 10.1093/scan/nsv065 25987597 PMC4590544

[B328] Meyer T. Constantinidis C. (2005). A software solution for the control of visual behavioral experimentation. J. Neurosci. Methods 142, 27–34. 10.1016/j.jneumeth.2004.07.009 15652614

[B329] Meyer-Baese A. Wismueller A. Lange O. (2004). Comparison of two exploratory data analysis methods for fMRI: unsupervised clustering versus independent component analysis. IEEE Trans. Inf. Technol. Biomed. 8, 387–398. 10.1109/TITB.2004.834406 15484444

[B330] Mezer A. Yovel Y. Pasternak O. Gorfine T. Assaf Y. (2009). Cluster analysis of resting-state fMRI time series. Neuroimage 45, 1117–1125. 10.1016/j.neuroimage.2008.12.015 19146962

[B331] Miezin F. M. Maccotta L. Ollinger J. M. Petersen S. E. Buckner R. L. (2000). Characterizing the hemodynamic response: effects of presentation rate, sampling procedure, and the possibility of ordering brain activity based on relative timing. Neuroimage 11, 735–759. 10.1006/nimg.2000.0568 10860799

[B332] Mikl M. Marecek R. Hlustík P. Pavlicová M. Drastich A. Chlebus P.. (2008). Effects of spatial smoothing on fMRI group inferences. Magn. Reson. Imaging 26, 490–503. 10.1016/j.mri.2007.08.006 18060720

[B333] Mildenberger P. Eichelberg M. Martin E. (2002). Introduction to the DICOM standard. Eur. Radiol. 12, 920–927. 10.1007/s003300101100 11960249

[B334] Millman K. J. Brett M. (2007). Analysis of Functional Magnetic Resonance Imaging in Python. Comput. Sci. Eng. 9, 52–55. 10.1109/MCSE.2007.46

[B335] Molloy E. K. Meyerand M. E. Birn R. M. (2014). The influence of spatial resolution and smoothing on the detectability of resting-state and task fMRI. Neuroimage 86, 221–230. 10.1016/j.neuroimage.2013.09.001 24021836 PMC5736131

[B336] Monti M. M. (2011). Statistical Analysis of fMRI Time-Series: A Critical Review of the GLM Approach. Front. Hum. Neurosci. 5:28. 10.3389/fnhum.2011.00028 21442013 PMC3062970

[B337] Morgan V. L. Dawant B. M. Li Y. Pickens D. R. (2007). Comparison of fMRI statistical software packages and strategies for analysis of images containing random and stimulus-correlated motion. Comput. Med. Imaging Graph. 31, 436–446. 10.1016/j.compmedimag.2007.04.002 17574816 PMC2570159

[B338] Mowinckel A. M. Espeseth T. Westlye L. T. (2012). Network-specific effects of age and in-scanner subject motion: a resting-state fMRI study of 238 healthy adults. Neuroimage 63, 1364–1373. 10.1016/j.neuroimage.2012.08.004 22992492

[B339] Mulderink T. A. Gitelman D. R. Mesulam M. M. Parrish T. B. (2002). On the Use of Caffeine as a Contrast Booster for BOLD fMRI Studies. Neuroimage 15, 37–44. 10.1006/nimg.2001.0973 11771972

[B340] Mumford J. A. (2012). A power calculation guide for fMRI studies. Soc. Cogn. Affect. Neurosci. 7, 738–742. 10.1093/scan/nss059 22641837 PMC3427872

[B341] Mumford J. A. Nichols T. E. (2008). Power calculation for group fMRI studies accounting for arbitrary design and temporal autocorrelation. Neuroimage 39, 261–268. 10.1016/j.neuroimage.2007.07.061 17919925 PMC2423281

[B342] Muresan L. Renken R. Roerdink J. B. Duifhuis H. (2005). Automated correction of spin-history related motion artefacts in fMRI: simulated and phantom data. IEEE Trans. Biomed. Eng. 52, 1450–1460. 10.1109/TBME.2005.851484 16119241

[B343] Murphy K. Birn R. M. Bandettini P. A. (2013). Resting-state fMRI confounds and cleanup. Neuroimage 80, 349–359. 10.1016/j.neuroimage.2013.04.001 23571418 PMC3720818

[B344] Murphy K. Garavan H. (2004). An empirical investigation into the number of subjects required for an event-related fMRI study. Neuroimage 22, 879–885. 10.1016/j.neuroimage.2004.02.005 15193618

[B345] Mustra M. Delac K. Grgic M. (2008). Overview of the DICOM standard, in 50th International Symposium (Zadar: ELMAR), 39–44.

[B346] Ngan S.-C. Laconte S. M. Hu X. (2000). Temporal Filtering of event-related fMRI data using cross-validation. Neuroimage 11, 797–804. 10.1006/nimg.2000.0558 10860803

[B347] Nichols T. E. Das S. Eickhoff S. B. Evans A. C. Glatard T. Hanke M. (2016). Best practices in data analysis and sharing in neuroimaging using MRI. bioRxiv. 10.1038/nn.4500 PMC5685169 28230846

[B348] Nichols T. E. Holmes A. P. (2002). Nonparametric permutation tests for functional neuroimaging: a primer with examples. Hum. Brain Mapp. 15, 1–25. 10.1002/hbm.1058 11747097 PMC6871862

[B349] Nichols T. Hayasaka S. (2003). Controlling the familywise error rate in functional neuroimaging: a comparative review. Stat. Methods Med. Res. 12, 419–446. 10.1191/0962280203sm341ra 14599004

[B350] Nickl-Jockschat T. Rottschy C. Thommes J. Schneider F. Laird A. R. Fox P. T.. (2015). Neural networks related to dysfunctional face processing in autism spectrum disorder. Brain Struct. Funct. 220, 2355–2371. 10.1007/s00429-014-0791-z 24869925 PMC4782795

[B351] Nie B. Chen K. Zhao S. Liu J. Gu X. Yao Q.. (2013). A rat brain MRI template with digital stereotaxic atlas of fine anatomical delineations in paxinos space and its automated application in voxel-wise analysis. Hum. Brain Mapp. 34, 1306–1318. 10.1002/hbm.21511 22287270 PMC4110061

[B352] Nomi J. S. Scherfeld D. Friederichs S. Schäfer R. Franz M. Wittsack H. J.. (2008). On the neural networks of empathy: a principal component analysis of an fMRI study. Behav. Brain Funct. 4:41. 10.1186/1744-9081-4-41 18798977 PMC2564949

[B353] Norman K. A. Polyn S. M. Detre G. J. Haxby J. V. (2006). Beyond mind-reading: multi-voxel pattern analysis of fMRI data. Trends Cogn. Sci. 10, 424–430. 10.1016/j.tics.2006.07.005 16899397

[B354] Norris D. G. (2006). Principles of magnetic resonance assessment of brain function. J. Magn. Reson. Imaging 23, 794–807. 10.1002/jmri.20587 16649206

[B355] Norris D. G. (2012). Spin-echo fMRI: the poor relation? Neuroimage 62, 1109–1115. 10.1016/j.neuroimage.2012.01.003 22245351

[B356] Oakes T. R. Johnstone T. Ores Walsh K. S. Greischar L. L. Alexander A. L. Fox A. S.. (2005). Comparison of fMRI motion correction software tools. Neuroimage 28, 529–543. 10.1016/j.neuroimage.2005.05.058 16099178

[B357] Ogawa S. (2012). Finding the BOLD effect in brain images. Neuroimage 62, 608–609. 10.1016/j.neuroimage.2012.01.091 22309802

[B358] Ogawa S. Lee T. M. (1990). Magnetic resonance imaging of blood vessels at high fields: *in vivo* and *in vitro* measurements and image simulation. Magn. Reson. Med. 16, 9–18. 10.1002/mrm.1910160103 2255240

[B359] Ogawa S. Lee T. M. Kay A. R. Tank D. W. (1990a). Brain magnetic resonance imaging with contrast dependent on blood oxygenation. Proc. Natl. Acad. Sci. U.S.A. 87, 9868–9872. 2124706 10.1073/pnas.87.24.9868 PMC55275

[B360] Ogawa S. Lee T. M. Nayak A. S. Glynn P. (1990b). Oxygenation-sensitive contrast in magnetic resonance image of rodent brain at high magnetic fields. Magn. Reson. Med. 14, 68–78. 2161986 10.1002/mrm.1910140108

[B361] Ogawa S. Tank D. W. Menon R. Ellermann J. M. Kim S. G. Merkle H.. (1992). Intrinsic signal changes accompanying sensory stimulation: functional brain mapping with magnetic resonance imaging. Proc. Natl. Acad. Sci. U.S.A. 89, 5951–5955. 10.1073/pnas.89.13.5951 1631079 PMC402116

[B362] Olman C. A. Yacoub E. (2011). High-field FMRI for human applications: an overview of spatial resolution and signal specificity. Open Neuroimag. J. 5, 74–89. 10.2174/1874440001105010074 22216080 PMC3245408

[B363] O'Reilly J. X. Woolrich M. W. Behrens T. E. Smith S. M. Johansen-Berg H. (2012). Tools of the trade: psychophysiological interactions and functional connectivity. Soc. Cogn. Affect. Neurosci. 7, 604–609. 10.1093/scan/nss055 22569188 PMC3375893

[B364] Pan W. J. Thompson G. J. Magnuson M. E. Jaeger D. Keilholz S. (2013). Infraslow LFP correlates to resting-state fMRI BOLD signals. Neuroimage 74, 288–297. 10.1016/j.neuroimage.2013.02.035 23481462 PMC3615090

[B365] Papademetris X. Jackowski M. P. Rajeevan N. Distasio M. Okuda H. Constable R. T.. (2006). BioImage suite: an integrated medical image analysis suite: an update. Insight J. 2006:209. 25364771 PMC4213804

[B366] Papp E. A. Leergaard T. B. Calabrese E. Johnson G. A. Bjaalie J. G. (2014). Waxholm space atlas of the sprague dawley rat brain. Neuroimage 97, 374–386. 10.1016/j.neuroimage.2014.04.001 24726336 PMC4160085

[B367] Patriat R. Molloy E. K. Meier T. B. Kirk G. R. Nair V. A. Meyerand M. E.. (2013). The effect of resting condition on resting-state fMRI reliability and consistency: a comparison between resting with eyes open, closed, and fixated. Neuroimage 78, 463–473. 10.1016/j.neuroimage.2013.04.013 23597935 PMC4003890

[B368] Pauli R. Bowring A. Reynolds R. Chen G. Nichols T. E. Maumet C. (2016). Exploring fMRI results space: 31 variants of an fMRI analysis in AFNI, FSL, and SPM. Front. Neuroinform. 10:24. 10.3389/fninf.2016.00024 27458367 PMC4932120

[B369] Peck K. K. Wierenga C. E. Moore A. B. Maher L. M. Gopinath K. Gaiefsky M.. (2004). Comparison of baseline conditions to investigate syntactic production using functional magnetic resonance imaging. Neuroimage 23, 104–110. 10.1016/j.neuroimage.2004.05.006 15325357

[B370] Pedersen M. Curwood E. K. Archer J. S. Abbott D. F. Jackson G. D. (2015). Brain regions with abnormal network properties in severe epilepsy of Lennox-Gastaut phenotype: Multivariate analysis of task-free fMRI. Epilepsia 56, 1767–1773. 10.1111/epi.13135 26333833

[B371] Peirce J. W. (2008). Generating stimuli for neuroscience using PsychoPy. Front. Neuroinform. 2:10. 10.3389/neuro.11.010.2008 19198666 PMC2636899

[B372] Penny W. D. Stephan K. E. Mechelli A. Friston K. (2004). Comparing dynamic causal models. Neuroimage 22, 1157–1172. 10.1016/j.neuroimage.2004.03.026 15219588

[B373] Pereira F. Mitchell T. Botvinick M. (2009). Machine learning classifiers and fMRI: a tutorial overview. Neuroimage 45, S199–209. 10.1016/j.neuroimage.2008.11.007 19070668 PMC2892746

[B374] Perlbarg V. Bellec P. Anton J. L. Pélégrini-Issac M. Doyon J. Benali H. (2007). CORSICA: correction of structured noise in fMRI by automatic identification of ICA components. Magn. Reson. Imaging 25, 35–46. 10.1016/j.mri.2006.09.042 17222713

[B375] Pernet C. R. Gorgolewski K. J. Job D. Rodriguez D. Whittle I. Wardlaw J. (2016). A structural and functional magnetic resonance imaging dataset of brain tumour patients. Sci. Data 3:160003. 10.1038/sdata.2016.3 26836205 PMC4736501

[B376] Perthen J. E. Lansing A. E. Liau J. Liu T. T. Buxton R. B. (2008). Caffeine-induced uncoupling of cerebral blood flow and oxygen metabolism: a calibrated BOLD fMRI study. Neuroimage 40, 237–247. 10.1016/j.neuroimage.2007.10.049 18191583 PMC2716699

[B377] Peters J. C. Reithler J. Schuhmann T. de Graaf T. Uludag K. Goebel R.. (2013). On the feasibility of concurrent human TMS-EEG-fMRI measurements. J. Neurophysiol. 109, 1214–1227. 10.1152/jn.00071.2012 23221407 PMC3569123

[B378] Petersen S. E. Dubis J. W. (2012). The mixed block/event-related design. Neuroimage 62, 1177–1184. 10.1016/j.neuroimage.2011.09.084 22008373 PMC3288695

[B379] Petkov C. I. Kikuchi Y. Milne A. E. Mishkin M. Rauschecker J. P. Logothetis N. K. (2015). Different forms of effective connectivity in primate frontotemporal pathways. Nat. Commun. 6, 6000. 10.1038/ncomms7000 25613079 PMC4306228

[B380] Phillips A. A. Chan F. H. Zheng M. M. Krassioukov A. V. Ainslie P. N. (2016). Neurovascular coupling in humans*:* Physiology, methodological advances and clinical implications. J. Cereb. Blood Flow Metab. 36, 647–664. 10.1177/0271678X15617954 26661243 PMC4821024

[B381] Poldrack R. A. (2000). Imaging brain plasticity: conceptual and methodological issues–a theoretical review. Neuroimage 12, 1–13. 10.1006/nimg.2000.0596 10875897

[B382] Poldrack R. A. (2006). Can cognitive processes be inferred from neuroimaging data? Trends Cogn. Sci. 10, 59–63. 10.1016/j.tics.2005.12.004 16406760

[B383] Poldrack R. A. (2007). Region of interest analysis for fMRI. Soc. Cogn. Affect. Neurosci. 2, 67–70. 10.1093/scan/nsm006 18985121 PMC2555436

[B384] Poldrack R. A. (2008). The role of fMRI in cognitive neuroscience: where do we stand? Curr. Opin. Neurobiol. 18, 223–227. 10.1016/j.conb.2008.07.006 18678252

[B385] Poldrack R. A. (2012). The future of fMRI in cognitive neuroscience. Neuroimage 62, 1216–1220. 10.1016/j.neuroimage.2011.08.007 21856431 PMC4131441

[B386] Poldrack R. A. Fletcher P. C. Henson R. N. Worsley K. J. Brett M. Nichols T. E. (2008). Guidelines for reporting an fMRI study. Neuroimage 40, 409–414. 10.1016/j.neuroimage.2007.11.048 18191585 PMC2287206

[B387] Poldrack R. A. Gorgolewski K. J. (2014). Making big data open: data sharing in neuroimaging. Nat. Neurosci. 17, 1510–1517. 10.1038/nn.3818 25349916

[B388] Poldrack R. A. Gorgolewski K. J. (2015). OpenfMRI: open sharing of task fMRI data. Neuroimage. 10.1016/j.neuroimage.2015.05.073. [Epub ahead of print]. 26048618 PMC4669234

[B389] Poldrack R. A. Halchenko Y. O. Hanson S. J. (2009). Decoding the large-scale structure of brain function by classifying mental States across individuals. Psychol. Sci. 20, 1364–1372. 10.1111/j.1467-9280.2009.02460.x 19883493 PMC2935493

[B390] Poldrack R. A. Mumford J. A. Nichols T. E. (2011). Handbook of Functional MRI Data Analysis. New York, NY: Cambridge University Press 10.1017/cbo9780511895029

[B391] Poldrack R. A. Poline J. B. (2015). The publication and reproducibility challenges of shared data. Trends Cogn. Sci. 19, 59–61. 10.1016/j.tics.2014.11.008 25532702

[B392] Poldrack R. A. Yarkoni T. (2016). From brain maps to cognitive ontologies: informatics and the search for mental structure. Annu. Rev. Psychol. 67, 587–612. 10.1146/annurev-psych-122414-033729 26393866 PMC4701616

[B393] Poline J.-B. Brett M. (2012). The general linear model and fMRI: does love last forever? Neuroimage 62, 871–880. 10.1016/j.neuroimage.2012.01.133 22343127

[B394] Polyn S. M. Natu V. S. Cohen J. D. Norman K. A. (2005). Category-specific cortical activity precedes retrieval during memory search. Science 310, 1963–1966. 10.1126/science.1117645 16373577

[B395] Poustchi-Amin M. Mirowitz S. A. Brown J. J. McKinstry R. C. Li T. (2001). Principles and applications of echo-planar imaging: a review for the general radiologist. Radiographics 21, 767–779. 10.1148/radiographics.21.3.g01ma23767 11353123

[B396] Power J. D. Barnes K. A. Snyder A. Z. Schlaggar B. L. Petersen S. E. (2012). Spurious but systematic correlations in functional connectivity MRI networks arise from subject motion. Neuroimage 59, 2142–2154. 10.1016/j.neuroimage.2011.10.018 22019881 PMC3254728

[B397] Power J. D. Mitra A. Laumann T. O. Snyder A. Z. Schlaggar B. L. Petersen S. E. (2014). Methods to detect, characterize, and remove motion artifact in resting state fMRI. Neuroimage 84, 320–341. 10.1016/j.neuroimage.2013.08.048 23994314 PMC3849338

[B398] Power J. D. Schlaggar B. L. Petersen S. E. (2015). Recent progress and outstanding issues in motion correction in resting state fMRI. Neuroimage 105, 536–551. 10.1016/j.neuroimage.2014.10.044 25462692 PMC4262543

[B399] Preibisch C. Castrillón G. J. Buhrer M. Riedl V. (2015). Evaluation of Multiband EPI Acquisitions for Resting State fMRI. PLoS ONE 10:e0136961. 10.1371/journal.pone.0136961 26375666 PMC4574400

[B400] Premi E. Cauda F. Costa T. Diano M. Gazzina S. Gualeni V.. (2016). Looking for neuroimaging markers in frontotemporal lobar degeneration clinical trials: a multi-voxel pattern analysis study in granulin disease. J. Alzheimers Dis. 51, 249–262. 10.3233/JAD-150340 26836150

[B401] Price C. J. (2012). A review and synthesis of the first 20 years of PET and fMRI studies of heard speech, spoken language and reading. Neuroimage 62, 816–847. 10.1016/j.neuroimage.2012.04.062 22584224 PMC3398395

[B402] Price C. J. Moore C. J. Friston K. J. (1997). Subtractions, conjunctions, and interactions in experimental design of activation studies. Hum. Brain Mapp. 5, 264–272. 20408227 10.1002/(SICI)1097-0193(1997)5:4<264::AID-HBM11>3.0.CO;2-E

[B403] Pruessmann K. P. Weiger M. Scheidegger M. B. Boesiger P. (1999). SENSE: sensitivity encoding for fast MRI. Magn. Reson. Med. 42, 952–962. 10542355

[B404] Pruim R. H. Mennes M. van Rooij D. Llera A. Buitelaar J. K. Beckmann C. F. (2015). ICA-AROMA: A robust ICA-based strategy for removing motion artifacts from fMRI data. Neuroimage 112, 267–277. 10.1016/j.neuroimage.2015.02.064 25770991

[B405] Radua J. Mataix-Cols D. (2012). Meta-analytic methods for neuroimaging data explained. Biol. Mood Anxiety Disord. 2:6. 10.1186/2045-5380-2-6 22737993 PMC3384225

[B406] Radua J. Mataix-Cols D. Phillips M. L. El-Hage W. Kronhaus D. M. Cardoner N.. (2012). A new meta-analytic method for neuroimaging studies that combines reported peak coordinates and statistical parametric maps. Eur. Psychiatry 27, 605–611. 10.1016/j.eurpsy.2011.04.001 21658917

[B407] Raichle M. E. (2001). Cognitive neuroscience. Bold insights. Nature 412, 128–130. 10.1038/35084300 11449247

[B408] Raichle M. E. (2009). A paradigm shift in functional brain imaging. J. Neurosci. 29, 12729–12734. 10.1523/JNEUROSCI.4366-09.2009 19828783 PMC6665302

[B409] Rao C. Singh N. C. (2015). Visuospatial complexity modulates reading in the brain. Brain Lang. 141, 50–61. 10.1016/j.bandl.2014.11.010 25528288

[B410] Razi A. Kahan J. Rees G. Friston K. J. (2015). Construct validation of a DCM for resting state fMRI. Neuroimage 106, 1–14. 10.1016/j.neuroimage.2014.11.027 25463471 PMC4295921

[B411] Reese T. G. Davis T. L. Weisskoff R. M. (1995). Automated shimming at 1.5 T using echo-planar image frequency maps. J. Magn. Reson. Imaging 5, 739–745. 10.1002/jmri.1880050621 8748496

[B412] Reijneveld J. C. Ponten S. C. Berendse H. W. Stam C. J. (2007). The application of graph theoretical analysis to complex networks in the brain. Clin. Neurophysiol. 118, 2317–2331. 10.1016/j.clinph.2007.08.010 17900977

[B413] Reimold M. Slifstein M. Heinz A. Mueller-Schauenburg W. Bares R. (2006). Effect of spatial smoothing on t-maps: arguments for going back from t-maps to masked contrast images. J. Cereb. Blood Flow Metab. 26, 751–759. 10.1038/sj.jcbfm.9600231 16208316

[B414] Renvall V. Nangini C. Hari R. (2014). All that glitters is not BOLD: inconsistencies in functional MRI. Sci. Rep. 4, 3920. 10.1038/srep03920 24472878 PMC3905278

[B415] Rex D. E. Ma J. Q. Toga A. W. (2003). The LONI pipeline processing environment. Neuroimage 19, 1033–1048. 10.1016/S1053-8119(03)00185-X 12880830

[B416] Richiardi J. Altmann A. Milazzo A. C. Chang C. Chakravarty M. M. Banaschewski T.. (2015). BRAIN NETWORKS. Correlated gene expression supports synchronous activity in brain networks. Science 348, 1241–1244. 10.1126/science.1255905 26068849 PMC4829082

[B417] Rigoux L. Daunizeau J. (2015). Dynamic causal modelling of brain-behaviour relationships. Neuroimage 117, 202–221. 10.1016/j.neuroimage.2015.05.041 26008885

[B418] Robinson S. D. Schöpf V. Cardoso P. Geissler A. Fischmeister F. P. Wurnig M.. (2013). Applying independent component analysis to clinical FMRI at 7 t. Front. Hum. Neurosci. 7:496. 10.3389/fnhum.2013.00496 24032007 PMC3759034

[B419] Roche A. (2011). A four-dimensional registration algorithm with application to joint correction of motion and slice timing in fMRI. IEEE Trans. Med. Imaging 30, 1546–1554. 10.1109/TMI.2011.2131152 21427017

[B420] Rosazza C. Minati L. (2011). Resting-state brain networks: literature review and clinical applications. Neurol. Sci. 32, 773–785. 10.1007/s10072-011-0636-y 21667095

[B421] Rosset A. Spadola L. Ratib O. (2004). OsiriX: an open-source software for navigating in multidimensional DICOM images. J. Digit. Imaging 17, 205–216. 10.1007/s10278-004-1014-6 15534753 PMC3046608

[B422] Rubinov M. Sporns O. (2010). Complex network measures of brain connectivity: uses and interpretations. Neuroimage 52, 1059–1069. 10.1016/j.neuroimage.2009.10.003 19819337

[B423] Ruff C. C. Driver J. Bestmann S. (2009). Combining TMS and fMRI: from 'virtual lesions' to functional-network accounts of cognition. Cortex 45, 1043–1049. 10.1016/j.cortex.2008.10.012 19166996 PMC2726131

[B424] Rydell J. Knutsson H. Borga M. (2008). Bilateral Filtering of fMRI Data. IEEE J. Sel. Top. Signal Process. 2, 891–896. 10.1109/JSTSP.2008.2007826

[B425] Sacchet M. D. Knutson B. (2013). Spatial smoothing systematically biases the localization of reward-related brain activity. Neuroimage 66, 270–277. 10.1016/j.neuroimage.2012.10.056 23110886 PMC3618861

[B426] Sack A. T. Camprodon J. A. Pascual-Leone A. Goebel R. (2005). The dynamics of interhemispheric compensatory processes in mental imagery. Science 308, 702–704. 10.1126/science.1107784 15860630

[B427] Sampaio A. Soares J. M. Coutinho J. Sousa N. Gonçalves Ó. F. (2014). The big five default brain: functional evidence. Brain Struct. Funct. 219, 1913–1922. 10.1007/s00429-013-0610-y 23881294

[B428] Sander C. Y. Hooker J. M. Catana C. Normandin M. D. Alpert N. M. Knudsen G. M.. (2013). Neurovascular coupling to D2/D3 dopamine receptor occupancy using simultaneous PET/functional MRI. Proc. Natl. Acad. Sci. U.S.A. 110, 11169–11174. 10.1073/pnas.1220512110 23723346 PMC3703969

[B429] Särkkä S. Solin A. Nummenmaa A. Vehtari A. Auranen T. Vanni S.. (2012). Dynamic retrospective filtering of physiological noise in BOLD fMRI: DRIFTER. Neuroimage 60, 1517–1527. 10.1016/j.neuroimage.2012.01.067 22281675 PMC3303954

[B430] Sato H. Yahata N. Funane T. Takizawa R. Katura T. Atsumori H.. (2013). A NIRS-fMRI investigation of prefrontal cortex activity during a working memory task. Neuroimage 83, 158–173. 10.1016/j.neuroimage.2013.06.043 23792984

[B431] Satterthwaite T. D. Wolf D. H. Loughead J. Ruparel K. Elliott M. A. Hakonarson H.. (2012). Impact of in-scanner head motion on multiple measures of functional connectivity: relevance for studies of neurodevelopment in youth. Neuroimage 60, 623–632. 10.1016/j.neuroimage.2011.12.063 22233733 PMC3746318

[B432] Scheinost D. Papademetris X. Constable R. T. (2014). The impact of image smoothness on intrinsic functional connectivity and head motion confounds. Neuroimage 95, 13–21. 10.1016/j.neuroimage.2014.03.035 24657356 PMC4076413

[B433] Scheinost D. Stoica T. Saksa J. Papademetris X. Constable R. T. Pittenger C.. (2013). Orbitofrontal cortex neurofeedback produces lasting changes in contamination anxiety and resting-state connectivity. Transl. Psychiatry 3, e250. 10.1038/tp.2013.24 23632454 PMC3641411

[B434] Schlegel F. Schroeter A. Rudin M. (2015). The hemodynamic response to somatosensory stimulation in mice depends on the anesthetic used: implications on analysis of mouse fMRI data. Neuroimage 116, 40–49. 10.1016/j.neuroimage.2015.05.013 25979665

[B435] Schmithorst V. J. Dardzinski B. J. Holland S. K. (2001). Simultaneous correction of ghost and geometric distortion artifacts in EPI using a multiecho reference scan. IEEE Trans. Med. Imaging 20, 535–539. 10.1109/42.929619 11437113 PMC1357361

[B436] Schmitt F. Mansfield P. Stehling M. K. Turner R. (2012). Echo-Planar Imaging: Theory, Technique and Application. Heidelberg: Springer.

[B437] Schölvinck M. L. Maier A. Ye F. Q. Duyn J. H. Leopold D. A. (2010). Neural basis of global resting-state fMRI activity. Proc. Natl. Acad. Sci. U.S.A. 107, 10238–10243. 10.1073/pnas.0913110107 20439733 PMC2890438

[B438] Schöpf V. Windischberger C. Kasess C. H. Lanzenberger R. Moser E. (2010). Group ICA of resting-state data: a comparison. MAGMA 23, 317–325. 10.1007/s10334-010-0212-0 20521082

[B439] Schurz M. Wimmer H. Richlan F. Ludersdorfer P. Klackl J. Kronbichler M. (2015). Resting-state and task-based functional brain connectivity in developmental dyslexia. Cereb. Cortex 25, 3502–3514. 10.1093/cercor/bhu184 25169986 PMC4585499

[B440] Schwarzbach J. (2011). A simple framework (ASF) for behavioral and neuroimaging experiments based on the psychophysics toolbox for MATLAB. Behav. Res. Methods 43, 1194–1201. 10.3758/s13428-011-0106-8 21614662

[B441] Seshamani S. Blazejewska A. I. McKown S. Caucutt J. Dighe M. Gatenby C.. (2016). Detecting default mode networks *in utero* by integrated 4D *fMRI rec*onstruction and analysis. Hum. Brain Mapp. 37, 4158–4178. 10.1002/hbm.23303 27510837 PMC5557390

[B442] Seth A. K. Barrett A. B. Barnett L. (2015). Granger causality analysis in neuroscience and neuroimaging. J. Neurosci. 35, 3293–3297. 10.1523/JNEUROSCI.4399-14.2015 25716830 PMC4339347

[B443] Setsompop K. P. Polimeni J. R. Bhat H. Wald L. L. (2013). Characterization of artifactual correlation in highly-accelerated simultaneous multi-slice (SMS) fMRI acquisitions, in Proceedings of the 21st Annual Meeting of ISMRM (Salt Lake City, UT).

[B444] Shams S. M. Afshin-Pour B. Soltanian-Zadeh H. Hossein-Zadeh G. A. Strother S. C. (2015). Automated iterative reclustering framework for determining hierarchical functional networks in resting state fMRI. Hum. Brain Mapp. 36, 3303–3322. 10.1002/hbm.22839 26032457 PMC6869720

[B445] Shinkareva S. V. Wang J. Kim J. Facciani M. J. Baucom L. B. Wedell D. H. (2014). Representations of modality-specific affective processing for visual and auditory stimuli derived from functional magnetic resonance imaging data. Hum. Brain Mapp. 35, 3558–3568. 10.1002/hbm.22421 24302696 PMC6869138

[B446] Shirer W. R. Jiang H. Price C. M. Ng B. Greicius M. D. (2015). Optimization of rs-fMRI pre-processing for enhanced signal-noise separation, test-retest reliability, and group discrimination. Neuroimage 117, 67–79. 10.1016/j.neuroimage.2015.05.015 25987368

[B447] Sidhu M. K. Stretton J. Winston G. P. Symms M. Thompson P. J. Koepp M. J.. (2015). Memory fMRI predicts verbal memory decline after anterior temporal lobe resection. Neurology 84, 1512–1519. 10.1212/WNL.0000000000001461 25770199 PMC4408284

[B448] Siegel J. S. Power J. D. Dubis J. W. Vogel A. C. Church J. A. Schlaggar B. L.. (2014). Statistical improvements in functional magnetic resonance imaging analyses produced by censoring high-motion data points. Hum. Brain Mapp. 35, 1981–1996. 10.1002/hbm.22307 23861343 PMC3895106

[B449] Skouras S. Gray M. Critchley H. Koelsch S. (2014). Superficial amygdala and hippocampal activity during affective music listening observed at 3 T but not 1.5 T fMRI. Neuroimage 101, 364–369. 10.1016/j.neuroimage.2014.07.007 25026154

[B450] Sladky R. Friston K. J. Tröstl J. Cunnington R. Moser E. Windischberger C. (2011). Slice-timing effects and their correction in functional MRI. Neuroimage 58, 588–594. 10.1016/j.neuroimage.2011.06.078 21757015 PMC3167249

[B451] Smith S. M. Hyvärinen A. Varoquaux G. Miller K. L. Beckmann C. F. (2014). Group-PCA for very large fMRI datasets. Neuroimage 101, 738–749. 10.1016/j.neuroimage.2014.07.051 25094018 PMC4289914

[B452] Smith S. M. Miller K. L. Salimi-Khorshidi G. Webster M. Beckmann C. F. Nichols T. E.. (2011). Network modelling methods for FMRI. Neuroimage 54, 875–891. 10.1016/j.neuroimage.2010.08.063 20817103

[B453] Smith S. M. Nichols T. E. (2009). Threshold-free cluster enhancement: addressing problems of smoothing, threshold dependence and localisation in cluster inference. Neuroimage 44, 83–98. 10.1016/j.neuroimage.2008.03.061 18501637

[B454] Smith S. M. Vidaurre D. Beckmann C. F. Glasser M. F. Jenkinson M. Miller K. L.. (2013). Functional connectomics from resting-state fMRI. Trends Cogn. Sci. 17, 666–682. 10.1016/j.tics.2013.09.016 24238796 PMC4004765

[B455] Soares J. M. Marques P. Alves V. Sousa N. (2013). A hitchhiker's guide to diffusion tensor imaging. Front. Neurosci. 7:31. 10.3389/fnins.2013.00031 23486659 PMC3594764

[B456] Soares J. M. Marques P. Magalhães R. Santos N. C. Sousa N. (2016). The association between stress and mood across the adult lifespan on default mode network. Brain Struct. Funct. 10.1007/s00429-016-1203-3. [Epub ahead of print]. 26971253 PMC5225218

[B457] Soares J. M. Sampaio A. Ferreira L. M. Santos N. C. Marques F. Palha J. A.. (2012). Stress-induced changes in human decision-making are reversible. Transl. Psychiatry 2, e131. 10.1038/tp.2012.59 22760555 PMC3410630

[B458] Sporns O. (2014). Contributions and challenges for network models in cognitive neuroscience. Nat. Neurosci. 17, 652–660. 10.1038/nn.3690 24686784

[B459] Stam C. J. Reijneveld J. C. (2007). Graph theoretical analysis of complex networks in the brain. Nonlinear Biomed. Phys. 1, 3. 10.1186/1753-4631-1-3 17908336 PMC1976403

[B460] Stark C. E. L. Squire L. R. (2001). When zero is not zero: the problem of ambiguous baseline conditions in fMRI. Proc. Natl. Acad. Sci. U.S.A. 98, 12760–12766. 10.1073/pnas.221462998 11592989 PMC60127

[B461] Stefanovic B. Pike G. B. (2005). Venous refocusing for volume estimation: VERVE functional magnetic resonance imaging. Magn. Reson. Med. 53, 339–347. 10.1002/mrm.20352 15678548

[B462] Stehling M. K. Turner R. Mansfield P. (1991). Echo-planar imaging: magnetic resonance imaging in a fraction of a second. Science 254, 43–50. 10.1126/science.1925560 1925560

[B463] Steinbrink J. Villringer A. Kempf F. Haux D. Boden S. Obrig H. (2006). Illuminating the BOLD signal: combined fMRI-fNIRS studies. Magn. Reson. Imaging 24, 495–505. 10.1016/j.mri.2005.12.034 16677956

[B464] Stephan K. E. Kasper L. Harrison L. M. Daunizeau J. den Ouden H. E. Breakspear M.. (2008). Nonlinear dynamic causal models for fMRI. Neuroimage 42, 649–662. 10.1016/j.neuroimage.2008.04.262 18565765 PMC2636907

[B465] Stevens W. D. Buckner R. L. Schacter D. L. (2010). Correlated low-frequency BOLD fluctuations in the resting human brain are modulated by recent experience in category-preferential visual regions. Cereb. Cortex 20, 1997–2006. 10.1093/cercor/bhp270 20026486 PMC2901023

[B466] Stippich C. (2015). Clinical Functional MRI - Presurgical Functional Neuroimaging. Heidelberg: Springer.

[B467] Stöcker T. Schneider F. Klein M. Habel U. Kellermann T. Zilles K.. (2005). Automated quality assurance routines for fMRI data applied to a multicenter study. Hum. Brain Mapp. 25, 237–246. 10.1002/hbm.20096 15846770 PMC6871722

[B468] Stoewer S. Goense J. Keliris G. A. Bartels A. Logothetis N. K. Duncan J.. (2012). An analysis approach for high-field fMRI data from awake non-human primates. PLoS ONE 7:e29697. 10.1371/journal.pone.0029697 22238636 PMC3253095

[B469] Stroman P. W. Tomanek B. Krause V. Frankenstein U. N. Malisza K. L. (2003). Functional magnetic resonance imaging of the human brain based on signal enhancement by extravascular protons (SEEP fMRI). Magn. Reson. Med. 49, 433–439. 10.1002/mrm.10831 12594745

[B470] Strother S. C. (2006). Evaluating fMRI preprocessing pipelines. IEEE Eng. Med. Biol. Mag. 25, 27–41. 10.1109/MEMB.2006.1607667 16568935

[B471] Sulzer J. Haller S. Scharnowski F. Weiskopf N. Birbaumer N. Blefari M. L.. (2013). Real-time fMRI neurofeedback: progress and challenges. Neuroimage 76, 386–399. 10.1016/j.neuroimage.2013.03.033 23541800 PMC4878436

[B472] Sumiyoshi A. Suzuki H. Ogawa T. Riera J. J. Shimokawa H. Kawashima R. (2012). Coupling between gamma oscillation and fMRI signal in the rat somatosensory cortex: its dependence on systemic physiological parameters. Neuroimage 60, 738–746. 10.1016/j.neuroimage.2011.12.082 22245345

[B473] Tagliazucchi E. Laufs H. (2015). Multimodal imaging of dynamic functional connectivity. Front. Neurol. 6:10. 10.3389/fneur.2015.00010 25762977 PMC4329798

[B474] Talairach J. Tournoux P. (1988). Co-Planar Stereotaxic Atlas of the Human Brain: 3-D Proportional System: An Approach to Cerebral Imaging (Thieme Classics). New York, NY: Thieme.

[B475] Tana M. G. Sclocco R. Bianchi A. M. (2012). GMAC: a Matlab toolbox for spectral Granger causality analysis of fMRI data. Comput. Biol. Med. 42, 943–956. 10.1016/j.compbiomed.2012.07.003 22925560

[B476] Tanabe J. Miller D. Tregellas J. Freedman R. Meyer F. G. (2002). Comparison of detrending methods for optimal fMRI preprocessing. Neuroimage 15, 902–907. 10.1006/nimg.2002.1053 11906230

[B477] Telischak N. A. Detre J. A. Zaharchuk G. (2015). Arterial spin labeling MRI: clinical applications in the brain. J. Magn. Reson. Imaging 41, 1165–1180. 10.1002/jmri.24751 25236477

[B478] Tewarie P. Schoonheim M. M. Schouten D. I. Polman C. H. Balk L. J. Uitdehaag B. M.. (2015). Functional brain networks: linking thalamic atrophy to clinical disability in multiple sclerosis, a multimodal fMRI and MEG study. Hum. Brain Mapp. 36, 603–618. 10.1002/hbm.22650 25293505 PMC6869443

[B479] Thirion B. Pinel P. Mériaux S. Roche A. Dehaene S. Poline J. B. (2007). Analysis of a large fMRI cohort: Statistical and methodological issues for group analyses. Neuroimage 35, 105–120. 10.1016/j.neuroimage.2006.11.054 17239619

[B480] Tomarken A. J. Waller N. G. (2005). Structural equation modeling: strengths, limitations, and misconceptions. Annu. Rev. Clin. Psychol. 1, 31–65. 10.1146/annurev.clinpsy.1.102803.144239 17716081

[B481] Tomasi D. Shokri-Kojori E. Volkow N. D. (2016). Temporal changes in local functional connectivity density reflect the temporal variability of the amplitude of low frequency fluctuations in gray matter. PLoS ONE 11:e0154407. 10.1371/journal.pone.0154407 27116610 PMC4846007

[B482] Tousseyn S. Dupont P. Goffin K. Sunaert S. Van Paesschen W. (2015). Correspondence between large-scale ictal and interictal epileptic networks revealed by single photon emission computed tomography (SPECT) and electroencephalography (EEG)-functional magnetic resonance imaging (fMRI). Epilepsia 56, 382–392. 10.1111/epi.12910 25631544

[B483] Triantafyllou C. Hoge R. D. Krueger G. Wiggins C. J. Potthast A. Wiggins G. C.. (2005). Comparison of physiological noise at 1.5 T, 3 T and 7 T and optimization of fMRI acquisition parameters. Neuroimage 26, 243–250. 10.1016/j.neuroimage.2005.01.007 15862224

[B484] Triantafyllou C. Hoge R. D. Wald L. L. (2006). Effect of spatial smoothing on physiological noise in high-resolution fMRI. Neuroimage 32, 551–557. 10.1016/j.neuroimage.2006.04.182 16815038

[B485] Tsang O. Gholipour A. Kehtamavaz N. Gopinath K. Briggs R. (2007). Comparison of brain masking techniques in functional magnetic resonance imaging, in 2007 IEEE Dallas Engineering in Medicine and Biology Workshop (Dallas, TX), 78–81. 10.1109/EMBSW.2007.4454178

[B486] Tsvetanov K. A. Henson R. N. Tyler L. K. Davis S. W. Shafto M. A. Taylor J. R.. (2015). The effect of ageing on fMRI: Correction for the confounding effects of vascular reactivity evaluated by joint fMRI and MEG in 335 adults. Hum. Brain Mapp. 36, 2248–2269. 10.1002/hbm.22768 25727740 PMC4730557

[B487] Tung K.-C. Uh J. Mao D. Xu F. Xiao G. Lu H. (2013). Alterations in resting functional connectivity due to recent motor task. Neuroimage 78, 316–324. 10.1016/j.neuroimage.2013.04.006 23583747 PMC3672369

[B488] Tzourio-Mazoyer N. Landeau B. Papathanassiou D. Crivello F. Etard O. Delcroix N.. (2002). Automated anatomical labeling of activations in SPM using a macroscopic anatomical parcellation of the MNI MRI single-subject brain. Neuroimage 15, 273–289. 10.1006/nimg.2001.0978 11771995

[B489] Uğurbil K. Ogawa S. (2015). From BOLD contrast to imaging human brain function, in fMRI: From Nuclear Spins to Brain Functions, eds Uludag K. Ugurbil K. Berliner L. (Boston, MA: Springer US), 3–9.

[B490] Ulmer S. Jansen O. (2010). fMRI: Basics and Clinical Applications. Heidelberg: Springer 10.1007/978-3-540-68132-8

[B491] Uludag K. Roebroeck A. (2014). General overview on the merits of multimodal neuroimaging data fusion. Neuroimage 102(Pt 1), 3–10. 10.1016/j.neuroimage.2014.05.018 24845622

[B492] Vallesi A. Arbula S. Capizzi M. Causin F. D'Avella D. (2015). Domain-independent neural underpinning of task-switching: an fMRI investigation. Cortex 65C, 173–183. 10.1016/j.cortex.2015.01.016 25734897

[B493] van den Heuvel M. P. Hulshoff Pol H. E. (2010). Exploring the brain network: a review on resting-state fMRI functional connectivity. Eur. Neuropsychopharmacol. 20, 519–534. 10.1016/j.euroneuro.2010.03.008 20471808

[B494] van der Zwaag W. Francis S. Head K. Peters A. Gowland P. Morris P.. (2009). fMRI at 1.5, 3 and 7 T: characterising BOLD signal changes. Neuroimage 47, 1425–1434. 10.1016/j.neuroimage.2009.05.015 19446641

[B495] Van De Ville D. Seghier M. L. Lazeyras F. Blu T. Unser M. (2007). WSPM: wavelet-based statistical parametric mapping. Neuroimage 37, 1205–1217. 10.1016/j.neuroimage.2007.06.011 17689101

[B496] Van Dijk K. R. A. Hedden T. Venkataraman A. Evans K. C. Lazar S. W. Buckner R. L. (2010). Intrinsic functional connectivity as a tool for human connectomics: theory, properties, and optimization. J. Neurophysiol. 103, 297–321. 10.1152/jn.00783.2009 19889849 PMC2807224

[B497] Van Dijk K. R. Sabuncu M. R. Buckner R. L. (2012). The influence of head motion on intrinsic functional connectivity MRI. Neuroimage 59, 431–438. 10.1016/j.neuroimage.2011.07.044 21810475 PMC3683830

[B498] Van Essen D. C. Smith S. M. Barch D. M. Behrens T. E. Yacoub E. Ugurbil K.. (2013). The WU-Minn human connectome project: an overview. Neuroimage 80, 62–79. 10.1016/j.neuroimage.2013.05.041 23684880 PMC3724347

[B499] Van Horn J. D. Ishai A. (2007). Mapping the human brain: new insights from FMRI data sharing. Neuroinformatics 5, 146–153. 10.1007/s12021-007-0011-6 17917125

[B500] Van Horn J. D. Poldrack R. A. (2009). Functional MRI at the crossroads. Int. J. Psychophysiol. 73, 3–9. 10.1016/j.ijpsycho.2008.11.003 19041348 PMC2747289

[B501] Vaudano A. E. Carmichael D. W. Salek-Haddadi A. Rampp S. Stefan H. Lemieux L.. (2012). Networks involved in seizure initiation. A reading epilepsy case studied with EEG-fMRI and MEG. Neurology 79, 249–253. 10.1212/WNL.0b013e31825fdf3a 22764255 PMC3398433

[B502] Visscher K. M. Miezin F. M. Kelly J. E. Buckner R. L. Donaldson D. I. McAvoy M. P.. (2003). Mixed blocked/event-related designs separate transient and sustained activity in fMRI. Neuroimage 19, 1694–1708. 10.1016/S1053-8119(03)00178-2 12948724

[B503] Viviani R. Grön G. Spitzer M. (2005). Functional principal component analysis of fMRI data. Hum. Brain Mapp. 24, 109–129. 10.1002/hbm.20074 15468155 PMC6871761

[B504] Vuilleumier P. Armony J. L. Driver J. Dolan R. J. (2001). Effects of attention and emotion on face processing in the human brain: an event-related fMRI study. Neuron 30, 829–841. 10.1016/S0896-6273(01)00328-2 11430815

[B505] Vul E. Harris C. Winkielman P. Pashler H. (2009). Puzzlingly high correlations in fMRI studies of emotion, personality, and social cognition. Perspect. Psychol. Sci. 4, 274–290. 10.1111/j.1745-6924.2009.01125.x 26158964

[B506] Wager T. D. Jonides J. Reading S. (2004). Neuroimaging studies of shifting attention: a meta-analysis. Neuroimage 22, 1679–1693. 10.1016/j.neuroimage.2004.03.052 15275924

[B507] Wager T. D. Lindquist M. Kaplan L. (2007). Meta-analysis of functional neuroimaging data: current and future directions. Soc. Cogn. Affect. Neurosci. 2, 150–158. 10.1093/scan/nsm015 18985131 PMC2555451

[B508] Wager T. D. Nichols T. E. (2003). Optimization of experimental design in fMRI: a general framework using a genetic algorithm. Neuroimage 18, 293–309. 10.1016/S1053-8119(02)00046-0 12595184

[B509] Wald L. L. (2012). The future of acquisition speed, coverage, sensitivity, and resolution. Neuroimage 62, 1221–1229. 10.1016/j.neuroimage.2012.02.077 22421052 PMC3409932

[B510] Wang J. Wang X. Xia M. Liao X. Evans A. He Y. (2015). GRETNA: a graph theoretical network analysis toolbox for imaging connectomics. Front. Hum. Neurosci. 9:386. 10.3389/fnhum.2015.00386 26175682 PMC4485071

[B511] Wang J. Zuo X. He Y. (2010). Graph-based network analysis of resting-state functional MRI. Front. Syst. Neurosci. 4:16. 10.3389/fnsys.2010.00016 20589099 PMC2893007

[B512] Wang X.-H. Li L. Xu T. Ding Z. (2015). Investigating the temporal patterns within and between intrinsic connectivity networks under eyes-open and eyes-closed resting states: a dynamical functional connectivity study based on phase synchronization. PLoS ONE 10, e0140300. 10.1371/journal.pone.0140300 26469182 PMC4607488

[B513] Weber M. J. Messing S. B. Rao H. Detre J. A. Thompson-Schill S. L. (2014). Prefrontal transcranial direct current stimulation alters activation and connectivity in cortical and subcortical reward systems: a tDCS-fMRI study. Hum. Brain Mapp. 35, 3673–3686. 10.1002/hbm.22429 24453107 PMC4107089

[B514] Weiskopf N. (2012). Real-time fMRI and its application to neurofeedback. Neuroimage 62, 682–692. 10.1016/j.neuroimage.2011.10.009 22019880

[B515] Weiskopf N. Hutton C. Josephs O. Deichmann R. (2006). Optimal EPI parameters for reduction of susceptibility-induced BOLD sensitivity losses: a whole-brain analysis at 3 T and 1.5 T. Neuroimage 33, 493–504. 10.1016/j.neuroimage.2006.07.029 16959495

[B516] Welvaert M. Rosseel Y. (2013). On the definition of signal-to-noise ratio and contrast-to-noise ratio for FMRI data. PLoS ONE 8:e77089. 10.1371/journal.pone.0077089 24223118 PMC3819355

[B517] Wen X. Rangarajan G. Ding M. (2013). Is granger causality a viable technique for analyzing fMRI data? PLoS ONE 8:e67428. 10.1371/journal.pone.0067428 23861763 PMC3701552

[B518] White T. Muetzel R. Schmidt M. Langeslag S. J. Jaddoe V. Hofman A.. (2014). Time of acquisition and network stability in pediatric resting-state functional magnetic resonance imaging. Brain Connect. 4, 417–427. 10.1089/brain.2013.0195 24874884 PMC4120810

[B519] White T. O'Leary D. Magnotta V. Arndt S. Flaum M. Andreasen N. C. (2001). Anatomic and functional variability: the effects of filter size in group fMRI data analysis. Neuroimage 13, 577–588. 10.1006/nimg.2000.0716 11305887

[B520] Whitlow C. T. Casanova R. Maldjian J. A. (2011). Effect of resting-state functional MR imaging duration on stability of graph theory metrics of brain network connectivity. Radiology 259, 516–524. 10.1148/radiol.11101708 21406628

[B521] Williams D. S. Detre J. A. Leigh J. S. Koretsky A. P. (1992). Magnetic resonance imaging of perfusion using spin inversion of arterial water. Proc. Natl. Acad. Sci. U.S.A. 89, 212–216. 10.1073/pnas.89.1.212 1729691 PMC48206

[B522] Winkler A. M. Ridgway G. R. Webster M. A. Smith S. M. Nichols T. E. (2014). Permutation inference for the general linear model. Neuroimage 92, 381–397. 10.1016/j.neuroimage.2014.01.060 24530839 PMC4010955

[B523] Wise R. G. Preston C. (2010). What is the value of human FMRI in CNS drug development? Drug Discov. Today 15, 973–980. 10.1016/j.drudis.2010.08.016 20813202

[B524] Woermann F. G. Jokeit H. Luerding R. Freitag H. Schulz R. Guertler S.. (2003). Language lateralization by Wada test and fMRI in 100 patients with epilepsy. Neurology 61, 699–701. 10.1212/01.WNL.0000078815.03224.57 12963768

[B525] Wold S. Esbensen K. Geladi P. (1987). Principal component analysis. Chemometr. Intell. Lab. Syst. 2, 37–52. 10.1016/0169-7439(87)80084-927570801

[B526] Wong C. W. DeYoung P. N. Liu T. T. (2016). Differences in the resting-state fMRI global signal amplitude between the eyes open and eyes closed states are related to changes in EEG vigilance. Neuroimage 124(Pt A), 24–31. 10.1016/j.neuroimage.2015.08.053 26327245

[B527] Woo C. W. Krishnan A. Wager T. D. (2014). Cluster-extent based thresholding in fMRI analyses: pitfalls and recommendations. Neuroimage 91, 412–419. 10.1016/j.neuroimage.2013.12.058 24412399 PMC4214144

[B528] Worsley K. J. Marrett S. Neelin P. Vandal A. C. Friston K. J. Evans A. C. (1996). A unified statistical approach for determining significant signals in images of cerebral activation. Hum. Brain Mapp. 4, 58–73. 20408186 10.1002/(SICI)1097-0193(1996)4:1<58::AID-HBM4>3.0.CO;2-O

[B529] Wu C. W. Chen C. L. Liu P. Y. Chao Y. P. Biswal B. B. Lin C. P. (2011). Empirical evaluations of slice-timing, smoothing, and normalization effects in seed-based, resting-state functional magnetic resonance imaging analyses. Brain Connect. 1, 401–410. 10.1089/brain.2011.0018 22432454

[B530] Xu P. Huang R. Wang J. Van Dam N. T. Xie T. Dong Z.. (2014). Different topological organization of human brain functional networks with eyes open versus eyes closed. Neuroimage 90, 246–255. 10.1016/j.neuroimage.2013.12.060 24434242

[B531] Yan C. G. Wang X. D. Zuo X. N. Zang Y. F. (2016). DPABI: data processing & analysis for (resting-state) brain imaging. Neuroinformatics 14, 339–351. 10.1007/s12021-016-9299-4 27075850

[B532] Yan C.-G. Zang Y.-F. (2010). DPARSF: a matlab toolbox for “pipeline” data analysis of resting-state fMRI. Front. Syst. Neurosci. 4:13. 10.3389/fnsys.2010.00013 20577591 PMC2889691

[B533] Yan C. Liu D. He Y. Zou Q. Zhu C. Zuo X.. (2009). Spontaneous brain activity in the default mode network is sensitive to different resting-state conditions with limited cognitive load. PLoS ONE 4:e5743. 10.1371/journal.pone.0005743 19492040 PMC2683943

[B534] Yarkoni T. (2012). Sixteen is Not Magic: Comment on Friston (2012) [Online]. Available online at: http://www.talyarkoni.org/blog/2012/04/25/sixteen-is-not-magic-comment-on-friston-2012/

[B535] Ye J. Lazar N. A. Li Y. (2011). Sparse geostatistical analysis in clustering fMRI time series. J. Neurosci. Methods 199, 336–345. 10.1016/j.jneumeth.2011.05.016 21641934

[B536] Ye M. Yang T. Qing P. Lei X. Qiu J. Liu G. (2015). Changes of functional brain networks in major depressive disorder: a graph theoretical analysis of resting-state fMRI. PLoS ONE 10:e0133775. 10.1371/journal.pone.0133775 26327292 PMC4556670

[B537] Young K. D. Zotev V. Phillips R. Misaki M. Yuan H. Drevets W. C.. (2014). Real-time FMRI neurofeedback training of amygdala activity in patients with major depressive disorder. PLoS ONE 9:e88785. 10.1371/journal.pone.0088785 24523939 PMC3921228

[B538] Yue Y. Loh J. M. Lindquist M. A. (2010). Adaptive spatial smoothing of fMRI images. Stat. Interface 3, 3–13. 10.4310/SII.2010.v3.n1.a1

[B539] Zalesky A. Fornito A. Bullmore E. T. (2010). Network-based statistic: identifying differences in brain networks. Neuroimage 53, 1197–1207. 10.1016/j.neuroimage.2010.06.041 20600983

[B540] Zang Y. F. He Y. Zhu C. Z. Cao Q. J. Sui M. Q. Liang M.. (2007). Altered baseline brain activity in children with ADHD revealed by resting-state functional MRI. Brain Dev. 29, 83–91. 10.1016/j.braindev.2006.07.002 16919409

[B541] Zang Y. Jiang T. Lu Y. He Y. Tian L. (2004). Regional homogeneity approach to fMRI data analysis. Neuroimage 22, 394–400. 10.1016/j.neuroimage.2003.12.030 15110032

[B542] Zeng H. Constable R. T. (2002). Image distortion correction in EPI: comparison of field mapping with point spread function mapping. Magn. Reson. Med. 48, 137–146. 10.1002/mrm.10200 12111941

[B543] Zeng L. L. Wang D. Fox M. D. Sabuncu M. Hu D. Ge M.. (2014). Neurobiological basis of head motion in brain imaging. Proc. Natl. Acad. Sci. U.S.A. 111, 6058–6062. 10.1073/pnas.1317424111 24711399 PMC4000812

[B544] Zhan X. Yu R. (2015). A window into the brain: advances in psychiatric fMRI. Biomed Res. Int. 2015, 12. 10.1155/2015/542467 26413531 PMC4564608

[B545] Zhang D. Liang B. Wu X. Wang Z. Xu P. Chang S.. (2015). Directionality of large-scale resting-state brain networks during eyes open and eyes closed conditions. Front. Hum. Neurosci. 9:81. 10.3389/fnhum.2015.00081 25745394 PMC4333775

[B546] Zhong Y. Wang H. Lu G. Zhang Z. Jiao Q. Liu Y. (2009). Detecting functional connectivity in fMRI using PCA and regression analysis. Brain Topogr. 22, 134–144. 10.1007/s10548-009-0095-4 19408112

[B547] Zou Q. H. Zhu C. Z. Yang Y. Zuo X. N. Long X. Y. Cao Q. J.. (2008). An improved approach to detection of amplitude of low-frequency fluctuation (ALFF) for resting-state fMRI: fractional ALFF. J. Neurosci. Methods 172, 137–141. 10.1016/j.jneumeth.2008.04.012 18501969 PMC3902859

[B548] Zou Q. Miao X. Liu D. Wang D. J. Zhuo Y. Gao J. H. (2015). Reliability comparison of spontaneous brain activities between BOLD and CBF contrasts in eyes-open and eyes-closed resting states. Neuroimage 121, 91–105. 10.1016/j.neuroimage.2015.07.044 26226087

[B549] Zuo X. N. Ehmke R. Mennes M. Imperati D. Castellanos F. X. Sporns O.. (2012). Network centrality in the human functional connectome. Cereb. Cortex 22, 1862–1875. 10.1093/cercor/bhr269 21968567

